# N=N Bond Cleavage by Tantalum Hydride Complexes: Mechanistic Insights and Reactivity

**DOI:** 10.1021/acs.inorgchem.1c03152

**Published:** 2021-12-10

**Authors:** Elena Álvarez-Ruiz, Jorge J. Carbó, Manuel Gómez, Cristina Hernández-Prieto, Alberto Hernán-Gómez, Avelino Martín, Miguel Mena, Josep M. Ricart, Antoni Salom-Català, Cristina Santamaría

**Affiliations:** † Departamento de Química Orgánica y Química Inorgánica and Instituto de Investigación Química “Andrés M. del Río” (IQAR), Universidad de Alcalá, Campus Universitario, E-28805 Alcalá de Henares, Madrid, Spain; ‡ Departament de Química Física i Inorgànica, Universitat Rovira i Virgili, Campus Sescelades, C/Marcel.lí Domingo, s/n, 43007 Tarragona, Spain

## Abstract

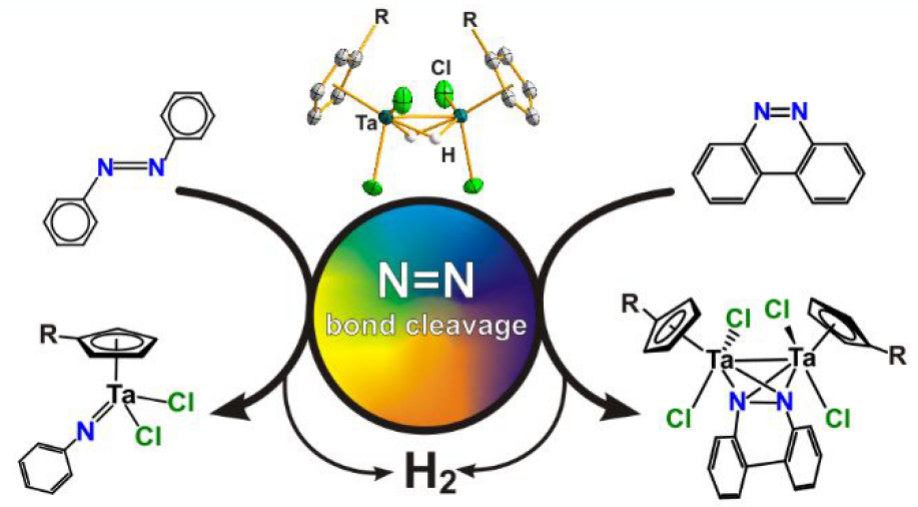

The reaction of [TaCp^R^X_4_] (Cp^R^ = η^5^-C_5_Me_5_, η^5^-C_5_H_4_SiMe_3_, η^5^-C_5_HMe_4_; X = Cl, Br) with SiH_3_Ph resulted in the formation of the dinuclear hydride tantalum(IV) compounds [(TaCp^R^X_2_)_2_(μ-H)_2_], structurally identified by single-crystal X-ray analyses. These species react with azobenzene to give the mononuclear imide complex [TaCp^R^X_2_(NPh)] along with the release of molecular hydrogen. Analogous reactions between the [{Ta(η^5^-C_5_Me_5_)X_2_}_2_(μ-H)_2_] derivatives and the cyclic diazo reagent benzo[*c*]cinnoline afford the biphenyl-bridged (phenylimido)tantalum complexes [{Ta(η^5^-C_5_Me_5_)X_2_}_2_(μ-NC_6_H_4_C_6_H_4_N)] along with the release of molecular hydrogen. When the compounds [(TaCp^R^X_2_)_2_(μ-H)_2_] (Cp^R^ = η^5^-C_5_H_4_SiMe_3_, η^5^-C_5_HMe_4_; X = Cl, Br) were employed, we were able to trap the side-on-bound diazo derivatives [(TaCp^R^X)_2_{μ-(η^2^,η^2^-NC_6_H_4_C_6_H_4_N)}] (Cp^R^ = η^5^-C_5_H_4_SiMe_3_, η^5^-C_5_HMe_4_; X = Cl, Br) as intermediates in the N=N bond cleavage process. DFT calculations provide insights into the N=N cleavage mechanism, in which the ditantalum(IV) fragment can promote two-electron reductions of the N=N bond at two different metal–metal bond splitting stages.

## Abstract 2

The series of dinuclear tantalum(IV) hydrides [{TaCp^R^X_2_}_2_(μ-H)_2_] (Cp^R^ = η^5^-C_5_Me_5_, η^5^-C_5_H_4_SiMe_3_, η^5^-C_5_HMe_4_; X = Cl, Br) show the ability to promote N=N bond cleavage in their reactions with azobenzene and benzo[*c*]cinnoline in absence of reducing reagents. Both the characterization of intermediate species and DFT studies point to a mechanism in two stages, in which the Ta−Ta bond splitting is key for the reduction of the N=N bond and its complete scission.

## Introduction

The study of N–N bond cleavage reactions is of great interest for the development of synthetic transformations using azo compounds as precursors of [NR] fragments^1−3^ and, more importantly, for a mechanistic understanding of the industrial and biological dinitrogen (N_2_) reduction to ammonia (NH_3_).^4,5^ Among the variety of metal compounds capable of promoting these reactions,^6^ low-valent early transition metals have attracted significant attention because they lead to metal imide fragments, which are important intermediates for a variety of catalytic processes such as nitrogen transfer, hydroamination, and metathesis reactions.^7^

The reduced metallic compounds required for the multielectron N–N scission process can be accessed by three different pathways. The first is the use of strong reductants such as alkaline and alkaline-earth metals, amalgams, alloys, and naphthalenides.^8^ This is nicely illustrated by the chemical reduction of [V(^*iPr*^BPDI)Cl_3_}] (^*iPr*^BPDI = 2,6-(2,6-*i*Pr_2_-C_6_H_3_N=CMe)_2_C_5_H_3_N) using sodium amalgam to form the vanadium dinitrogen complex [{V(^*iPr*^BPDI)(thf)}_2_(μ-N_2_)], which reacts with azobenzene to form a bis(imido) compound.^9^ In general terms, this approach has important limitations in the isolation of pure low-valent metal compounds, which are typically contaminated by the corresponding inorganic salts and over-reduced impurities. Improving this strategy, Mashima^10^ has reported a salt-free methodology employing a series of organic reducing reagents. This has been recently implemented in the formation of niobium and tantalum 2-pyridylimido compounds through the reaction of MCl_5_ (M = Nb, Ta), 2,2′-azopyridine, and 1-methyl-3,6-bis(trimethylsilyl)-1,4-cyclohexadiene as the reducing agent.^11^ Although this synthetic route succeeds in producing organometallic compounds with high purity, its use has been limited to halide and cyclopentadienyl group 4 and 5 derivatives.

Alternatively, metal hydride compounds have proved to be efficient precursors of low-valent species, H_2_ being the only byproduct generated.^12−15^ In line with the latter, Hou described the potential of his titanium hydride cluster [{Ti(η^5^-C_5_Me_4_SiMe_3_)}_3_(μ_3_-H)(μ-H)_6_] in the fixation and functionalization of N_2_.^16^

Notably, most of the investigations on N–N bond activation on azo compounds with low-valent early transition metals employ azobenzene as a model substrate and metallic reducing agents.^6^ On consideration of the advantages of metal hydride species, it is surprising that there has been only one example reported with a mid-valent transition metal. In this contribution, Holland et al. explored the ability of a high-spin iron(II) hydride dimer to break the N=N double bond in azo aromatics.^17^

Filling this gap for early transition metals, herein we report the N=N double-bond cleavage of the azo aromatics azobenzene and benzo[c]cinnoline, mediated by the series of dinuclear tantalum(IV) hydride complexes [(TaCp^R^X_2_)_2_(μ-H)_2_] (Cp^R^ = η^5^-C_5_Me_5_, η^5^-C_5_H_4_SiMe_3_, η^5^-C_5_HMe_4_; X = Cl, Br). Structurally mapping these reactions shows how the tantalum(IV) hydrides partially or totally break the N=N double bond with elimination of H_2_. In addition, insights into the electronic structures of dinuclear hydride tantalum(IV) compounds and a detailed atomistic description of the reaction mechanism are gained by theoretical studies.

## Results and Discussion

**Scheme 1:**
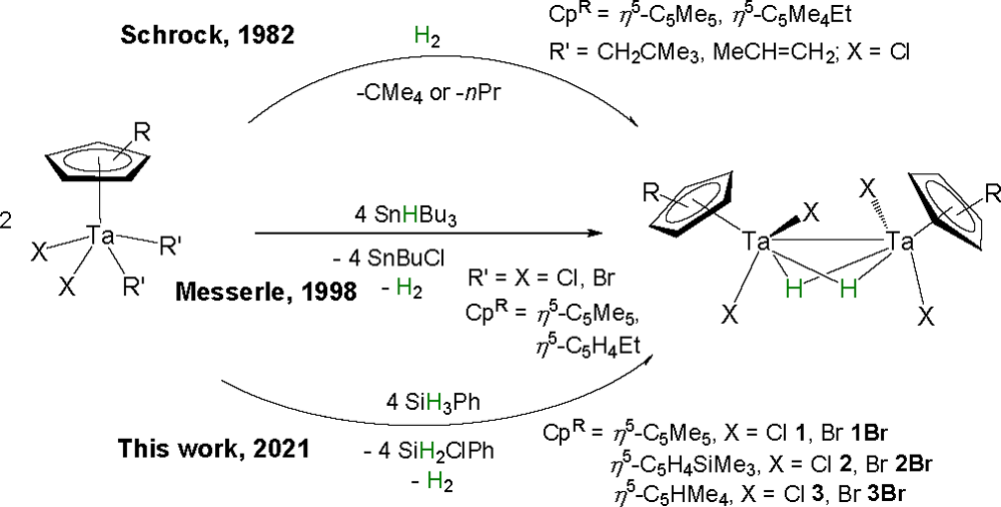
Synthetic Protocols of Tantalum(IV) Hydride Complexes

The preparation of the dinuclear hydride tantalum(IV) compound [{Ta(η^5^-C_5_Me_5_)Cl_2_}_2_(μ-H)_2_] (**1**) was first reported by Schrock and co-workers, via hydrogenation of both the bis(neopentyl) species [Ta(η^5^-C_5_Me_4_R)(CH_2_CMe_3_)_2_Cl_2_] (R = Me, Et) and the propylene complex [Ta(η^5^-C_5_Me_4_R)(MeCH=CH_2_)Cl_2_].^18^ Later, its synthesis was revisited by Messerle and co-workers, who prepared the hydride complex from the reaction of SnHBu_3_ with [Ta(η^5^-C_5_Me_4_R)Cl_4_] (R = Me, Et) (Scheme 1).^19^ Despite both groups providing a detailed characterization in solution, the determination of the solid-state structure was still elusive. We were delighted to observe that heating a toluene solution of SiH_3_Ph and [Ta(η^5^-C_5_Me_5_)Cl_4_] at 100 °C for 24 h affords, after slow cooling at room temperature, compound **1** as a crystalline solid in 92% yield (Scheme 1). Notably, this reaction is accompanied by a dramatic change in color from orange to dark blue.

This methodology is widely applicable, as evidenced by the synthesis of the series of hydride halo (chloro and bromo) tantalum species [(TaCp^R^X_2_)_2_(μ-H)_2_] (Cp^R^ = η^5^-C_5_Me_5_, η^5^-C_5_H_4_SiMe_3_, η^5^-C_5_HMe_4_; X = Cl, Br) (**1**–**3** and **1Br**–**3Br**) supported by different cyclopentadienyl derivatives. These compounds were isolated in high yields and characterized by X-ray diffraction analyses. For reasons of similarity in the synthetic protocols and the solid-state structures of the chloro and the bromo compounds, the experimental details and figures with the molecular structures of the latter derivatives are given in the Supporting Information.

**Figure 1:**
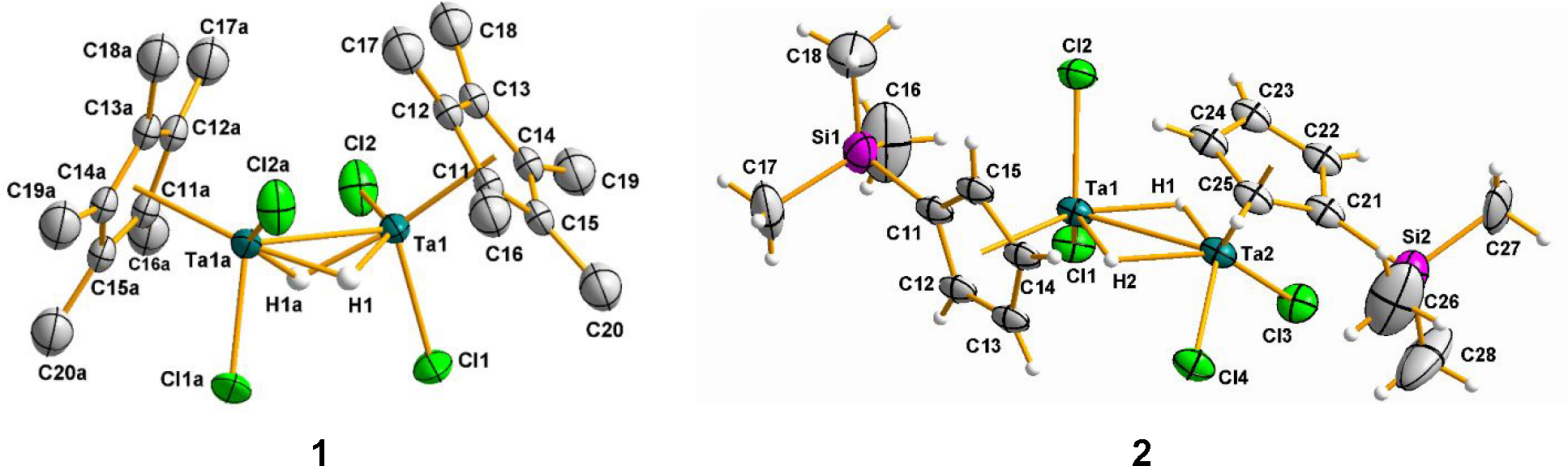
Molecular structures of compounds **1** and **2**. Thermal ellipsoids are shown at 50% probability. Hydrogen atoms of η^5^-C_5_Me_5_ ligands are omitted for clarity.

The solid-state structures of compounds **1** and **2** are depicted in Figure 1, while those of **1Br**–**3Br** can be found in Figures S1–S3 in the Supporting Information. Relevant bond distances and angles are given in Table 1. These products have a dinuclear structure with two bridging hydride units, consistent with the ^1^H NMR spectra. Both tantalum centers exhibit a distorted-trigonal-bipyramidal geometry comprising the centroid of the cyclopentadienyl ring, two chlorine/bromine atoms, and two hydride ligands, in some cases related by a crystallographic binary axis perpendicular to the Ta–Ta bond. The two tantalum atoms are separated by distances ranging from 2.753(2) to 2.813(1) Å, with the largest values being found for compounds **1** and **1Br** containing the bulkier pentamethylcyclopentadienyl ligand, which impedes a closer approximation between both metal centers. In line with this observation, the absence of cyclopentadienyl ligands in previously reported systems containing bridging hydrides results in slightly shorter metal–metal bond lengths.^20^ The Ta–Cl average bond length of 2.36(1) Å in complexes **1** and **2** is consistent with a Ta(IV)–Cl single bond, although it is slightly shorter than Ta–Cl distances found in the related ditantalum hydride complexes [{TaCl_2_(PMe_3_)_2_}_2_(μ-H)_2_] (average 2.418(3) Å)^20c^ and [{TaCl_2_(PMe_3_)_2_}_2_(μ-H)_4_] (2.461(5) Å).^20d^ Likewise, the Ta–Br average bond length of 2.505(4) Å in **1Br**–**3Br** is typical of single-bond distances. Additionally, the hydride atoms in these species were characterized as asymmetrically bridging the two tantalum atoms (see Table 1).

**Table 1:** Selected Average Lengths (Å) and Angles (deg) for Tantalum(IV) Hydride Complexes

	**1**	**1Br**	**2**	**2Br**	**3Br**
Ta–Ta	2.813(1)	2.840(2)	2.758(1)	2.753(2)	2.764(1)
Ta–H1	2.0(2)	1.960(1)	1.859(1)	1.86(2)	1.84(1)
Ta–H1a/2	1.8(2)	1.621(1)	1.656(1)	1.66(2)	1.85(1)
Ta–Cl/Br	2.36(1)	2.510(1)	2.354(9)	2.502(6)	2.503(8)
Ta–H–Ta	97(2)	104.5(1)	103.2(1)	102.5(5)	96.9(7)
Cl/Br–Ta–Cl/Br	100.0(2)	99.3(1)	101.5(3)	101.0(5)	101.5(1)

Although the positions of the hydride atoms in the diamagnetic compounds **1**, **1Br**, **2**, **2Br**, and **3Br** were determined in the difference Fourier map and their positions were refined, there is a slight uncertainty in these assignments due to the proximity of heavy tantalum and chlorine/bromine atoms that can overwhelm the small electron density of the H atoms. Complementing the X-ray data, the presence of bridging hydrides between the tantalum centers is also confirmed by infrared data (ν(TaH) stretching band at 1588 cm^–1^ (**1**), 1590 cm^–1^ (**1Br**), 1498 cm^–1^ (**2**), 1509 cm^–1^ (**2Br**), 1537 cm^–1^ (**3**), 1513 cm^–1^ (**3Br**)) and the highly upfield resonance found in the ^1^H NMR spectra (δ 10.23 (**1**), 11.24 (**1Br**), 10.05 (**2**), 10.79 (**2Br**), 10.16 (**3**), 10.97 (**3Br**)).

**Figure 2:**
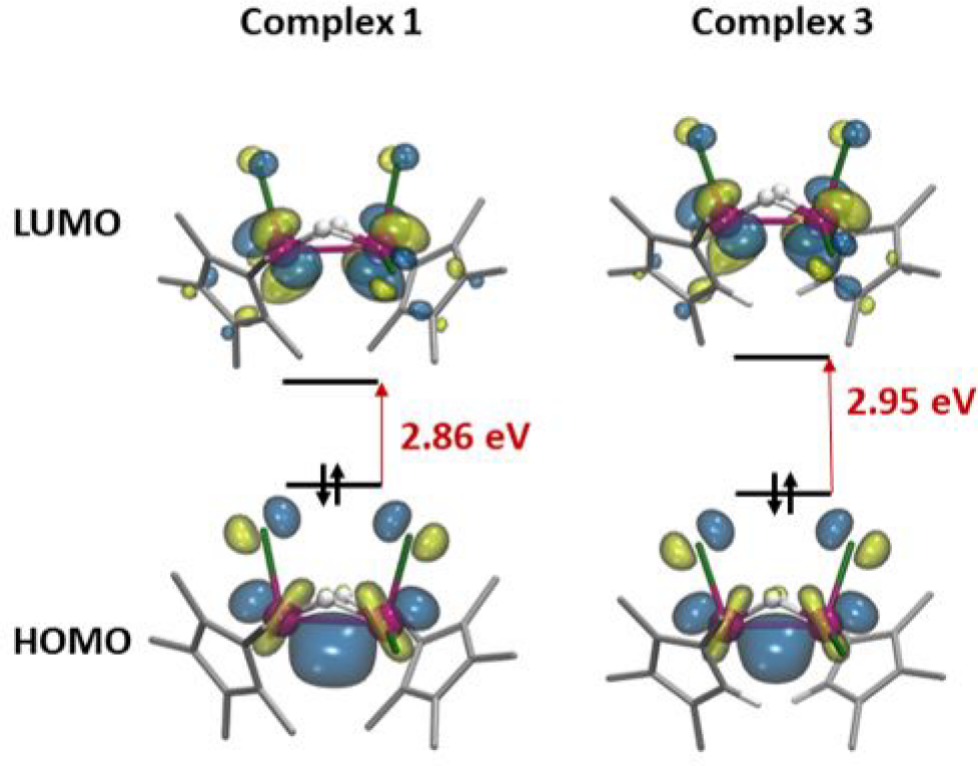
Frontier molecular orbitals of singlet state of complexes **1** and **3**.

These features led us to examine the electronic structure of these complexes by DFT calculations. Full geometric optimization of complexes **1**–**3** led to calculated geometric parameters in excellent agreement with the experimental values (see Table S2). Figure 2 shows the analysis of the frontier molecular orbitals (MOs) for complexes **1** and **3**, while Figure S60 shows the MOs of **2**. In all structures, the highest occupied molecular orbital (HOMO) consists of a bonding combination of atomic d-type orbitals centered at both tantalum centers, which is a clear indication of a σ Ta–Ta bonding between two Ta(IV) centers. The lowest unoccupied molecular orbital (LUMO) corresponds to a nonbonding orbital based on d-type orbitals centered at the Ta centers. The large energy gap between the HOMO and LUMO, ∼3 eV, further supports the experimental diamagnetic behavior.

**Scheme 2:**
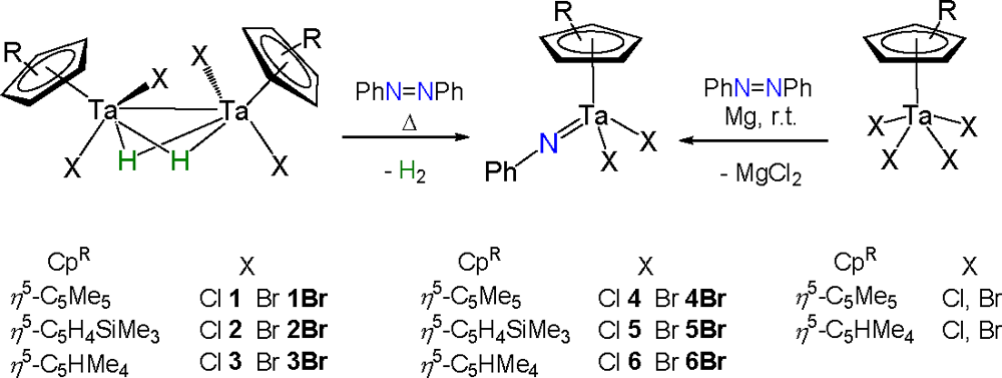
Synthetic Protocol of Tantalum(V) Imido Complexes **4**−**6** and **4Br**−**6Br**

To explore the potential of the hydride complexes in N=N bond cleavage, we began our studies using azobenzene. Thus, treatment of a dark blue toluene solution of [{Ta(η^5^-C_5_Me_5_)Cl_2_}_2_(μ-H)_2_] (**1**) with 1 equiv of azobenzene at room temperature yielded, after 12 h, a reddish orange solution formed by a mixture of products, from which only the mononuclear imide complex [Ta(η^5^-C_5_Me_5_)Cl_2_(NPh)] (**4**) could be unequivocally identified. Accordingly, single crystals grown from the latter reaction mixture allowed us to confirm the identity of compound **4**. In an improvement of the synthesis, a thermal treatment at 50 °C for 24 h of equimolar amounts of **1** and PhN=NPh renders compound **4** in high yield and purity (Scheme 2).

Since compound **4** has been previously reported, using a different synthetic protocol,^21^ details of the X-ray diffraction analysis are given in Figure S4 in the Supporting Information. Using the optimized reaction conditions, the imide complexes [TaCp^R^X_2_(NPh)] (Cp^R^ = η^5^-C_5_Me_5_, X = Br (**4Br**); Cp^R^ = η^5^-C_4_H_4_SiMe_3_, X = Cl (**5**), Br (**5Br**); Cp^R^ = η^5^-C_5_HMe_4_, X = Cl (**6**), Br (**6Br**)) were selectively formed upon reaction of the hydride bromo and chloro derivatives with azobenzene. Further studies reveal that the four-electron reduction and N=N bond cleavage of azobenzene to form compounds **4**, **4Br**, **6**, and **6Br** can be achieved in a one-pot reaction of the halo derivatives [TaCp^R^X_4_] (Cp^R^ = η^5^-C_5_Me_5_, η^5^-C_5_HMe_4_; X = Cl, Br), PhN=NPh, and magnesium in tetrahydrofuran.

The monomeric nature and the absence of hydride ligands in the solid-state structures of the complexes **4−6** and **4Br**−**6Br** suggest a metal-based four-electron reduction of azobenzene proceeding via Ta–Ta bond scission and reductive elimination of hydrogen. Similar metal–metal bond metathesis reductions of arylazo reagents to give imido fragments have been previously reported in the literature. Thus, Cotton and co-workers described the reaction of the M=M complexes [Cl_2_(R_2_S)M]_2_(μ-Cl)_2_(μ-SR_2_) (M = Nb,^22^ Ta^23^) with PhN=NPh to form dinuclear imido metal species. In a similar fashion, Tsurugi, Mashima, and co-workers reported the cleavage of the N=N double bond present in benzo[*c*]cinnoline by reaction with a W≡W triply bonded compound, (*t*BuO)_3_W≡W(O*t*Bu)_3_, affording dinuclear (imido)tungsten compounds.^24^ In contrast, the hydrogen via has been only explored by Holland and co-workers in the treatment of the low-coordinate iron(II) hydrides [L^MeEt^Fe(μ-H)]_2_ (L^MeEt^ = bulky β-diketiminate ligand) with PhN=NPh to give rise to an iron(III) imido dimer.^17^

**Scheme 3:**
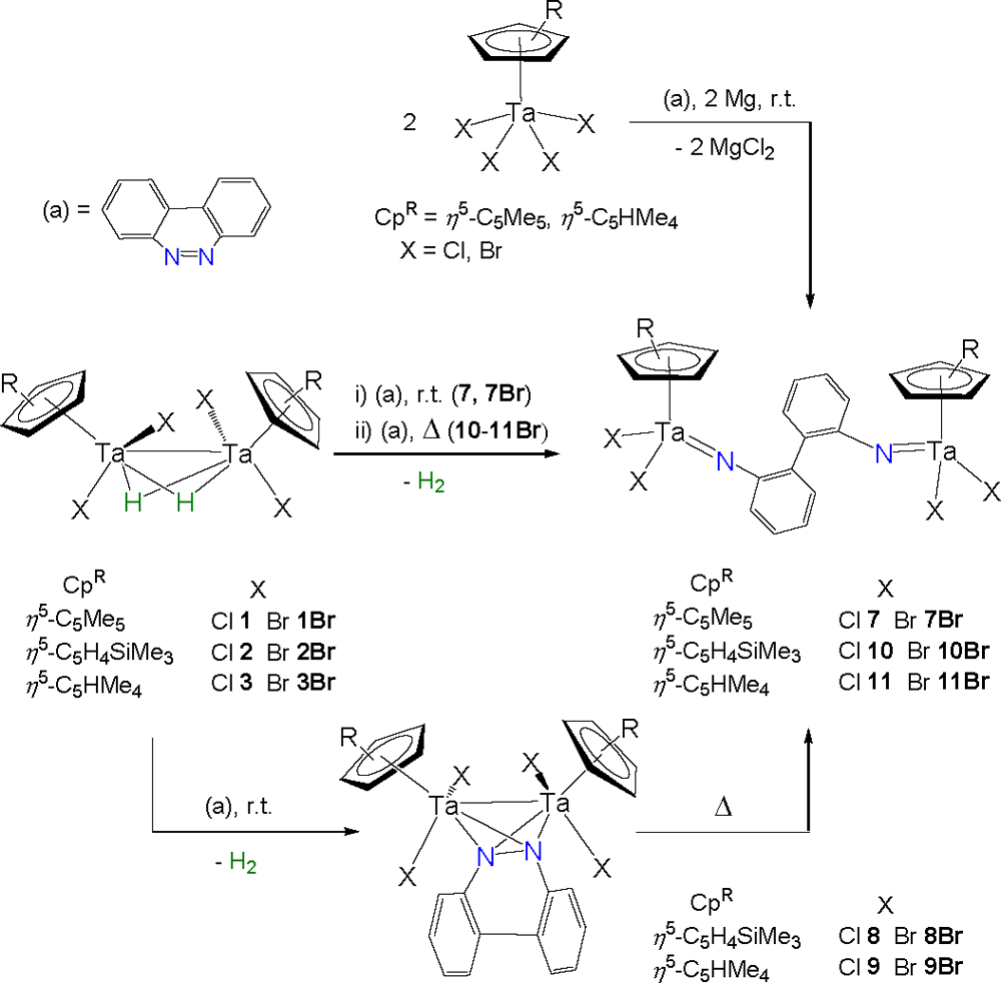
Reactions of the Hydride Tantalum Compounds **1**−**3**, **1Br**–**3Br**, and the Mononuclear Tetrahalo Derivatives with Benzo[*c*]cinnoline

Encouraged by these results, we expanded our studies toward N=N bond cleavage of the benzo[*c*]cinnoline. The treatment of complexes **1** and **1Br** with this diazo cyclic reagent in toluene at room temperature resulted in a dramatic color change from dark blue-green to orange-yellow to yield the aryl-imido complexes **7** and **7Br**, as outlined in Scheme 3. In contrast, the analogous reactions with 2, 2Br, 3, and 3Br led to the isolation of the dinuclear μ-η^2^,η^2^-benzo[*c*]cinnoline intermediates [(TaCp^R^X)_2_(μ-η^2^,η^2^-NC_6_H_4_C_6_H_4_N)] (Cp^R^ = η^5^-C_5_H_4_SiMe_3_, X = Cl (**8**), Br (**8Br**); Cp^R^ = η^5^-C_5_HMe_4_ X = Cl (**9**), Br (**9Br**)) as blue or violet products in high yields (>85%). Accordingly, the corresponding aryl-imido complexes **10**, **10Br**, **11**, and **11Br** could be obtained in high yield and purity by thermal treatment of the compounds **10**, **10Br**, **11**, and **11Br**. Consistent with a four-electron reduction process, the imido species **7**, **7Br**, **11**, and **11Br** were also formed by chemical reduction of the halo derivatives [TaCp^R^X_4_] (Cp^R^ = η^5^-C_5_Me_5_, η^5^-C_5_HMe_4_; X = Cl, Br) with magnesium in the presence of benzo[*c*]cinnoline, as outlined in Scheme 3.

In agreement with the formation of the diamagnetic and symmetrical biphenyl-bridged (phenylimido)tantalum(V) species **7**, **7Br**, **10**, **10Br**, **11**, and **11Br**, the ^1^H and ^13^C{^1^H} NMR spectra display one set of signals for the equivalent Cp^R^ ligands (Cp^R^ = η^5^-C_5_Me_5_, η^5^-C_5_H_4_SiMe_3_, η^5^-C_5_HMe_4_) and the aryl moiety.

**Figure 3:**
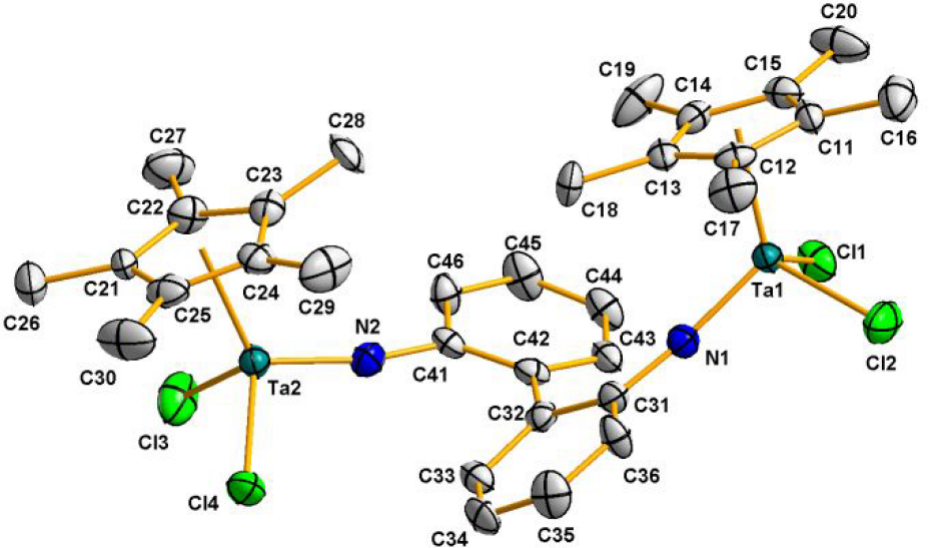
Molecular structure of **7**. Thermal ellipsoids are shown at 50% probability. Hydrogen atoms are omitted for clarity.

The identity of complex **7** as an aryl-imido complex was determined by a single-crystal X-ray diffraction study. The molecular structure of **7** is depicted in Figure 3, and a selection of the most relevant bond distances and angles is given in Table 2. Complex **7** contains two [Ta(η^5^-C_5_Me_5_)Cl_2_] units linked by a [1,1′-biphenyl]-2,2′-diimido bridge through the nitrogen atoms, confirming the complete cleavage of the N=N bond in benzo[*c*]cinnoline. The dihedral angle of the biphenyl moiety is 39.8(2)°, leading to a large separation of the tantalum atoms (7.962(1) Å). Ta–N bond distances (average value 1.790(1) Å) and Ta–N–C angles (average value 167.3(2)°) are within the ranges found for other cyclopentadienyl tantalum(V) imido complexes.^25^

**Table 2:** Selected Average Lengths (Å) and Angles (deg) for Tantalum(IV) Complexes **7**, **8Br**, and **9**

	**7**	**8Br**	**9**
Ta···Ta	7.962(1)		
Ta–Ta		2.917(1)	2.960(1)
Ta1–N1	1.791(7)	2.110(2)	2.110(7)
Ta1–N2		2.122(2)	2.156(7)
Ta2–N2	1.790(7)	2.116(2)	2.119(8)
Ta2–N1		2.119(2)	2.150(7)
Ta–Cl/Br	2.332(5)	2.53(2)	2.38(3)
N1–N2		1.450(3)	1.46(1)
N–C	1.385(5)	1.428(6)	1.43(1)
Ta–N–Ta		87.1(2)	87.8(2)

Intrigued by the formation of the intermediates **8**, **8Br**, **9**, and **9Br**, we monitored the reactions by ^1^H NMR spectroscopy, which display the release of molecular hydrogen (δ 4.46) and the presence of a symmetrical cyclopentadienyl tantalum tricyclic aryl moiety. The selective reduction of benzo[*c*]cinnoline by these compounds is in contrast with other potential four-electron or greater reductant species acting through bond metathesis, where the complete reduction and cleavage give the only observed product.^24^ In fact, these types of intermediates with benzo[*c*]cinnoline are usually accessed by employing two-electron-reducing derivatives such as those reported for niobium,^26^ iron,^27^ and zirconium.^28^

**Figure 4:**
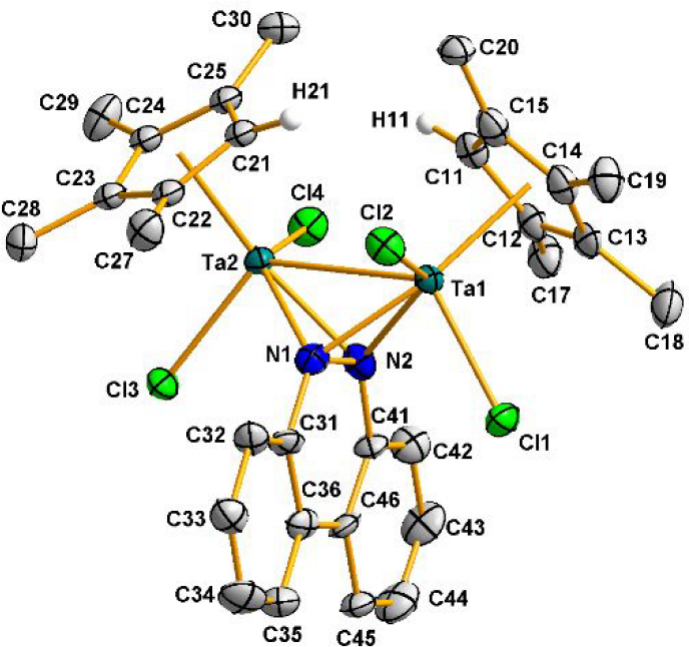
Molecular structure of **9**. Thermal ellipsoids are shown at 50% probability. Hydrogen atoms are omitted for clarity.

Complexes **8Br** and **9** were crystallized from hexane or toluene solutions, and the determination of their solid-state structures shows the geometry displayed in Scheme 3. Figure 4 depicts the molecular structure of **9**, while that of **8Br** can be found in Figure S5 in the Supporting Information. Relevant bond distances and angles are given in Table 2.

The structure of complexes **8Br** and **9** consists of two [TaCp^R^X_2_] (Cp^R^ = η^5^-C_5_H_4_SiMe_3_, X = Br; Cp^R^ = η^5^-C_5_HMe_4_; X = Cl) units bridged by a diazene ligand bound in a μ-η^2^,η^2^ fashion. The two-electron reduction of the N=N bond is reflected by the much larger bond distance (**8Br**, 1.450(3) Å; **9**, 1.46(1) Å) in comparison to that found for benzo[*c*]cinnoline (1.292(3) Å).^29^ A further analysis reveals that incorporation of the diamido fragment in the tantalum coordination sphere affects the Ta–Ta bond, which becomes slightly longer (2.917(1) Å (**8Br**) and 2.960(1) Å (**9**)) than those registered for the hydride starting materials **1**, **2**, and **1Br**−**3Br** (2.753(2)–2.813(1) Å).

The different chemical behavior of **2**, **2Br**, **3**, and **3Br** in comparison to **1** and **1Br** toward the activation of benzo[*c*]cinnoline can be ascribed to the observed difficulty in the formation of metal–metal bonds by the sterically demanding pentamethylcyclopentadienyl ligands, which would facilitate the formation of the final tantalum imides **7** and **7Br** without detection of the corresponding intermediate, according to the experimental results.

### Computational Characterization of the Mechanism of N=N Double-Bond Cleavage

**Figure 5:**
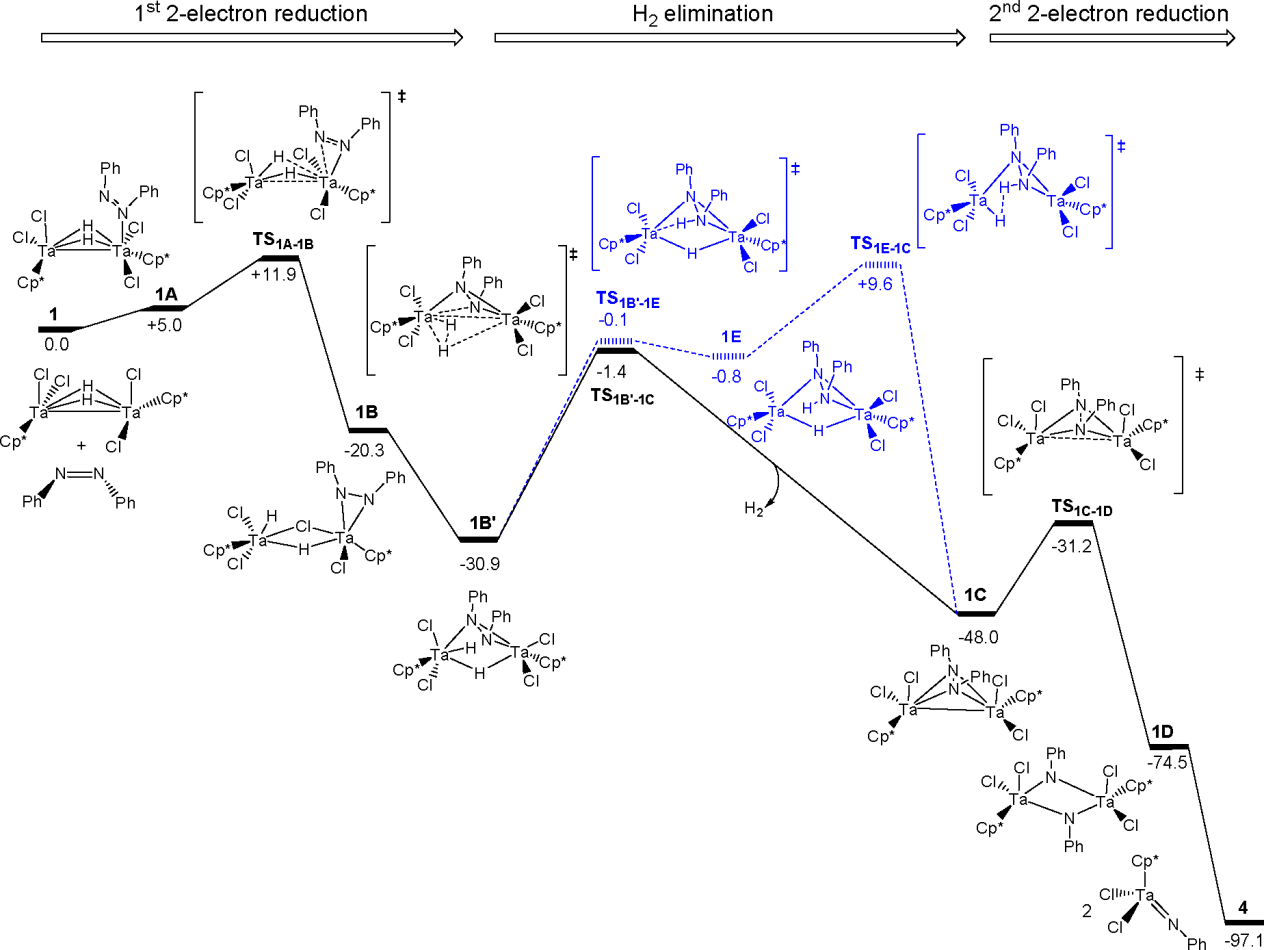
Gibbs free energy profile (kcal mol^–1^) for the reaction mechanism of **1** to **4** (solid black lines). An alternative, higher-energy mechanism is represented by blue lines.

**Figure 6:**
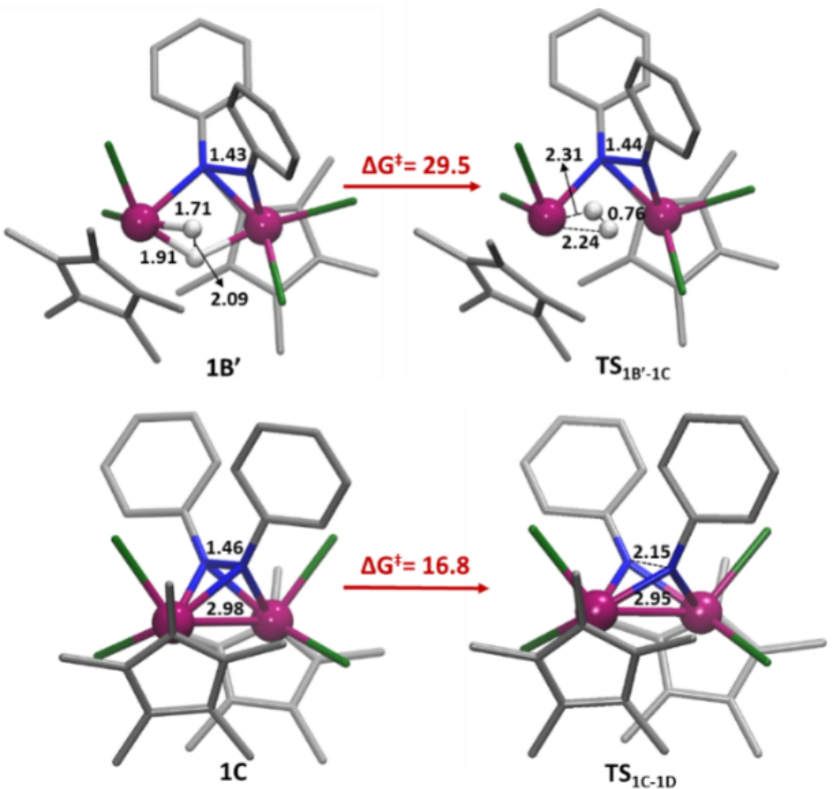
DFT structures of selected intermediates and transition states. Distances are given in Å and free energies in kcal mol^–1^.

To gain further insights into the four-electron N=N double-bond cleavage of azobenzene by the dinuclear Ta(IV) hydride complexes, we performed DFT calculations on the transformation of **1** to the imide complex [Ta(η^5^-C_5_Me_5_)Cl_2_(NPh)] (**4**). Figure 5 depicts the potential free energy profile of the proposed mechanism as solid black lines, and Figure 6 shows the most relevant structures. To facilitate the discussion, the mechanism is divided into three stages: (i) an initial two-electron reduction of azobenzene by Ta(IV)–Ta(IV) bond splitting and oxidation to yield the dinuclear Ta(V) hydride intermediate coordinating a (μ-η^2^:η^2^-PhN–NPh)^2–^ fragment, (ii) H_2_ reductive elimination to recover the Ta(IV) oxidation state and metal–metal bond, and (iii) a second two-electron reduction and N–N bond cleavage by virtue of the Ta(IV)–Ta(IV) bond scission and oxidation to form the Ta(V) imido complex **4**. This sequence is analogous to the mechanism proposed by Holland and co-workers for the azo N=N bond cleavage by an iron(II) hydride dimer, in which some intermediates were characterized computationally.^17^

For the first step, we have been able to identify several adducts resulting from the coordination of the diazobenzene to complex **1**; however, in all cases coordination required the *cis* configuration of the azobenzene, in which both phenyl substituents are pointing outside of the coordination sphere of the Ta dimer, to minimize the substrate–complex steric congestion. The *trans–cis* isomerization can occur photochemically and thermally, and the mechanisms have been extensively studied.^30^ In our case, we computed a free energy penalty of 12.7 kcal mol^–1^ for the isomerization, which is in good agreement with the experimental value reported, Δ*H* = 12–13 kcal mol^–1^.^31,32^ Among the variety of identified adducts, the most stable **1A** shows the azobenzene η^1^ coordinated to a single tantalum formed in a slightly endergonic (+5.0 kcal mol^–1^) process (see Figure 5). From **1A**, the azobenzene can undergo a two-electron reduction by Ta(IV)–Ta(IV) splitting and oxidation to Ta(V), overcoming a low free-energy barrier of 6.9 kcal mol^–1^ (Δ*G*^⧧^, **1A** → **TS**_**1A-1B**_). Overall, the first reduction toward formation of intermediate **1B** is an exergonic process with 25.3 kcal mol^–1^, which balances the energy penalty of *trans–cis* isomerization of diazobenzene and coordination (∼24 kcal mol^–1^). Intermediate **1B** is a tantalum(V) complex with a hydrazido ligand coordinated in a (η^2^-PhN–NPh)^2–^ fashion to only one of the metal centers. In addition, the calculated geometrical parameters for **1A** to **1B** reflect the partial cleavage of the N=N double bond to a single N–N bond and a change in the oxidation state from Ta(IV) to Ta(V) with concomitant cleavage of the metal–metal bond. Thus, the N–N and the Ta–Ta distances lengthen from 1.25 to 1.42 Å and from 2.81 to 3.51 Å, respectively. Further rearrangement gives rise to the isomer **1B′** that is more stable by 10.6 kcal mol^–1^, in which the hydrazide bridges the two metal centers (see Figures 5 and 6). It is interesting to note that metal hydrazide complexes are important intermediates in the development of dinitrogen activation^33,34^ and high-energy-density materials that release energy through dinitrogen evolution.^35^

Next, the reaction continues through H_2_ reductive elimination in **1B′** to form the intermediate **1C**, similar to the experimentally isolated dinuclear μ-η^2^,η^2^-benzo[*c*]cinnoline complexes (**8** and **9**). Indeed, the computed distances for Ta–Ta and N–N bonds in **1C** (2.98 and 1.46 Å, respectively) are very close to those determined by an X-ray study for complex **9** (2.960 and 1.46 Å, respectively). For the hydrogen evolution, we consider both homolytic and the heterolytic H–H bond formation as shown in Figure 5 (black and blue lines, respectively).^36^ With regard to the homolytic pathway (black line in Figure 5), it proceeds via the transition state **TS**_**1B′-1C**_, involving coupling of one terminal and one bridging hydride ligand of **1B′** to extrude H_2_ and reduce the metals to Ta(IV). In the resulting complex **1C**, the Ta–Ta distance is within the range of a metal–metal bond (2.98 Å) and the HOMO corresponds to a bonding orbital analogous to those represented in Figure 2. Accordantly, the Ta–Ta distance in the transition state **TS**_**1B′-1C**_ (3.17 Å) is significantly shorter that than in the reactant **1B′** (3.42 Å) (see Figure 6). Although this step has the largest free energy barrier along the proposed mechanism (29.5 kcal mol^–1^), the transformation of **1B′** to **1C** is an exergonic process driven by the very favorable thermodynamic nature of the H_2_ release process (Δ*G*(**1B′** → **1C**) = −17.1 kcal mol^–1^).

Alternatively, we have also examined the heterolytic reactivity across a polarized tantalum–hydride bond, which resembles that described for polynuclear titanium complexes activating strong bonds (N–H, C=O, or C–H) via addition across bridging Ti–X junctions (X = N, C).^37−40^ Moreover, a recent computational study proposes that the addition of dihydrogen to dinitrogen on a dimeric Ti(III)–hydride complex occurs without change in the oxidation state of titanium, while the subsequent reduction of the N–N bond involves oxidation to Ti(IV).^41^ In our case, a similar proposal implies the migration of a terminal hydride in **1B′** to one N atom of the hydrazide moiety to afford **1E**. On consideration that intermediate **1E** is 30.1 kcal mol^–1^ higher in energy than **1B′** and the subsequent heterolytic H–H bond formation, by reaction between the acidic proton of the NHPh fragment and the hydride ligand, requires an extra 10.4 kcal mol^–1^ (total value >40 kcal mol^–1^), this heterolytic pathway can be ruled out. We have also characterized another alternative heterolytic pathway, but it resulted in a more highly energy demanding process in comparison to that through the homolytic H_2_ elimination, and it did not explain the formation of the μ-η^2^,η^2^-aza intermediate observed experimentally (see Figure S61 in the Supporting Information).

The last stage of the reaction consists of a second two-electron reduction of the N–N bond of the μ-η^2^,η^2^-hydrazide by the ditantalum(IV) fragment in the intermediate **1C**. The process occurs with a moderate free energy barrier (Δ*G*^⧧^(**1C** → **TS**_**1C-1D**_) = 16.8 kcal mol^–1^) and causes the complete cleavage of the N–N bond to give the bis-imido Ta(V) complex **1D.** This bond cleavage is reflected in the evolution of N–N distances along the reaction coordinate, varying from 1.46 Å in **1C** to 2.15 Å in **TS**_**1C-1D**_ and then to 3.17 Å in **1D** (Figure 6). The formation of intermediate **1D** is notably exergonic (Δ*G*(**1C → 1D**) = −26.5 kcal mol^–1^), whereas its dissociation into two Ta(V) imido complexes **4** provides the thermodynamic driving force of the whole process, with product **4** lying 97.1 kcal mol^–1^ below the reactants.

## Conclusion

Combining SiH_3_Ph and the mononuclear tantalum(V) derivatives [TaCp^R^X_4_] (Cp^R^ = η^5^-C_5_Me_5_, η^5^-C_5_H_4_SiMe_3_, η^5^-C_5_HMe_4_; X = Cl, Br), we have developed a one-step methodology for the synthesis of dinuclear tantalum(IV) hydrides [(TaCp^R^X_2_)_2_(μ-H)_2_] (Cp^R^ = η^5^-C_5_Me_5_, η^5^-C_5_H_4_SiMe_3_, η^5^-C_5_HMe_4_; X = Cl, Br) in high yields. We have shown the ability of these species to promote a four-electron reduction and complete N=N bond cleavage in their reactions with azobenzene and benzo[*c*]cinnoline. Moreover, we were able to trap a side-on-bound μ-η^2^,η^2^-azo species in the reaction with the cyclic diazo reagent benzo[*c*]cinnoline as a plausible intermediate before cleaving the N=N bond.

A DFT analysis of the electronic structures of dinuclear tantalum(IV) hydrides complexes supports the metal–metal bonding between the two tantalum atoms, as manifested by the HOMO nature, consisting of a bonding combination of atomic d-type orbitals centered at both tantalum centers.

We have proposed a plausible reaction mechanism on the basis of DFT calculations that consists of three main steps: (i) initial two-electron reduction of diazabenzene by the hydride ditantalum(IV) fragment to yield the intermediate hydride ditantalum(V) coordinated by a (μ-η^2^:η^2^-PhN-NPh)^2–^ fragment, (ii) homolytic H_2_ evolution via reductive elimination to recover a dinuclear tantalum(IV) complex, and (iii) a second two-electron reduction by the ditantalum(IV) fragment, causing the complete cleavage of the N–N bond and yielding the tantalum(V) imido products.

## Experimental Section

### Experimental Details

All manipulations were carried out under a dry argon atmosphere using Schlenk-tube and cannula techniques or in a conventional argon-filled glovebox. Solvents were carefully refluxed over the appropriate drying agents and distilled prior to use: C_6_D_6_ and hexane (Na/K alloy), CDCl_3_ (CaH_2_), tetrahydrofuran (Na/benzophenone), and toluene (Na). The starting materials [TaCp^R^X_4_] (Cp^R^ = η^5^-C_5_Me_5_, η^5^-C_5_H_4_SiMe_3_; X = Cl, Br) were prepared by following the reported procedure for titanium,^42^ and the preparation of [Ta(η^5^-C_5_HMe_4_)Br_4_] is detailed in the Supporting Information along with the molecular structure of [Ta(η^5^-C_5_HMe_4_)Br_4_]. TaX_5_ (X = Cl, Br), SiH_3_Ph, and C_5_HMe_4_SiMe_3_ were purchased from Aldrich and were used without further purification. Azobenzene and benzo[*c*]cinnoline were purchased from Alfa Aesar and were used after sublimation. Microanalyses (C, H, N, S) were performed with a LECO CHNS-932 microanalyzer. Samples for IR spectroscopy were prepared as KBr pellets and spectra recorded on a PerkinElmer IR-FT Frontier spectrophotometer (4000–400 cm^–1^). ^1^H and ^13^C NMR spectra were obtained by using the Varian NMR spectrometers Unity-300 Plus, Mercury-VX, and Unity-500 and reported with reference to solvent resonances. ^1^H–^13^C gHSQC spectra were recorded using a Unity-500 MHz NMR spectrometer operating at 25 °C.

### Synthesis of [Ta(η^5^-C_5_HMe_4_)Cl_4_]

To a toluene (20 mL) suspension of TaCl_5_ (3.000 g, 8.375 mmol) placed in a Carius tube fitted with a Young valve was added dropwise C_5_HMe_4_SiMe_3_ (1.628 g, 8.375 mmol) dissolved in 10 mL of toluene. The mixture was stirred at 100 °C overnight and then evaporated to dryness. The residue was washed with two portions of hexane (40 mL) and dried in vacuo to give [Ta(η^5^-C_5_HMe_4_)Cl_4_] as a yellow solid (yield: 3.000 g, 81%). IR (KBr, cm^–1^): ν̃ 3085 (s, CH arom, 2996 (w, CH aliph), 2972 (w, CH aliph), 2922 (w, CH aliph), 1778 (w, CC), 1598 (m, CC), 1493 (m, CC), 1473 (m, CC), 1423 (m, CC), 1380 (s), 1018 (s), 887 (s), 605 (w), 433 (w). ^1^H NMR (300 MHz, C_6_D_6_): δ 5.45 (s, 1H, C_5_*H*Me_4_), 2.27, 2.03 (s, 6H, C_5_*Me*_4_H). ^13^C{^1^H} NMR (75 MHz, C_6_D_6_): δ 133.6, 132.7 (*C*-Me arom), 120.4 (*CH*_arom_), 15.8, 13.1 (C_5_H*Me*_4_). Anal. Calcd for C_9_H_13_Cl_4_Ta (443.96): C, 24.35; H, 2.95. Found: C, 24.07; H, 2.96.

### General Procedure for the Synthesis of [(TaCp^R^Cl_2_)_2_(μ-H)_2_] (Cp^R^ = η^5^-C_5_Me_5_ (**1**), η^5^-C_5_H_4_SiMe_3_ (**2**), η^5^-C_5_HMe_4_ (**3**))

A toluene (40–45 mL) solution of [TaCp^R^Cl_4_] (Cp^R^ = η^5^-C_5_Me_5_, η^5^-C_5_H_4_SiMe_3_; η^5^-C_5_HMe_4_) and SiH_3_Ph was placed in a Carius tube fitted with a Young valve, and the reaction mixture was stirred and heated. The resulting dark blue-green solutions were then filtered, and the solvent was removed under reduced pressure to afford microcrystalline dark blue-green solids. Crystals suitable for single crystal X-ray diffraction were obtained by cooling of the reaction mixture to room temperature.

#### Synthesis of [{Ta(η^5^-C_5_Me_5_)Cl_2_}_2_(μ-H)_2_] (**1**)

Thermal treatment at 100 °C for 24 h of a toluene solution of SiH_3_Ph (0.472 g, 4.367 mmol) and [Ta(η^5^-C_5_Me_5_)Cl_4_] (1.000 g, 2.183 mmol) gave complex **1** as a microcrystalline dark blue solid. (yield: 0.780 g, 92%). IR (KBr, cm^–1^): ν̃ 2966 (m, CH aliph), 2910 (m, CH aliph), 1588 (s, Ta–H), 1485 (m, CC), 1427 (m, CC), 1381 (s, CC), 1025 (m), 807 (w), 645 (w), 421 (w).

#### Synthesis of [{Ta(η^5^-C_5_H_4_SiMe_3_)Cl_2_}_2_(μ-H)_2_] (**2**)

Thermal treatment at 80 °C for 24 h of a toluene solution of SiH_3_Ph (0.471 g, 4.348 mmol) and [Ta(η^5^-C_5_H_4_SiMe_3_)Cl_4_] (1.000 g, 2.174 mmol) in toluene afforded complex **2** as a microcrystalline dark blue solid (yield: 0.740 g, 87%). IR (KBr, cm^–1^): ν̃ 3103 (w, CH ar) 2955 (m, CH aliph), 2897 (w, CH aliph), 1498 (w, Ta–H), 1399 (m, CC), 1251 (s, SiMe_3_), 1170 (m), 904 (m), 840 (vs, SiMe_3_), 760 (m, CH ar). ^1^H NMR (300 MHz, C_6_D_6_): δ 10.05 (s, 2H, Ta–*H*), 0.19 (s, 18H, C_5_H_4_S*iMe*_3_), not observed (C_5_*H*_4_SiMe_3_). ^13^C{^1^H} NMR (75 MHz, C_6_D_6_): δ −0.3 (s, C_5_H_4_Si*Me*_3_), not observed (*C*_5_H_4_SiMe_3_). Anal. Calcd for C_16_Cl_4_H_28_Si_2_Ta_2_ (780.27): C, 24.63; H, 3.62. Found: C, 24.80; H, 3.55.

#### Synthesis of [{Ta(η^5^-C_5_HMe_4_)Cl_2_}_2_(μ-H)_2_] (**3**)

Thermal treatment at 100 °C for 24 h of a toluene solution of SiH_3_Ph (0.487 g, 4.505 mmol) and [Ta(η^5^-C_5_HMe_4_)Cl_4_] (1.000 g, 2.252 mmol) afforded complex **3** as a microcrystalline dark blue solid (yield: 0.840 g, 99%). IR (KBr, cm^–1^): ν̃ 3085 (w, CH arom), 2986 (w, CH aliph), 2964 (w, CH aliph), 2918 (m, CH aliph), 2859 (w, CH aliph), 1537 (s, Ta–H), 1483 (s, CC), 1382 (s, CC), 1321 (w, CC), 1150 (w), 1024 (m), 870 (m). ^1^H NMR (300 MHz, C_6_D_6_): δ 10.16 (s, 2H, Ta–*H*), 7.45 (s, 2H, C_5_*H*Me_4_), 2.17 (bs, 12H, C_5_H*Me*_4_), 1.65 (s, 12H, C_5_H*Me*_4_). ^13^C{^1^H} NMR (75 MHz, C_6_D_6_): δ 126.2 (*C*_5_HMe_4_), 123.3 (bs, *C*_5_HMe_4_), 99.0 (*C*_5_HMe_4_), 13.9 (bs, C_5_H*Me*_4_), 11.2 (C_5_H*Me*_4_). Anal. Calcd for C_18_H_28_Cl_4_Ta_2_ (748.12): C, 28.89; H, 3.77. Found: C, 29.10; H, 3.78.

### General Procedure for the Synthesis of [TaCp^R^Cl_2_(NPh)] (Cp^R^ = η^5^-C_5_Me_5_ (**4**), η^5^-C_5_H_4_SiMe_3_ (**5**), η^5^-C_5_HMe_4_ (**6**))

Ph_2_N_2_ was added to a toluene solution (30–40 mL) of [(TaCp^R^X_2_)_2_(μ-H)_2_] (Cp^R^ = η^5^-C_5_Me_5_, η^5^-C_5_H_4_SiMe_3_; η^5^-C_5_HMe_4_) placed in a Carius tube (100 mL) with a Young valve. The argon pressure was reduced, and the reaction mixture was stirred and heated for 24–48 h and then filtered. The solvent was removed under reduced pressure to afford microcrystalline orange solids (**4**, **6**) or a dark orange oil (**5**).

#### Synthesis of [Ta(η^5^-C_5_Me_5_)Cl_2_(NPh)] (**4**)

The thermal treatment at 50 °C for 24 h of a toluene solution of Ph_2_N_2_ (0.117 g, 0.644 mmol) and [{Ta(η^5^-C_5_Me_5_)Cl_2_}_2_(μ-H)_2_] (**1**; 0.500 g, 0.644 mmol) in toluene afforded complex **4** (yield: 0.477 g, 78%).

#### Synthesis of [Ta(η^5^-C_5_H_4_SiMe_3_)Cl_2_(NPh)] (**5**)

The thermal treatment at 60 °C for 24 h of a toluene solution of Ph_2_N_2_ (0.117 g, 0.641 mmol) and [{Ta(η^5^-C_5_H_4_SiMe_3_)Cl_2_}_2_(μ-H)_2_] (**2**; 0.500 g, 0.641 mmol) in toluene afforded compound **5** (yield: 0.560 g, 91%). IR (KBr, cm^–1^): ν̃ 3102 (w, CH arom), 2955 (w, CH aliph), 2897 (w, CH aliph), 1584 (w, CC), 1483 (s, CC), 1351 (m, =NR), 1251 (s, SiMe_3_), 1171 (m), 841 (vs, SiMe_3_), 760 (s), 692 (m). ^1^H NMR (300 MHz, C_6_D_6_): δ 7.09, 6.83, 6.72 (m, 5H, N*Ph*), 6.20, 5.93 (spin system AA′BB′, 4H, C_5_*H*_4_SiMe_3_), 0.12 (s, 9H, C_5_H_4_Si*Me*_3_). ^13^C{^1^H} NMR (75 MHz, C_6_D_6_): δ 156.0 (C_ipso_ N*Ph*), 128.1, 125.9, 125.3 (N*Ph*), 120.7, 113.9 (*C*_5_H_4_SiMe_3_), −0.6 (C_5_H_4_Si*Me*_3_). Anal. Calcd for C_14_H_18_NSiCl_2_Ta (480.24): C, 35.01; H, 3.78; N, 2.92. Found: C, 34.81; H, 3.61; N, 3.51.

#### Synthesis of [Ta(η^5^-C_5_HMe_4_)Cl_2_(NPh)] (**6**)

##### Method A

Thermal treatment at 70 °C for 24 h of a toluene solution of Ph_2_N_2_ (0.097 g, 0.535 mmol) and [{Ta(η^5^-C_5_HMe_4_)Cl_2_}_2_(μ-H)_2_] (**3**; 0.400 g, 0.535 mmol) afforded complex **6** (yield: 0.447 g, 90%).

##### Method B

A 100 mL Schlenk vessel was charged in the glovebox with Ph_2_N_2_ (0.082 g, 0.450 mmol), [Ta(η^5^-C_5_Me_4_H)Cl_4_] (0.400 g, 0.901 mmol,), Mg (0.022 g, 0.901 mmol), and thf (30–40 mL). After it was stirred for 24 h at room temperature, the reaction mixture was dried under vacuum, the product was extracted with toluene, the extract was filtered through a medium-porosity glass frit, and the solvent was then removed under vacuum to give complex **6** as an orange solid (yield: 0.35 g, 85%). IR (KBr, cm^–1^): ν̃ 3086 (w, CH arom, 3070 (w, CH arom), 2980 (w, CH aliph), 2963 (w, CH aliph), 2916 (w, CH aliph), 1603 (w, CC), 1585 (m, CC), 1495 (s, CC), 1482 (m, CC), 1381 (m, CC), 1347 (s, =NR), 1068 (s), 766 (m), 693 (s). ^1^H NMR (500 MHz, C_6_D_6_): δ 7.20–6.60 (m, 5H, NPh), 5.37 (s, 1H, C_5_*H*Me_4_), 1.90, 1.77 (s, 6H, C_5_H*Me*_4_). ^13^C{^1^H} NMR (125 MHz, C_6_D_6_): δ 126.2, 125.1, 124.6, 122.1 (Ph), 107.3 (overlapped, *C*_5_HMe_4_), 13.2, 10.9 (C_5_H*Me*_4_). Anal. Calcd for C_15_H_18_Cl_2_NTa (464.16): C, 38.81; H, 3.91; N, 3.02. Found: C, 38.87; H, 3.92; N, 3.81.

#### Synthesis of [{Ta(η^5^-C_5_Me_5_)Cl_2_}_2_(μ-NC_6_H_4_–C_6_H_4_N)] (**7**)

A 100 mL Schlenk vessel was charged in the glovebox with benzo[*c*]cinnoline (0.116 g, 0.644 mmol), [{Ta(η^5^-C_5_Me_5_)Cl_2_}_2_(μ-H)_2_] (**1**; 0.500 g, 0.644 mmol), and toluene (30–40 mL). After it was stirred for 24 h at room temperature, the reaction mixture was filtered through a medium-porosity glass frit, and the solvent was then removed under vacuum to give **7** (yield: 0.516 g, 84%) as a microcrystalline orange solid. IR (KBr, cm^–1^): ν̃ 3054 (w, CH arom, 2982 (w, CH aliph), 2918 (m, CH aliph), 1584 (w, CC), 1458 (m, CC), 1420 (s, CC), 1335 (s, =NR), 1113 (w), 976 (m), 760 (s), 535 (m). ^1^H NMR (500 MHz, C_6_D_6_): δ 7.50–6.80 (m, 8H, NC_6_*H*_4_–C_6_*H*_4_N), 1.88 (s, 30H, C_5_*Me*_5_). ^13^C{^1^H} NMR (125 MHz, C_6_D_6_): δ 152.0, 135.7, 132.5, 128.7, 127.0, 124.2, (NC_6_H_4_-C_6_H_4_N), 121.6 (*C*_5_Me_5_), 11.6 (C_5_*Me*_5_). Anal. Calcd for C_32_Cl_4_H_38_N_2_Ta_2_ (954.36): C, 40.27; H, 4.01; N, 2.93. Found: C, 39.85; H, 4.03; N, 3.78.

### General Procedure for the Synthesis of [{TaCp^R^Cl_2_}(μ-η^2^,η^2^-NC_6_H_4_–C_6_H_4_N)] (Cp^R^ = η^5^-C_5_H_4_SiMe_3_ (**8**), η^5^-C_5_HMe_4_ (**9**))

A 100 mL Schlenk vessel was charged in the glovebox with benzo[*c*]cinnoline, [(TaCp^R^Cl_2_)_2_(μ-H)_2_] (Cp^R^ = η^5^-C_5_H_4_SiMe_3_; η^5^-C_5_HMe_4_), and toluene. After it was stirred at room temperature for 24 h, the reaction mixture was filtered through a medium-porosity glass frit, and the solvent was then removed under vacuum to yield microcrystalline blue-purple solids.

#### Synthesis of [{Ta(η^5^-C_5_H_4_SiMe_3_)Cl_2_}_2_(μ-η^2^,η^2^-NC_6_H_4_C_6_H_4_N)] (**8**)

A 100 mL Schlenk vessel was charged with benzo[*c*]cinnoline (0.115 g, 0.641 mmol), [{Ta(η^5^-C_5_H_4_SiMe_3_)Cl_2_}_2_(μ-H)_2_] (**2**; 0.500 g, 0.641 mmol), and toluene (30–40 mL) to give **8** (yield: 0.550 g, 89%) as a blue microcrystalline solid. IR (KBr, cm^–1^): ν̃ 3076 (w, CH arom, 2954 (m, CH aliph), 2897 (w, CH aliph), 1601 (w), 1481 (m, CC), 1437 (m, CC), 1253 (s, SiMe_3_), 1169 (m), 903 (m), 840 (vs, SiMe_3_), 758 (s). ^1^H NMR (300 MHz, C_6_D_6_): δ 7.69–6.80 (m, 8H, NC_6_*H*_4_–C_6_*H*_4_N), 7.62–5.47 (m, 8 H, C_5_*H*_4_SiMe_3_), 0.20 (s, 18 H, C_5_H_4_Si*Me*_3_). ^13^C{^1^H} NMR (75 MHz, C_6_D_6_): δ 151.1, 128.5, 126.9, 125.7, 122.2, 120.7 (N*C*_6_H_4_–*C*_6_H_4_N) 124.5, 119.4, 115.4, 110.3 (*C*_5_H_4_SiMe_3_), −0.3 (C_5_H_4_Si*Me*_3_). Anal. Calcd for C_28_H_34_Cl_4_N_2_Si_2_Ta_2_ (958.46): C, 35.09; H, 3.57; N, 2.92. Found: C, 34.79; H, 3.63; N, 3.49.

#### Synthesis of [{Ta(η^5^-C_5_HMe_4_)Cl_2_}_2_(μ-η^2^,η^2^-NC_6_H_4_C_6_H_4_N)] (**9**)

##### Method A

A 100 mL Schlenk vessel was charged with benzo[*c*]cinnoline (0.072 g, 0.401 mmol), [{Ta(η^5^-C_5_HMe_4_)Cl_2_}_2_(μ-H)_2_] (**3**; 0.300 g, 0.401 mmol), and toluene (30–40 mL) to give **9** (yield: 0.327 g, 88%) as a purple microcrystalline solid.

##### Method B

A 100 mL Schlenk vessel was charged in the glovebox with benzo[*c*]cinnoline (0.081 g, 0.450 mmol), [Ta(η^5^-C_5_HMe_4_)Cl_4_] (0.400 g, 0.901 mmol), Mg (0.022 g, 0.901 mmol), and thf (30–40 mL). After it was stirred for 24 h at room temperature, the reaction mixture was dried under vacuum, the product was extracted with toluene, the extract was filtered through a medium-porosity glass frit, and the solvent was then removed under vacuum to give complex **9** as a purple solid (yield: 0.300 g, 72%). IR (KBr, cm^–1^): ν̃ 3033 (w, CH arom, 2999 (w, CH aliph), 2963 (w, CH aliph), 2912 (w, CH aliph), 1601 (m, CC), 1502 (s, CC), 1492 (s, CC), 1484 (s, CC), 1377 (m, Ta–N), 1256 (s), 1105 (m), 974 (m), 757 (s), 468 (m). ^1^H NMR (500 MHz, C_6_D_6_): δ 7.93 (s, 2H, C_5_HMe_4_), 7.90–6.80 (m, 8H, NC_6_H_4_–C_6_H_4_N), 2.57, 2.12, 1.78, 1.47 (s, 6H, C_5_HMe_4_). ^13^C{^1^H} NMR (125 MHz, C_6_D_6_): δ 151.9 132.3, 125.4, 122.1, 122.0, 119.4 (NC_6_H_4_–C_6_H_4_N), 100.8 (*C*_5_HMe_4_), 15.7, 13.0, 12.1, 11.4 (C_5_H*Me*_4_). Anal. Calcd for C_30_H_34_Cl_4_N_2_Ta_2_ (926.31): C, 38.90; H, 3.70; N, 3.02. Found: C, 38.87; H, 3.92; N, 3.81.

### Synthesis of [{Ta(η^5^-C_5_H_4_SiMe_3_)Cl_2_}_2_(μ-NC_6_H_4_–C_6_H_4_N)] (**10**)

A toluene solution (20 mL) of complex **8** (0.400 g, 0.417 mmol) was placed in a 25 mL Carius tube and then sealed under vacuum by flame. The reaction mixture was heated in an autoclave at 200 °C for 3 days, and then was cooled to room temperature affording an orange oil. The Carious tube was opened in a glovebox, the solution was decanted, and the solvent was then removed in vacuum to yield **10** (0.38 g, 95%). IR (KBr, cm^–1^): ν̃ 3088 (w, CH arom, 2953 (m, CH aliph), 2895 (m, CH aliph), 1564 (w), 1485 (m, CC), 1435 (m, CC), 1342 (m, =NR), 1250 (s, SiMe_3_), 904 (m), 840 (vs, SiMe_3_), 758 (s). ^1^H NMR (300 MHz, C_6_D_6_): 7.36–6.80 (m, 8H, NC_6_*H*_4_-C_6_*H*_4_N), 6.21–5.92 (spin system AA′BB′, m, 8 H, C_5_*H*_4_SiMe_3_), 0.18 (s, 18 H, C_5_H_4_Si*Me*_3_). ^13^C{^1^H} NMR (75 MHz, C_6_D_6_): δ 154.3, 136.7, 131.7, 127.3, 127.1, 124.8 (N*C*_6_H_4_-*C*_6_H_4_N), 121.5, 113.2 (*C*_5_H_4_SiMe_3_), −0.6 (C_5_H_4_Si*Me*_3_) Anal. Calcd for C_28_H_34_Cl_4_N_2_Si_2_Ta_2_ (958.46): C, 35.09; H, 3.57; N, 2.92 found: C, 35.01; H, 3.46; N, 3.24.

### Synthesis of [{Ta(η^5^-C_5_HMe_4_)Cl_2_}_2_(μ-NC_6_H_4_–C_6_H_4_N)] (**11**)

A Carius tube (100 mL) with a Young valve was charged with benzo[*c*]cinnoline (0.072 g, 0.401 mmol), toluene (30–40 mL), and [{Ta(η^5^-C_5_HMe_4_)Cl_2_}_2_(μ-H)_2_] (**3**) (0.300 g, 0.401 mmol), and the mixture was heated to 90 °C for 24 h to give **11** (yield: 0.267 g, 72%). IR (KBr, cm^–1^): ν̃ 3050 (w, CH arom, 2976 (w, CH aliph), 2962 (w, CH aliph), 2918 (w, CH aliph), 1585 (w, CC), 1486 (m, CC), 1449 (m, CC), 1424 (m, CC), 1342 (s, =NR), 1112 (w), 1105 (m), 763 (s). ^1^H NMR (300 MHz, C_6_D_6_): δ 7.50–6.80 (m, 8H, NC_6_*H*_4_–C_6_*H*_4_N), 5.51 (s, 2H, C_5_*H*Me_4_), 1.99, 1.76 (s, 12H, C_5_H*Me*_4_). ^13^C{^1^H} NMR (75 MHz, C_6_D_6_): δ 153.3, 136.0, 132.1, 127.1, 124.3, not detected (N*C*_6_H_4_–*C*_6_H_4_N), 107.2 (overlapped, C_5_HMe_4_), 13.6, 11.1 (C_5_H*Me*_4_). Anal. Calcd for C_30_H_34_Cl_4_N_2_Ta_2_ (926.31): C, 38.90; H, 3.70; N, 3.02. Found: C, 38.62; H, 3.79; N, 3.62.

### Crystal Structure Determination of Complexes **1**, **1Br**, **2**, **2Br**, **3Br**, **4**, **7**, **8Br**, and **9**

Crystals were obtained by slow cooling at −20 °C of the corresponding hexane or toluene solution. Single crystals were coated with mineral oil, mounted on Mitegen MicroMounts with the aid of a microscope, and immediately placed in the low-temperature nitrogen stream of the diffractometer. The intensity data sets for **1**, **1Br**, **2**, **2Br, 4**, and **7** were collected at 200 K on a Bruker-Nonius KappaCCD diffractometer equipped with graphite-monochromated Mo Kα radiation (λ = 0.71073 Å) and an Oxford Cryostream 700 unit, while those for **3Br**, **8Br**, and **9** were collected at 200 K on a Bruker D8 Venture diffractometer equipped with multilayer optics for monochromatization and collimator, with Mo Kα radiation (λ = 0.71073 Å) and an Oxford Cryostream 800 unit. Crystallographic data for all complexes are presented in Table S1 in the Supporting Information.

The structures were solved by using the WINGX package^43^ (Olex2 for [Ta(η^5^-C_5_HMe_4_)Br_4_]),^44^ by direct methods (SHELXS)^45^ or intrinsic phasing (SHELXT, in the case of [Ta(η^5^-C_5_HMe_4_)Br_4_], **1Br**, **2Br**, and **11**)^46^ and refined by least-squares against *F*^2^ (SHELXL).^47^ All non-hydrogen atoms were anisotropically refined, while hydrogen atoms were placed at idealized positions and refined using a riding model, except for the bridging hydride atoms in complexes **1, 1Br, 2, 2Br**, and **3Br** that were located in the difference Fourier maps and isotropically refined; after several cycles of refinement, the coordinates and thermal factors were fixed most of the times. Cyclopentadienyl carbon atoms presented some dynamic disorder in some complexes; thus EADP restraints were applied for **1**, **1Br**, **2**, and **2Br**, while SIMU gave better results in the case of compound **4**.

Complex **3Br** crystallized with two deuterated benzene solvent molecules. On the other hand, one toluene molecule could be modeled in the case of **9**, although voids for two more molecules were found using the Platon Squeeze procedure.^48^ By using this program, the contribution of these disordered toluene molecules to the structure factors was removed.

### Computational Details

All calculations were performed using the Gaussian16 program package.^49^ The geometries, energies, and electronic structures of all the investigated species were calculated within the density functional theory (DFT)^50^ framework using the B3LYP functional.^51−53^ A standard double-ξ Lanl2dz^54^ basis set with an f polarization function for tantalum atoms and with a d polarization function for silicon and chlorine atoms was used.^55,56^ For bromine atoms a Lanl2dz basis set with polarization and diffuse functions was employed.^57−61^ The rest of the atoms such as nitrogen, carbon, and hydrogen were treated with a standard 6-31G(d,p) basis set.^62−64^ We also applied a GD3 dispersion correction^65^ as implemented in Gaussian 16. The transition states were characterized by a single imaginary frequency, whose normal mode corresponded to the expected motion. Moreover, an intrinsic reaction coordinate (IRC) analysis was performed for almost all transition states to confirm their nature and the intermediates that were connected by the transition state. Finally, in order to change the reference from the ideal gas standard state of 1 atm (0.041 mol L^–1^) to 1 mol L^–1^ in the condensed state, we applied a correction on Gibbs free energy values of 1.89 kcal mol^–1^.

## Supporting Information Available

The following files are available free of charge. (PDF). (XYZ) The Supporting Information is available free of charge at https://pubs.acs.org/doi/10.1021/acs.inorgchem.1c03152.

General procedures for the synthesis of the bromo derivatives [Ta(η^5^-C_5_HMe_4_)Br_4_] and **1Br**–**11Br**, experimental data for the X-ray diffraction studies on [Ta(η^5^-C_5_HMe_4_)Br_4_], **1**, **1Br**, **2**, **2Br**, **3Br**, **4**, **7**, **8Br**, and **9**, molecular structures of compounds [Ta(η^5^-C_5_HMe_4_)Br_4_], **1Br**, **2Br**, **3Br**, **4**, and **8Br**, NMR spectra for complexes **1**−**11**and **1Br**−**11Br**, X-ray diffraction vs calculated geometrical parameters for complexes **1** and **2** and their analogues with Br, **1Br**–**3Br**, frontier molecular orbitals of complexes **1**–**3** and HOMO–LUMO energy gaps, alternative N=N double-bond-breaking mechanism for the transformation of **1** into **4**, DFT structures of reactants and product **4**, DFT structures of different reaction intermediates of the mechanism shown in Figure 5, and DFT structures of the transition states of the mechanism shown in Figure 5 (PDF)
Cartesian coordinates in a format for convenient visualization (XYZ)

## Supplementary Material

ic1c03152_si_001.pdf

ic1c03152_si_002.xyz

## Accession Codes

CCDC 2114623–2114631 and 2114665 contain the supplementary crystallographic data for this paper. These data can be obtained free of charge via www.ccdc.cam.ac.uk/data_request/cif, or by emailing data_request@ccdc.cam.ac.uk, or by contacting The Cambridge Crystallographic Data Centre, 12 Union Road, Cambridge CB2 1EZ, UK; fax: +44 1223 336033.

## References

[ref1] Tonks I. A. Ti-Catalyzed and -Mediated Oxidative Amination Reactions. Acc. Chem. Res. 2021, 54, 3476–3490. 10.1021/acs.accounts.1c00368. 34420307 PMC9022015

[ref2] Kawakita K.; Parker B. F.; Kakiuchi Y.; Tsurugi H.; Mashima K.; Arnold J.; Tonks I. A. Reactivity of Terminal Imido Complexes of Group 4–6 Metals: Stoichiometric and Catalytic Reactions Involving Cycloaddition with Unsaturated Organic Molecules. Coord. Chem. Rev. 2020, 407, 213118 10.1016/j.ccr.2019.213118. 32863399 PMC7453927

[ref3] Yi X.; Xi C. Copper-Promoted Tandem Reaction of Azobenzenes with Allyl Bromides via N=N Bond Cleavage for the Regioselective Synthesis of Quinolines. Org. Lett. 2015, 17, 5836–5839. 10.1021/acs.orglett.5b03009. 26580318

[ref4] Tollman W. B.. Activation of Small Molecules: Organometallic and Bioinorganic Perspectives; Wiley-VCH: 2006. 10.1002/3527609350.

[ref5] Nishibayashi Y.. Transition Metal-Dinitrogen Complexes: Preparation and Reactivity; Wiley-VCH: 2019. 10.1002/9783527344260.

[ref6] aLockwood M. A.; Fanwick P. E.; Eisenstein O.; Rothwell I. P. Reduction of Azobenzene at a Single Tungsten Metal Center. J. Am. Chem. Soc. 1996, 118, 2762–2763. 10.1021/ja954010p.

[ref7] aMüller T. E.; Hultzsch K. C.; Yus M.; Foubelo F.; Tada M. Hydroamination: Direct Addition of Amines to Alkenes and Alkynes. Chem. Rev. 2008, 108, 3795–3892. 10.1021/cr0306788. 18729420

[ref8] Connelly N. G.; Geiger W. E. Chemical Redox Agents for Organometallic Chemistry. Chem. Rev. 1996, 96, 877–910. 10.1021/cr940053x. 11848774

[ref9] Milsmann C.; Turner Z. R.; Semproni S. P.; Chirik P. J. Azo N=N Bond Cleavage with a Redox-Active Vanadium Compound Involving Metal–Ligand Cooperativity. Angew. Chem., Int. Ed. 2012, 51, 5386–5390. 10.1002/anie.201201085. 22514017

[ref10] Saito T.; Nishiyama H.; Tanahashi H.; Kawakita K.; Tsurugi H.; Mashima K. 1,4-Bis(trimethylsilyl)-1,4-diaza-2,5-cyclohexadienes as Strong Salt-Free Reductants for Generating Low-Valent Early Transition Metals with Electron-Donating Ligands. J. Am. Chem. Soc. 2014, 136, 5161–5170. 10.1021/ja501313s. 24597916

[ref11] Kawakita K.; Kakiuchi Y.; Beaumier E. P.; Tonks I. A.; Tsurugi H.; Mashima K. Synthesis of Pyridylimido Complexes of Tantalum and Niobium by Reductive Cleavage of the N=N Bond of 2,2′-Azopyridine: Precursors for Early–Late Heterobimetallic Complexes. Inorg. Chem. 2019, 58, 15155–15165. 10.1021/acs.inorgchem.9b02043. 31553585 PMC7017918

[ref12] Fryzuk M. D.; Johnson S. A.; Rettig S. J. New mode of coordination for the dinitrogen ligand: a dinuclear tantalum complex with a bridging N_2_ unit that is both side-on and end-on. J. Am. Chem. Soc. 1998, 120, 11024–11025. 10.1021/ja982377z. 11457146

[ref13] Akagi F.; Matsuo T.; Kawaguchi H. Dinitrogen cleavage by a diniobium tetrahydride complex: formation of a nitride and its conversion into imide species. Angew. Chem., Int. Ed. 2007, 46, 8778–8781. 10.1002/anie.200703336. 17924386

[ref14] Ballmann J.; Munha R. F.; Fryzuk M. D. The hydride route to the preparation of dinitrogen complexes. Chem. Commun. 2010, 46, 1013–1025. 10.1039/b922853e. 20126700

[ref15] Shima T.; Hou Z. Dinitrogen Fixation by Transition Metal Hydride Complexes. Top. Organomet. Chem. 2017, 60, 23–44. 10.1007/3418_2016_3.

[ref16] Shima T.; Hu S.; Luo G.; Kang X.; Luo Y.; Hou Z. Dinitrogen Cleavage and Hydrogenation by a Trinuclear Titanium Polyhydride Complex. Science 2013, 340, 1549–1552. 10.1126/science.1238663. 23812710

[ref17] Bellows S. M.; Arnet N. A.; Gurubasavaraj P. M.; Brennessel W. W.; Bill E.; Cundari T. R.; Holland P. L. The Mechanism of N–N Double Bond Cleavage by an Iron(II) Hydride Complex. J. Am. Chem. Soc. 2016, 138, 12112–12123. 10.1021/jacs.6b04654. 27598037 PMC5499983

[ref18] Belmonte P. A.; Schrock R. R.; Day C. S. Binuclear Tantalum Hydride Complexes. J. Am. Chem. Soc. 1982, 104, 3082–3089. 10.1021/ja00375a023.

[ref19] Lee T.-Y.; Messerle L. Utility of hydridotributyltin as both reductant and hydride transfer reagent in organotransition metal chemistry I. A convenient synthesis of the organoditantalum(IV) hydrides (η-C_5_Me_4_R)_2_Ta_2_(μ-H)_2_Cl_4_ (R = Me, Et) from (η-C_5_Me_4_R)TaCl_4_, and probes of the possible reaction pathways. J. Organomet. Chem. 1998, 553, 397–403. 10.1016/S0022-328X(97)00620-7.

[ref20] aSattelberger A. P.; Wilson R. B.; Huffman J. C. Metal-metal Bonded Complexes of the Early Transition Metals. 5. Direct Hydrogenation of a Metal-Metal Multiple Bond. Inorg. Chem. 1982, 21, 4179–4184. 10.1021/ic00142a014.

[ref21] Gómez M.; Gómez-Sal P.; Nicolás M. P.; Royo P. Monopentamethylcyclopentadienyl isocyanide, amine and imido tantalum(V) complexes. X-ray crystal structure of [TaCp*Cl_4_(CN-2,6-Me_2_C_6_H_3_]. J. Organomet. Chem. 1995, 491, 121–125. 10.1016/0022-328X(94)05236-5.

[ref22] Cotton F. A.; Duraj S. A.; Roth W. J. A New Double Bond Metathesis Reaction: Conversion of an Nb=Nb and an N=N Bond into Two Nb=N Bonds. J. Am. Chem. Soc. 1984, 106, 4749–4751. 10.1021/ja00329a018.

[ref23] Canich J. A. M.; Cotton F. A.; Duraj S. A.; Roth W. J. The Preparation of Ta_2_Cl_6_(PhN)_2_(Me_2_S)_2_ by reaction of Ta_2_Cl_6_(Me_2_S)_3_ with PhNNPh: Crystal Structure of the Product. Polyhedron 1986, 5, 895–898. 10.1016/S0277-5387(00)84454-X.

[ref24] Ikeda H.; Nishi K.; Tsurugi H.; Mashima K. Metathesis cleavage of an N=N bond in benzo[c]cinnolines and azobenzenes by triply-bonded ditungsten complexes. Chem. Commun. 2018, 54, 3709–3711. 10.1039/C7CC08570B. 29441373

[ref25] Groom C. R.; Bruno I. J.; Lightfoot M. P.; Ward S. C. The Cambridge Structural Database. Acta Crystallogr., Sect. B: Struct. Sci., Cryst. Eng. Mater. 2016, B72, 171–179. 10.1107/S2052520616003954. PMC4822653 27048719

[ref26] Saito T.; Nishiyama H.; Kawakita K.; Nechayev M.; Kriegel B.; Tsurugi H.; Arnold J.; Mashima K. Reduction of (*t*BuN=)NbCl_3_(py)_2_ in a Salt-Free Manner for Generating Nb(IV) Dinuclear Complexes and Their Reactivity toward Benzo[c]cinnoline. Inorg. Chem. 2015, 54, 6004–6009. 10.1021/acs.inorgchem.5b00812. 26017157

[ref27] Doedens R. J. Structural Studies of Organonitrogen Compounds of the Transition Elements. IV. The Crystal and Molecular Structure of Benzo[c]cinnolinebis(tricarbonyliron), C_12_H_8_N_2_Fe_2_(CO)_6_. Inorg. Chem. 1970, 9, 429–436. 10.1021/ic50085a001.

[ref28] Gonzlez-Maupoey M.; Rodriguez G. M.; Cuenca T. μ-Imido, μ-(η^2^,η^2^-N,N-Hydrazido) and μ-(η^1^-C:η^2^-C,N-Isocyanido) Dinuclear (Fulvalene)zirconium Derivatives. Eur. J. Inorg. Chem. 2002, 2002, 2057–2063. 10.1002/1099-0682(200208)2002:8<2057::AID-EJIC2057>3.0.CO;2-P.

[ref29] Van der Meer H. The Crystal Structure of 9,10-Diazaphenanthrene. Acta Crystallogr., Sect. B: Struct. Crystallogr. Cryst. Chem. 1972, B28, 367–370. 10.1107/S0567740872002420.

[ref30] aCattaneo P.; Persico M. An *ab initio* study of the photochemistry of azobenzene. Phys. Chem. Chem. Phys. 1999, 1, 4739–4743. 10.1039/a905055h.

[ref31] Schulze F. W.; Detrik H. J.; Camenga H. K.; Klinge H. Thermodynamic Properties of the Structural Analogues Benzo[c]cinnoline, *Trans*-azobenzene, and *Cis*-azobenzene. Z. Phys. Chem. (Muenchen, Ger.) 1977, 107, 1 10.1524/zpch.1977.107.1.001.

[ref32] Adamson A. W.; Vogler A.; Kunkely H.; Wachter R. Photocalorimetry. Enthalpies of Photolysis of *trans*-Azobenzene, Ferrioxalate and Cobaltioxalate Ions, Chromium Hexacarbonyl, and Dirhenium Decarbonyl. J. Am. Chem. Soc. 1978, 100, 1298–1300. 10.1021/ja00472a049.

[ref33] Shaver M. P.; Fryzuk M. D. Activation of Molecular Nitrogen: Coordination, Cleavage and Functionalization of N_2_ Mediated By Metal Complexes. Adv. Synth. Catal. 2003, 345, 1061–1076. 10.1002/adsc.200303081.

[ref34] Ohki Y.; Fryzuk M. D. Dinitrogen Activation by Group 4 Metal Complexes. Angew. Chem., Int. Ed. 2007, 46, 3180–3183. 10.1002/anie.200605245. 17366507

[ref35] See for example: Bushuyev O. S.; Brown P.; Maiti A.; Gee R. H.; Peterson G. R.; Weeks B. L.; Hope-Weeks L. J. Ionic Polymers as a New Structural Motif for High-Energy-Density Materials. J. Am. Chem. Soc. 2012, 134, 1422–1425. 10.1021/ja209640k. 22191717

[ref36] Ma X.; Lei M.; Liu Sh. Homolytic or heterolytic dihydrogen splitting with ditantalum/dizirconium dinitrogen complexes? A computational study. Organometallics 2015, 34, 1255–1263. 10.1021/om501316t.

[ref37] Aguado-Ullate S.; Carbó J. J.; González-del Moral O.; Martín A.; Mena M.; Poblet J. M.; Santamaría C. Ammonia Activation by μ_3_-Alkylidyne Fragments Supported on a Titanium Molecular Oxide Model. Inorg. Chem. 2011, 50, 6269–6279. 10.1021/ic2006327. 21619036

[ref38] Carbó J. J.; García-López D.; González-del Moral O.; Martín A.; Mena M.; Santamaría C. Carbon–Nitrogen Bond Construction and Carbon-Oxygen Double Bond Cleavage on a Molecular Titanium Oxonitride: A Combined Experimental and Computational Study. Inorg. Chem. 2015, 54, 9401–9412. 10.1021/acs.inorgchem.5b00943. 26365632

[ref39] Carbó J. J.; García-López D.; Gómez-Pantoja M.; González-Pérez J. I.; Martín A.; Mena M.; Santamaría C. Intermetallic Cooperation in C-H Activation Involving Transient Titanium-Alkylidene Species: A Synthetic and Mechanistic Study. Organometallics 2017, 36, 3076–3083. 10.1021/acs.organomet.7b00416.

[ref40] Carbó J. J.; Gómez-Pantoja M.; Martín A.; Mena M.; Ricart J. M.; Salom-Català A.; Santamaría C. A Bridging bis-Allyl Titanium Complex: Mechanistic Insights into the Electronic Structure and Reactivity. Inorg. Chem. 2019, 58, 12157–12166. 10.1021/acs.inorgchem.9b01505. 31448905

[ref41] Wang B.; Luo G.; Nishiura M.; Hu S.; Shima T.; Luo Y.; Hou Z. Dinitrogen Activation by Dyhydrogen and PNP-Ligated Titanium Complex. J. Am. Chem. Soc. 2017, 139, 1818–1821. 10.1021/jacs.6b13323. 28134522

[ref42] Llinás G. H.; Mena M.; Palacios F.; Royo P.; Serrano R. C_5_Me_5_)SiMe_3_ as a Mild and Effective Reagent for Transfer of the C_5_Me_5_ Ring: An Improved Route to Monopentamethylcyclopentadienyl Trihalides of the Group 4 Elements. J. Organomet. Chem. 1988, 340, 37–40. 10.1016/0022-328X(88)80551-5.

[ref43] Farrugia L. J. WinGX and ORTEP for Windows: an update. J. Appl. Crystallogr. 2012, 45, 849–854. 10.1107/S0021889812029111.

[ref44] Dolomanov O. V.; Bourhis L. J.; Gildea R. J.; Howard J. A. K.; Puschmann H. OLEX2: a complete structure solution, refinement and analysis program. J. Appl. Crystallogr. 2009, 42, 339–341. 10.1107/S0021889808042726.

[ref45] Sheldrick G. M. A short history of SHELX. Acta Crystallogr., Sect. A: Found. Crystallogr. 2008, A64, 112–122. 10.1107/S0108767307043930. 18156677

[ref46] Sheldrick G. M. SHELXT – Integrated space-group and crystal-structure determination. Acta Crystallogr., Sect. A: Found. Adv. 2015, 71, 3–8. 10.1107/S2053273314026370. 25537383 PMC4283466

[ref47] Sheldrick G. M. Crystal structure refinement with SHELXL. Acta Crystallogr., Sect. C: Struct. Chem. 2015, 71, 3–8. 10.1107/S2053229614024218. 25567568 PMC4294323

[ref48] Spek A. L. PLATON SQUEEZE: a tool for the calculation of the disordered solvent contribution to the calculated structure factors. Acta Crystallogr., Sect. C: Struct. Chem. 2015, C71, 9–18. 10.1107/S2053229614024929. 25567569

[ref49] Frisch M. J.; Trucks G. W.; Schlegel H. B.; Scuseria G. E.; Robb M. A.; Cheeseman J. R.; Scalmani G.; Barone V.; Petersson G. A.; Nakatsuji H.; Li X.; Caricato M.; Marenich A. V.; Bloino J.; Janesko B. G.; Gomperts R.; Mennucci B.; Hratchian H. P.; Ortiz J. V.; Izmaylov A. F.; Sonnenberg J. L.; Williams-Young D., Ding F.; Lipparini F.; Egidi F.; Goings J.; Peng B.; Petrone A.; Henderson T.; Ranasinghe D.; Zakrzewski V. G.; Gao J.; Rega N.; Zheng G.; Liang W.; Hada M.; Ehara M.; Toyota K.; Fukuda R.; Hasegawa J.; Ishida M.; Nakajima T.; Honda Y.; Kitao O.; Nakai H.; Vreven T.; Throssell K.; Montgomery J. A. Jr.; Peralta J. E.; Ogliaro F.; Bearpark M. J.; Heyd J. J.; Brothers E. N.; Kudin K. N.; Staroverov V. N.; Keith T. A.; Kobayashi R.; Normand J., Raghavachari K.; Rendell A. P.; Burant J. C.; Iyengar S. S.; Tomasi J.; Cossi M.; Millam J. M.; Klene M.; Adamo C.; Cammi R.; Ochterski J. W.; Martin R. L.; Morokuma K.; Farkas O.; Foresman J. B.; Fox D. J. Gaussian 16, Rev. A.03; Gaussian, Inc.: 2016.

[ref50] Parr R. G.; Yang W. In Density Functional Theory of Atoms and Molecules; Oxford University Press: 1989.

[ref51] Lee C.; Yang W.; Parr R. G. Development of the Colle-Salvetti correlation-energy formula into a functional of the electron density. Phys. Rev. B: Condens. Matter Mater. Phys. 1988, 37, 785–789. 10.1103/PhysRevB.37.785. 9944570

[ref52] Becke A. D. Density-functional thermochemistry. III. The role of exact exchange. J. Chem. Phys. 1993, 98, 5648–5652. 10.1063/1.464913.

[ref53] Stephens P. J.; Devlin F. J.; Chabolowski C. F.; Frisch M. J. Ab Initio Calculation of Vibrational Absorption and Circular Dichroism Spectra Using Density Functional Force Fields. J. Phys. Chem. 1994, 98, 11623–11627. 10.1021/j100096a001.

[ref54] Hay P. J.; Wadt W. R. Ab initio effective core potentials for molecular calculations. Potentials for K to Au including the outermost core orbitals. J. Chem. Phys. 1985, 82, 299–310. 10.1063/1.448975.

[ref55] Höllwarth A.; Böhme M.; Dapprich S.; Ehlers A. W.; Gobbi A.; Jonas V.; Köler K. F.; Stegmann R.; Veldkamp A.; Frenking G. A set of d-polarization functions for pseudo-potential basis sets of the main group elements Al-Bi and f-type polarization functions for Zn, Cd, Hg. Chem. Chem. Phys. Lett. 1993, 208, 237–240. 10.1016/0009-2614(93)89068-S.

[ref56] Ehlers A. W.; Böhme M.; Dapprich S.; Gobbi A.; Höllwarth A.; Jonas V.; Köhler K. F.; Stegmann R.; Veldkamp A.; Frenking G. A set of f-polarization functions for pseudo-potential basis sets of the transition metals Sc-Cu, Y-Ag and La-Au. Chem. Phys. Lett. 1993, 208, 111–114. 10.1016/0009-2614(93)80086-5.

[ref57] Wadt W. R.; Hay P. J. Ab initio effective core potentials for molecular calculations. Potentials for main group elements Na to Bi. J. Chem. Phys. 1985, 82, 284–298. 10.1063/1.448800.

[ref58] Feller D. The Role of Databases in Support of Computational Chemistry Calculations. J. Comput. Chem. 1996, 17, 1571–1586. 10.1002/(SICI)1096-987X(199610)17:13<1571::AID-JCC9>3.0.CO;2-P.

[ref59] Check C. E.; Faust T. O.; Bailey J. M.; Brian J. W.; Gilbert T. M.; Sunderlin L. S. Addition of Polarization and Diffuse Functions to the LANL2DZ Basis Set for p-Block Elements. J. Phys. Chem. A 2001, 105, 8111–8116. 10.1021/jp011945l.

[ref60] Schuchardt K. L.; Didier B. T.; Elsethagen T.; Lisong S.; Gurumoorthi V.; Chase J.; Li J.; Windus T. L. Basis Set Exchange: A Community Database for Computational Sciences. J. Chem. Inf. Model. 2007, 47, 1045–1052. 10.1021/ci600510j. 17428029

[ref61] Pritchard B. P.; Altarawy D.; Didier B.; Gibson T. D.; Windus T. L. New Basis Set Exchange: An open, Up-to-Date Resource for the Molecular Sciences Community. J. Chem. Inf. Model. 2019, 59, 4814–4820. 10.1021/acs.jcim.9b00725. 31600445

[ref62] Hehre W. J.; Ditchfield R.; Pople J. A. Self—Consistent Molecular Orbital Methods. XII. Further Extensions of Gaussian—Type Basis Sets for Use in Molecular Orbital Studies of Organic Molecules. J. Chem. Phys. 1972, 56, 2257–2261. 10.1063/1.1677527.

[ref63] Hariharan P. C.; Pople J. A. The influence of polarization functions on molecular orbital hydrogenation energies. Theor. Chim. Acta 1973, 28, 213–222. 10.1007/BF00533485.

[ref64] Francl M. M.; Pietro W. J.; Hehre W. J.; Binkley J. S.; Gordon M. S.; DeFrees D. J.; Pople J. A. Self-consistent molecular orbital methods. XXIII. A polarization-type basis set for second-row elements. J. Chem. Phys. 1982, 77, 3654–3665. 10.1063/1.444267.

[ref65] Grimme S.; Antony J.; Ehrlich S.; Krieg H. A consistent and accurate ab initio parametrization of density functional dispersion correction (DFT-D) for the 94 elements H-Pu. J. Chem. Phys. 2010, 132, 154104 10.1063/1.3382344. 20423165

