# Supplementary material for: N=N Bond Cleavage by Tantalum Hydride Complexes: Mechanistic Insights and Reactivity
Source: Inorg Chem. 2021 Dec 10;61(1):474–85. doi: 10.1021/acs.inorgchem.1c03152 (PMC8753601; doi:10.1021/acs.inorgchem.1c03152)
Supplement: Supplementary file 1 — ic1c03152_si_001.pdf [file ic1c03152_si_001.pdf]

Supporting Information for the Paper Entitled

N=N Bond Cleavage by a Tantalum Hydride Complex: Mechanistic Insights and Reactivity

Elena Álvarez-Ruiz,† Jorge J. Carbó,‡ Manuel Gómez,† Cristina Hernández-Prieto,† Alberto Hernán-Gómez,† Avelino Martín,† Miguel Mena,† Josep M. Ricart,‡ Antoni Salom-Català‡ and Cristina Santamaría*†.*

†Departamento de Química Orgánica y Química Inorgánica. Instituto de Investigación Química "Andrés M. del Río" (IQAR), Universidad de Alcalá, Campus Universitario, E-28805 Alcalá de Henares, Madrid, Spain.

‡Departament de Química Física i Inorgànica, Universitat Rovira i Virgili, Campus Sescelades, C/ Marcel.lí Domingo, s/n, 43007 Tarragona (Spain)

Table of Contents

Experimental Section

- General procedures for the synthesis of the bromo derivatives [Ta(η^5-C_5HMe_4)Br_4] and **1Br–11Br**………S3-S7

Crystallographic Data

- **Table S1**: Experimental data for the X-ray diffraction studies on [Ta(η^5-C_5HMe_4)Br_4], **1**, **1Br, 2, 2Br, 3Br**, **4**, **7**, **8Br**, and **9** ………………..……………………………:S8-S9
- **Figure S1:** Molecular structure of compound [Ta(η^5-C_5HMe_4)Br_4] with selected bond distances and angles…………………………………………………………………..S10
- **Figure S2:** Molecular structure of compound **1Br**…………………………………..S10
- **Figure S3:** Molecular structure of compound **2Br**.…………………………………...S11
- **Figure S4:** Molecular structure of compound **3Br..**…………………………………...S11
- **Figure S5:** Molecular structure of compound **4** with selected bond distances and angles ………………………………………………………………………………….……...S12
- **Figure S6:** Molecular structure of compound **8Br** ………………..………..……..S12

NMR Spectroscopy

- NMR Spectra for complexes **1-11Br (**including bromo derivatives**)** (**Figure S7-S60**)………………………………………………………………………S13-S39

Theoretical Studies

- **Table S2:** X-Ray diffraction vs Calculated geometrical parameters for complex **1** and **2** and their analogous with Br, **1Br, 2Br** and **3Br**…………………………………..S40
- **Figure S61.** Frontier Molecular Orbitals of complexes **1**, **2** and **3**; and HOMO-LUMO energy gaps…………………………………………………………………………S41
- **Figure S62.** Alternative N=N double bond breaking mechanism for the transformation of **1** into **4**………………………………………………………………………..S42
- **Figure S63.** DFT structures of reactants and product **4**………………………………S42
- **Figure S64.** DFT structures of different reaction intermediates of the mechanism shown in Figure 5……………………………………………………………………….S43

- **Figure S65.** DFT structures of the transition states of the mechanism shown in Figure 5……………………………………………………………………………………S43

Experimental Section

Synthesis of [Ta(η^5-C_5HMe_4)Br_4]. To a toluene (20 ml) suspension of $TaBr_5$ (2.00 g, 3.44 mmol) placed into a Carius tube fitted with a Young's valve was added dropwise $C_5HMe_4SiMe_3$ (670 mg, 3.44 mmol) solved in 10 ml of toluene. The mixture was stirred at 100 °C overnight and then evaporated to dryness. The residue was washed with two portions of hexane (40 mL) and dried in vacuo to give [Ta(η^5-C_5HMe_4)Br_4] as a red solid (Yield: 1.97 g, 92 %). IR (KBr, cm^{-1}): $\bar{\nu}$ = 3081 (m, CH arom.), 2994 (w, CH aliph.), 2965 (w, CH aliph.), 2915 (w, CH aliph.), 1767 (w, CC), 1568 (m, CC), 1498 (m, CC), 1472 (m, CC), 1449 (m, CC), 1423 (m, CC), 1378 (s), 1016 (s), 883 (s), 601 (w), 429 (w) ^{1}H NMR (300 MHz, C_6D_6): δ = 5.64 (s, 1H, C_5*H*Me_4), 2.36, 2.12 (s, 6H, C_5H*Me*$_4$). $^{13}C\{^1H\}$ NMR (75 MHz, C_6D_6): δ= 133.8, 132.9 (*C*-Me arom.), 121.5 (*CH*$_{arom}$), 17.7, 14.9 (C_5H*Me*$_4$). Elemental analysis (%) calcd. for $C_9H_{13}Br_4Ta$ (621.76): C, 17.38; H, 2.11; found: C, 17.30; H, 2.18.

General procedure for the synthesis of [($TaCp^RBr_2$)$_2$(μ-H)$_2$] (Cp^R = η^5-C_5Me_5, 1Br; Cp^R = η^5-$C_5H_4SiMe_3$, 2Br; Cp^R = η^5-C_5HMe_4 3Br). A toluene (40-45 mL) solution of [$TaCp^RX_4$] (Cp^R = η^5-C_5Me_5, η^5-$C_5H_4SiMe_3$, η^5-C_5HMe_4) and SiH_3Ph was placed into a Carius tube fitted with a Young's valve, and under rigorously anhydrous conditions, the reaction mixture was stirred and heated. The resulting dark blue/green solutions were then filtered, and the solvent was removed under reduced pressure to afford microcrystalline dark blue/green solids. Suitable crystals for single crystal x-ray diffraction were obtained by cooling of the reaction mixture to room temperature.

Synthesis of [{Ta(η^5-C_5Me_5)Br_2}$_2$(μ-H)$_2$] (1Br). The thermal treatment at 90 °C for 48 hours of SiH_3Ph (0.340 g, 3.14 mmol) with [Ta(η^5-C_5Me_5)Br_4] (1.000 g, 1.57 mmol) in toluene afforded the complex **1Br** as a microcrystalline dark green solid (Yield: 0.71 g, 95 %). IR (KBr, cm^{-1}): $\bar{\nu}$ = 2985 (m, CH aliph), 2962 (m, CH aliph), 2907 (s, CH aliph), 1590 (s, Ta-H), 1483 (s, CC), 1426 (m, CC), 1380 (s, CC), 1023 (s), 804 (w), 733 (m), 696 (m), 421 (w). ^{1}H NMR (300 MHz, C_6D_6): δ= 11.24 (s, 2H, Ta-*H*), 2.10 (s, 30H, C_5*Me*$_5$). $^{13}C\{^1H\}$ NMR (75 MHz, C_6D_6): δ= 122.6 (*C*$_5$Me$_5$), 14.6 (C_5*Me*$_5$). Elemental analysis (%) calcd. for $C_{20}Br_4H_{32}Ta_2$ (953.98): C, 25.18; H, 3.38; found: C, 24.98; H, 3.30.

[{Ta(η^5-$C_5H_4SiMe_3$)Br_2}$_2$(μ-H)$_2$] (2Br). The thermal treatment at 70 °C for 48 hours of SiH_3Ph (0.237 g, 2.194 mmol) with [Ta(η^5-$C_5H_4SiMe_3$)Br_4] (0.700 g, 1.097 mmol) in toluene afforded the complex **2Br** as a microcrystalline dark green solid (Yield: 0.430 g, 82 %). IR

(KBr, cm^{-1}): $\bar{\nu}$ = 3102 (w, CH ar) 2954 (m, CH aliph), 2896 (w, CH aliph), 1509 (w, Ta-H), 1397 (m, CC), 1250 (s, $SiMe_3$), 1169 (m), 904 (m), 838 (vs, $SiMe_3$), 760 (m, CH ar). ^{1}H-NMR (300 MHz, C_6D_6): δ = 10.79 (s, 2H, Ta-*H*), 0.24 (s, 18H, $C_5H_4Si\mathit{Me}_3$), not observed ($C_5\mathit{H}_4SiMe_3$). ^{13}C{^{1}H}-NMR (75 MHz, C_6D_6): δ= 0.32 ($C_5H_4Si\mathit{Me}_3$), not observed ($\mathit{C}_5H_4SiMe_3$). Elemental analysis (%) calcd. for $C_{16}Br_4H_{28}Si_2Ta_2$ (958.08): C, 20.06; H, 2.95; found: C, 19.80; H, 2.91.

Synthesis of [{Ta(η^5-C_5HMe_4)Br_2}$_2$(μ-H)$_2$] (3Br). The thermal treatment at 100 ºC for 24 hours of SiH_3Ph (0.348 g, 3.217 mmol) with [Ta(η^5-C_5HMe_4)Br_4] (1.000 g, 1.608 mmol) in toluene afforded the complex **3Br** (0.700 g, 94 %) as a microcrystalline dark solid. IR (KBr, cm^{-1}): $\bar{\nu}$ = 2984 (w, CH aliph.), 2961 (w, CH aliph.), 2915 (m, CH aliph.), 2855 (w, CH aliph.), 1513 (s, Ta-H), 1480 (s, CC), 1380 (s, CC), 1319 (w, CC), 1149 (w), 1021 (m), 855 (m), 802 (m). ^{1}H NMR (300 MHz, C_6D_6): δ = 10.97 (s, 2H, Ta-*H*), 7.42 (s, 2H, $C_5\mathit{H}Me_4$), 2.16 (bs, 6H, $C_5H\mathit{Me}_4$), 1.79 (s, 6H, $C_5H\mathit{Me}_4$). ^{13}C{^{1}H} NMR (75 MHz, C_6D_6): δ= 126.3 (*C*H), not detected, 98.7 (C_5HMe_4), 14.5 (bs, $C_5H\mathit{Me}_4$), 12.3 ($C_5H\mathit{Me}_4$). Elemental analysis (%) calcd. for $C_{18}H_{28}Br_4Ta_2$ (925.93): C, 23.35; H, 3.05; found: C, 23.40; H, 3.23.

General procedure for the synthesis of [TaCp^{R}Br$_2$(NPh)] (Cp^R = η^5-C_5Me_5 4Br; η^5-$C_5H_4SiMe_3$ 5Br; η^5-C_5HMe_4 6Br). Ph_2N_2 was added to a toluene solution (30-40 mL) of [(TaCp^{R}Br$_2$)$_2$(μ-H)$_2$] (Cp^R = η^5-C_5Me_5, η^5-$C_5H_4SiMe_3$; η^5-C_5HMe_4) placed into a Carius tube (100 mL) with a Young's valve. The argon pressure was reduced, and the reaction mixture was stirred, was heated for 24-48 hours, and then filtered. The solvent was removed under reduced pressure to afford microcrystalline orange solids (**4Br**, **6Br**) or a dark orange oil (**5Br**).

Synthesis of [Ta(η^5-C_5Me_5)Br_2(NPh)] (4Br). The thermal treatment at 50 ºC for 24 hours of Ph_2N_2 (0.076 g, 0.419 mmol) with [{Ta(η^5-C_5Me_5)Br_2}$_2$(μ-H)$_2$] (**1Br**) (0.400 g, 0.419 mmol) in toluene rendered the compound **4Br** as an orange solid (Yield: 0.404 g, 85 %). **METHOD B:** A 100 mL Schlenk vessel was charged in the glovebox with Ph_2N_2 (0.043 g, 0.236 mmol), [Ta(η^5-C_5Me_5)Br_4] (0.300 g, 0.472 mmol), Mg (0.011 g, 0.472 mmol), and thf (30-40 mL). After stirring for 24 hours at room temperature, the reaction mixture was dried in vacuum, the product was extracted with toluene and filtered through a medium porosity glass frit, and the solvent was then removed in vacuum to yield **4Br** (0.193 g, 72 %) as an orange solid. IR (KBr, cm^{-1}): $\bar{\nu}$ = 3066 (w, CH arom), 2960 (w, CH aliph), 2914 (w, CH aliph), 1584 (m, CC), 1481 (s, CC), 1349 (s, =NR), 1067 (m), 982 (m), 768 (s), 693 (m). ^{1}H NMR (300 MHz,

C_6D_6): δ = 7.09 (t, 2H, *J* = 9 Hz, H_p NPh), 6.88 (d, 2H, *J* = 9 Hz, H_o NPh), 6.73 (t, 1H, *J* = 9 Hz, H_m NPh), 1.88 (s, 15H, C_5*Me*$_5$). $^{13}C\{^1H\}$-NMR (75 MHz, C_6D_6): δ = not detected (C_{ipso}), 128.3, 126.2, 124.5 (N*Ph*), 121.8 (*C*$_5$Me$_5$), 11.9 (C_5*Me*$_5$). Elemental analysis (%) calcd. for $C_{16}Br_2H_{20}NTa$ (567.09): C, 33.88; H, 3.55; N, 2.47; found: C, 33.85; H, 3.61; N, 3.15.

Synthesis of [Ta(η^5-$C_5H_4SiMe_3$)Br_2(NPh)] (5Br). The thermal treatment at 60 ºC for 24 hours of Ph_2N_2 (0.095 g, 0.522 mol) with [{Ta(η^5-$C_4H_4SiMe_3$)Br_2}$_2$(μ-H)$_2$] (**2Br**) (0.500 g, 0.522 mmol) in toluene rendered the complex **5Br** as dark orange oil (Yield: 0.520 g, 87 %) IR (KBr, cm^{-1}): $\bar{\nu}$ = 3096 (w, CH arom), 2954 (m, CH aliph), 2896 (m, CH aliph), 1562 (m, CC), 1482 (s, CC), 1351 (s, =NR), 1251 (s, $SiMe_3$), 1069 (m), 841 (vs, $SiMe_3$), 759 (s), 689 (m). ^1H NMR (300 MHz, C_6D_6): δ = 7.08, 6.89, 6.73 (NPh), 6.23, 5.91 (spin system AA’BB’, 4H, C_5*H*$_4$SiMe$_3$), 0.12 (s, 9H, C_5H_4Si*Me*$_3$). $^{13}C\{^1H\}$ NMR (75 MHz, C_6D_6): δ = 156.0 (Cipso NPh), 128.1, 125.9, 125.5 (NPh), 120.3, 114.0 (*C*$_5$H$_4$SiMe$_3$), -0.1 (sC_5H_4Si*Me*$_3$). Elemental analysis (%) calcd. for $C_{14}H_{18}Br_2NSiTa$ (569.14): C, 29.54; H, 3.19; N, 2.46; found: C, 29.02; H, 3.47; N, 2.73.

Synthesis of [Ta(η^5-C_5HMe_4)Br_2(NPh)] (6Br). The thermal treatment at 70 ºC for 24 hours of Ph_2N_2 (0.079 g, 0.432 mmol) with [{Ta(η^5-C_5HMe_4)Br_2}$_2$(μ-H)$_2$] (**3Br**) (0.400 g, 0.432 mmol) in toluene rendered the compound **6Br** as an orange solid (Yield. 0.340 g, 71 %). **METHOD B:** A 100 mL Schlenk vessel was charged in the glovebox with Ph_2N_2 (0.059 g, 0.322 mmol), [Ta(η^5-C_5HMe_4)Br_4] (0.400 g, 0.643 mmol), Mg (0.016g, 0.643 mmol), and thf (30-40 mL). After stirring for 24 hours at room temperature, the reaction mixture was dried in vacuum, the product was extracted with toluene and filtered through a medium porosity glass frit, and the solvent was then removed in vacuum to yield **6Br** as an orange solid (Yield: 0.306 g, 86 %). IR (KBr, cm^{-1}): $\bar{\nu}$ = 3073 (w, CH arom), 2960 (w, CH aliph), 2914 (w, CH aliph.), 1601 (w, CC), 1581 (m, CC), 1494 (s, CC), 1481 (m, CC), 1382 (m, CC), 1353 (s, =NR), 1068 (m), 764 (s), 693 (s). ^1H NMR (300 MHz, C_6D_6): δ = 7.20- 6.60 (m, 5H, Ph), 5.36 (s, 1H, C_5*H*Me$_4$), 1.92, 1.83 (s, 6H, C_5H*Me*$_4$). $^{13}C\{^1H\}$ NMR (125 MHz, C_6D_6): δ = 126.0, 125.0, 124.8, 122.1 (s, Ph), 108.1 (overlapped, *C*$_5$HMe$_4$), 14.0, 11.9 (C_5H*Me*$_4$). Elemental analysis (%) calcd. for $C_{15}H_{18}Br_2NTa$ (553.06): C, 32.57; H, 3.28; N 2.53; found: C, 32.87; H, 3.47; N, 3.17.

Synthesis of [{Ta(η^5-C_5Me_5)Br_2}$_2$(μ-NC_6H_4-C_6H_4N)] (7Br). A 100 mL Schlenk vessel was charged in the glovebox with benzo[c]cinnoline (0.038 g, 0.210 mmol), [{Ta(η^5-C_5Me_5)Br_2}$_2$(μ-H)$_2$] (**1Br**) (0.200 g, 0.210 mmol), and toluene (30-40 mL). After stirring for

24 hours at room temperature, the reaction mixture was filtered through a medium porosity glass frit, and the solvent was then removed in vacuum to yield **7Br** (Yield: 0.216 g, 91 %) as an orange solid. **METHOD B:** A 100 mL Schlenk vessel was charged in the glovebox with benzo[c]cinnoline (0.071 g, 0.393 mmol), [Ta(η^5-C_5Me_5)Br_4] (0.500 g, 0.786 mmol), Mg (0.019 g, 0.786 mmol), and thf (30-40 mL). After stirring for 24 hours at room temperature, the reaction mixture was dried in vacuum, the product was extracted with toluene and filtered through a medium porosity glass frit, and the solvent was then removed in vacuum to yield **7Br** (0.334 g, 75 %) as an orange solid. IR (KBr, cm^{-1}): $\bar{\nu}$ = 3059 (w, CH arom), 2960 (w, CH aliph), 2914 (w, CH aliph), 1586 (w, CC), 1461 (m, CC), 1418 (m, CC), 1334 (s, =NR), 1114 (w), 1024 (w), 974 (w), 759 (s). ^{1}H NMR (500 MHz, C_6D_6): δ = 7.55-6.80 (m, 8H, NC_6H_4-C_6H_4N), 1.92 (s, 30H, C_5Me_5). ^{13}C{^{1}H} NMR (125 MHz, C_6D_6): δ= 152.2, 135.2, 132.9, 128.7, 127.1, 124.4 (NC_6H_4-C_6H_4N), 121.9 (C_5Me_5), 12.2 (C_5Me_5). Elemental analysis (%) calcd. for $C_{32}Br_4H_{38}N_2Ta_2$ (1132.17): C, 33.95; H, 3.38; N 2.47; found C, 34.31; H, 3.42; N, 2.84.

Synthesis of [{Ta(η^5-$C_5H_4SiMe_3$)Br_2}$_2${μ-(η^2,η^2-$NC_6H_4C_6H_4N$)}] (8Br). A 100 mL Schlenk vessel was charged in the glovebox with benzo[c]cinnoline (0.094 g, 0.522 mmol), [{Ta(η^5-$C_5H_4SiMe_3$)Br_2}$_2$(μ-H)$_2$] (**2Br**) (0.500 g, 0.522 mmol), and toluene (30-40 mL). After stirring for 24 hours at room temperature, the reaction mixture was filtered through a medium porosity glass frit, and the solvent was then removed in vacuum to yield **8Br** (Yield: 0.540 g, 91 %) as a violet microcrystalline solid. IR (KBr, cm^{-1}): $\bar{\nu}$ = 3072 (w, CH arom), 2952 (s, CH aliph), 2894 (m, CH aliph), 1601 (w), 1481 (m, CC), 1436 (m, CC), 1252 (s, $SiMe_3$), 1168 (m), 902 (m), 840 (vs, $SiMe_3$), 756 (s). ^{1}H NMR (300 MHz, C_6D_6): δ = 7.77-6.82 (m, 8H, NC_6H_4-C_6H_4N), 7.63-5.61 (m, 8 H, $C_5H_4SiMe_3$), 0.25 (s, 18 H, $C_5H_4SiMe_3$). ^{13}C{^{1}H} NMR (75 MHz, C_6D_6): δ = 153.3, 125.9, 125.3, 122.0, 121.1 (NC_6H_4-C_6H_4N), 125.0, 119.2, 116.2, 110.6 ($C_5H_4SiMe_3$), 0.7 ($C_5H_4SiMe_3$). Elemental analysis (%) calcd. for $C_{28}H_{34}N_2Br_4Si_2Ta_2$ (1136.2657): C, 29.60; H, 3.01; N, 2.46; found: C, 29.97; H, 3.56; N, 2.79.

Synthesis of [{Ta(η^5-C_5HMe_4)Br_2}$_2${μ-(η^2,η^2-NC_6H_4-C_6H_4N)}] (9Br). A 100 mL Schlenk vessel was charged in the glovebox with benzo[c]cinnoline (0.058 g, 0.324 mmol), [{Ta(η^5-C_5HMe_4)Br_2}$_2$(μ-H)$_2$] (**3Br**) (0.300 g, 0.324 mmol), and toluene (30-40 mL). After stirring for 24 hours at room temperature, the reaction mixture was filtered through a medium porosity glass frit, and the solvent was then removed in vacuum to yield **9Br** as a microcrystalline purple solid (Yield: 0.308 g, 86%). **METHOD B:** A 100 mL Schlenk vessel

was charged in the glovebox with benzo[c]cinnoline (0.058 g, 0.322 mmol), [Ta(η^5-C_5HMe_4)Br_4] (0.400 g, 0.643 mmol), Mg (0.016 g, 0.643 mmol), and thf (30-40 mL). After stirring for 24 hours at room temperature, the reaction mixture was dried in vacuum, the product was extracted with toluene and filtered through a medium porosity glass frit, and the solvent was then removed in vacuum to yield **9Br** as a purple solid (Yield: 0.266 g, 75%). IR (KBr, cm^{-1}): $\bar{\nu}$ = 3081 (w, CH arom), 3058 (w, CH aliph), 2996 (w, CH aliph), 2963 (w, CH aliph), 2911 (w, CH aliph.), 1601 (m, CC), 1481 (s, CC), 1455 (s, CC), 1433 (m, CC), 1377 (m, Ta-N), 1254 (s), 1102 (m), 976 (w), 755 (s), 462 (w). ^1H NMR (500 MHz, C_6D_6): δ = 8.02 (s, 2H, $C_5\mathit{H}Me_4$), 8.10-6.80 (m, 8H, $NC_6\mathit{H}_4$-$C_6\mathit{H}_4N$), 2.69, 2.11, 1.85, 1.65 (s, 6H, $C_5H\mathit{Me}_4$). $^{13}C\{^1H\}$ NMR (125 MHz, C_6D_6): δ= 154.1, 132.4, 129.3, 125.6, 122.5, 121.8 ($N\mathit{C}_6H_4$-C_6H_4N), 101.0 (overlapped, C_5HMe_4), 17.1, 14.4, 13.9, 13.1 ($C_5H\mathit{Me}_4$). Elemental analysis (%) calcd. for $C_{30}H_{34}Br_4N_2Ta_2$ (1104.11): C, 32.63; H, 3.10; N 2.54; found: C, 33.02; H, 3.22; N, 3.07.

Synthesis of [{Ta(η^5-$C_5H_4SiMe_3$)Br_2}$_2$(μ-NC_6H_4-C_6H_4N)] (10Br). A toluene solution (20 mL) of **8Br** (0.400 g, 0.352 mmol) were placed in a 25-mL Carious tube and then sealed under vacuum by flame. The reaction mixture was heated in an autoclave at 200ºC for four days, and then was allowed to cool to room temperature affording orange solutions. The Carious tube was opened in a glovebox, the solution was decanted, and the solvent was then removed in vacuum to yield **10Br** (Yield: 0.360, 92%). IR (KBr, cm^{-1}): $\bar{\nu}$ = 3093 (w, CH arom), 2955 (m, CH aliph), 2897 (m, CH aliph), 1572 (w), 1481 (m, CC), 1435 (m, CC), 1344 (m, =NR), 1250 (s, $SiMe_3$), 905 (m), 841 (vs, $SiMe_3$), 758 (s).. ^1H NMR (300 MHz, C_6D_6): δ = 7.36-6.75 (m, 8H, $NC_6\mathit{H}_4$-$C_6\mathit{H}_4N$), 6.26-5.90 (spin system AA'BB', m, 8 H, $C_5\mathit{H}_4SiMe_3$), 0.23 (s, 18 H, $C_5H_4Si\mathit{Me}_3$). $^{13}C\{^1H\}$ NMR (75 MHz, C_6D_6): δ = 154.5, 136.3, 131.8, 127.4, 127.1, 125.0 ($N\mathit{C}_6H_4$-C_6H_4N), 121.5, 112.6 ($\mathit{C}_5H_4SiMe_3$), -0.1 ($C_5\mathit{H}_4Si\mathit{Me}_3$). Elemental analysis (%) calcd. for $C_{28}H_{34}Br_4N_2Si_2Ta_2$ (1136.27): C, 29.60; H, 3.01; N, 2.46 found: C, 29.90; H, 3.19; N, 2.86.

Synthesis of [{Ta(η^5-C_5HMe_4)Br_2}$_2$(μ-NC_6H_4-C_6H_4N)] (11Br). Benzo[c]cinnoline (0.058 g, 0.324 mmol) was added to a toluene solution (30-40 mL) of [{Ta(η^5-C_5HMe_4)Br_2}$_2$(μ-H)$_2$] (**3Br**) (0.300 g, 0.324 mmol) placed into a Carius tube (100 mL) with a Young's valve. The argon pressure was reduced, and the reaction mixture was heated to 90 ºC for 24 hours, and then filtered. The resulting orange solid **11Br** (0.297 g, 83%) was obtained after drying under vacuum. IR (KBr, cm^{-1}): $\bar{\nu}$ = 3056 (w, CH arom), 2976 (w, CH aliph), 2960 (w, CH aliph), 2915 (w, CH aliph.), 1584 (w, CC), 1486 (s, CC), 1448 (s, CC), 1423 (m, CC), 1339 (s,

=NR), 1112 (w), 1105 (w), 761 (s). ^{1}H NMR (500 MHz, C_6D_6): δ = 7.50-6.80 (m, 8H, $NC_6\mathit{H}_4$-$C_6\mathit{H}_4N$), 5.55 (s, $C_5\mathit{H}Me_4$), 2.00, 1.83 (s, 12H, $C_5H\mathit{Me}_4$). ^{13}C{^{1}H} NMR (125 MHz, C_6D_6): δ= not detected (C_1), 135.6, 132.5, 127.3, 124.5, not detected ($N\mathit{C}_6H_4$-C_6H_4N), 107.7 (overlapped, C_5HMe_4), 14.2, 11.7 ($C_5H\mathit{Me}_4$). Elemental analysis (%) calcd. for $C_{30}H_{34}Br_4N_2Ta_2$ (1104.12): C, 32.63; H, 3.10; N 2.54; found: C, 32.67; H, 3.28; N, 3.15.

(1) Hidalgo Llinás, G; Mena, M.; Palacios, F.; Royo, P.; Serrano, R. (C_5Me_5)$SiMe_3$ as a Mild and Effective Reagent for Transfer of the C_5Me_5 Ring: An Improved Route to Monopentamethylcyclopentadienyl Trihalides of the Group 4 Elements. *J. Organomet. Chem*. **1998**, *340*, 37-40.

Crystallographic Data

Table S1. Experimental data for the X-ray diffraction studies on [Ta(η^5-C_5HMe_4)Br_4], **1**, **1Br, 2, 2Br, 3Br**, **4**, **7**, **8Br**, and **9**.

	[Ta(η^5-C_5HMe_4)Br_4]	**1**	**1Br**	**2**	**2Br**
Formula	$C_9H_{13}Br_4Ta$	$C_{20}H_{32}Cl_4Ta_2$	$C_{20}H_{32}Br_4Ta_2$	$C_{16}H_{28}Cl_4Si_2Ta_2$	$C_{16}H_{28}Br_4Si_2Ta_2$
M	621.78	776.15	953.99	780.26	958.1
T [K]	200(2)	200(2)	200(2)	200(2)	200(2)
λ[Å]	0.71073	0.71073	0.71073	0.71073	0.71073
Crystal system	triclinic	orthorhombic	orthorhombic	triclinic	triclinic
Space group	P-1	Fdd2	Fdd2	P-1	P-1
a [Å]; α [º]	9.1291(5); 115.417(2)	14.523(1)	14.631(1)	7.6832(7); 110.887(5)	7.91.7(7); 112.33(1)
b [Å]; β [º]	12.3854(6); 95.581(2)	37.796(1)	37.977(3)	11.9799(4);97.956(7)	12.143(2);99.33(1)
c [Å]; γ [º]	14.2864(8); 104.780(2)	8.868(1)	9.1002(5)	14.664(1);93.191(5)	14.610(4);92.669(9)
V [Å^3]	1370.60(13)	4867.6(6)	5056.4(7)	1240.8(2)	1272.5(4)
Z	4	8	8	2	2
ρ_{calcd} [g cm^{-3}]	3.013	2.118	2.506	2.088	2.501
μ [mm^{-1}]	19.644	9.427	14.975	9.336	14.967
F(000)	1120	2928	3504	732	876
Crystal size [mm^3]	0.47 x0.15 x 0.10	0.10 x 0.10 x 0.10	0.10 × 0.10 × 0. 05	0.47 x 0.30 x 0.14	0.27 x 0.21 x 0.04
θ range [deg]		3.00 – 25.03	2.69 – 25.03	2.69 – 25.03	2.63 – 25.03
Index ranges	–11 to 11,<br>–16 to 16,<br>–18 to 18	–17 to 17,<br>–44 to 44,<br>–10 to 10	–17 to 17,<br>–44 to 44,<br>-10 to 10	–9 to 9,<br>–15 to 14,<br>–19 to 19	–10 to 10,<br>–15 to 15,<br>–18 to 18
Reflections collected	33179	16361	17370	24018	24339
Unique data	6132(R_{int} = 0.076)	2141(R_{int} = 0.086)	2236 (R_{int} = 0.159)	4358(R_{int} = 0.142)	4492(R_{int} = 0.155)
Reflections [I>2σ(I)]	4997	1799	**1815**	2715	2369
Goodness-of-fit on F^2	1.052	1.089	1.096	1.017	1.126
Final R indices [I>2σ(I)]	R1 = 0.034<br>wR2 = 0.049	R1 = 0.038<br>wR2 = 0.073	R1 = 0.062<br>wR2 = 0.142	R1 = 0.048<br>wR2 = 0.105	R1 = 0.064<br>wR2 = 0.122
R indices (all data)	R1 = 0.073<br>wR2 = 0.079	R1 = 0.055<br>wR2 = 0.081	R1 = 0.091<br>wR2 = 0.167	R1 = 0.111<br>wR2 = 0.125	R1 = 0.183<br>wR2 = 0.180
Largest diff. peak/hole [e·Å^{-3}]	1.340/-1.239	2.753/-1.857	2.71/–2.316	2.612/ -2.258	2.509 / -2.436

[a] $R1 = \Sigma||F_0|-|F_c||/[\Sigma|F_0|]$ $wR2 = \{[\Sigma w(F_0^2 - F_c^2)^2] / [\Sigma w(F_0^2)^2]\}^{1/2}$

Table S1(cont.). Experimental data for the X-ray diffraction studies on [Ta(η^5-C_5HMe_4)Br_4], **1**, **1Br, 2**, **2Br, 3-Br**, **4**, **7**, **8Br**, and **9.**

	**3Br**	**4**	**7**	**8Br**	**9**
Formula	$C_{30}H_{40}Br_4Ta_2$	$C_{16}H_{20}Cl_2NTa$	$C_{32}H_{38}Cl_4N_2Ta_2$	$C_{28}H_{34}Br_4N_2Si_2Ta_2$	$C_{37}H_{42}Cl_4N_2Ta_2$
M	1082.16	478.18	954.34	1136.29	1018.42
T [K]	200(2)	200(2)	200(2)	200(2)	200(2)
λ[Å]	0.71073	0.71073	0.71073	0.71073	0.71073
Crystal system	tetragonal	orthorhombic	monoclinic	monoclinic	orthorhombic
Space group	$P4_122$	Pbca	$P2_1/c$	C2/c	Pbca
a [Å]; α [º]	9.5580(4)	13.136(1)	15.562(2)	32.504(2)	14.0319(4)
b [Å]; β [º]	9.5580(4)	14.313(1)	13.649(1); 110.39(1)	15.1481(8);98.185(2)	16.0226(5)
c [Å]; γ [º]	34.417(2)	18.167(1)	16.351(1)	13.5051(6)	33.757(1)
V [$Å^3$]	3326.9(3)	3415.7(3)	3255.3(5)	6581.8(6)	7589.6(4)
Z	4	8	4	8	8
ρ_{calcd} [g cm^{-3}]	2.161	1.86	1.947	2.293	1.783
μ [mm^{-1}]	11.395	6.739	7.071	11.596	6.072
F(000)	2024	1840	1832	4240	3936
Crystal size [mm^3]	0.23 x 0.13 x 0.10	0.33 × 0.19 × 0.10	0.16 x 0.12 x 0.10	0.20 x 0.20 x 0.05	0.30 x 0.23 x 0.08
θ range [deg]	3.07 – 27.48	2.24 – 25.00	2.66 – 25.00	2.19 – 25.03	2.61 – 25.03
Index ranges	–12 to 11,<br>–12 to 12,<br>–47 to 47	–15 to 15,<br>–17 to 17,<br>-21 to 21	–18 to 18,<br>–16 to 16,<br>–19 to 19	–38 to 38,<br>–18 to 18,<br>–16 to 16	–16 to 16,<br>–19 to 18,<br>–40 to 40
Reflections collected	59146	38505	60215	179710	107947
Unique data	3822(R_{int} = 0.050)	3005 (R_{int} = 0.113)	5729(R_{int} = 0.100)	5790(R_{int} = 0.066)	6694(R_{int} = 0.143)
Reflections [I>2σ(I)]	3731	2088	4506	5401	4563
Goodness-of-fit on F^2	1.173	1.135	1.075	1.032	1.009
Final R indices [I>2σ(I)]	R1 = 0.018<br>wR2 = 0.035	R1 = 0.047<br>wR2 = 0.106	R1 = 0.039<br>wR2 = 0.093	R1 = 0.013<br>wR2 = 0.031	R1 = 0.035<br>wR2 = 0.106
R indices (all data)	R1 = 0.019<br>wR2 = 0.035	R1 = 0.083<br>wR2 = 0.129	R1 = 0.062<br>wR2 = 0.111	R1 = 0.016<br>wR2 = 0.033	R1 = 0.095<br>wR2 = 0.140
Largest diff. peak/hole [e·$Å^{-3}$]	0.422/ -0.447	2.648/–2.139	2.575/ -1.608	0.629 / -0.511	2.217/ -2.788

[a] $R1 = \Sigma||F_0|-|F_c||/[\Sigma|F_0|]$ $wR2 = \{[\Sigma w(F_0^2 - F_c^2)^2] / [\Sigma w(F_0^2)^2]\}^{1/2}$

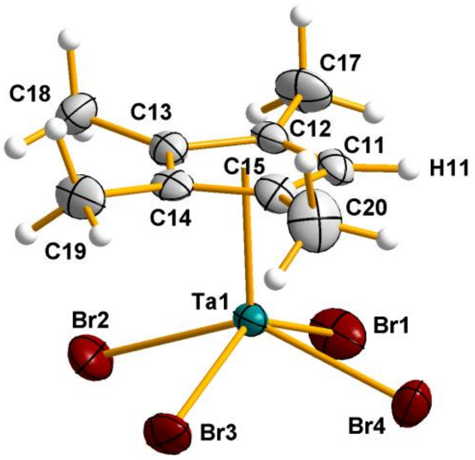

Figure S1: Molecular structure of compound [Ta(η^5-C_5HMe_4)Br_4]. Thermal ellipsoids are at 50% probability. Selected bond lengths (Å) and angles (deg): Ta1-Br1 2.5025(7), Ta1-Br2 2.522(8), Ta1-Br3 2.514(1), Ta1-Br4 2.515(1), Br-Ta1-Br averaged 83.3(5).

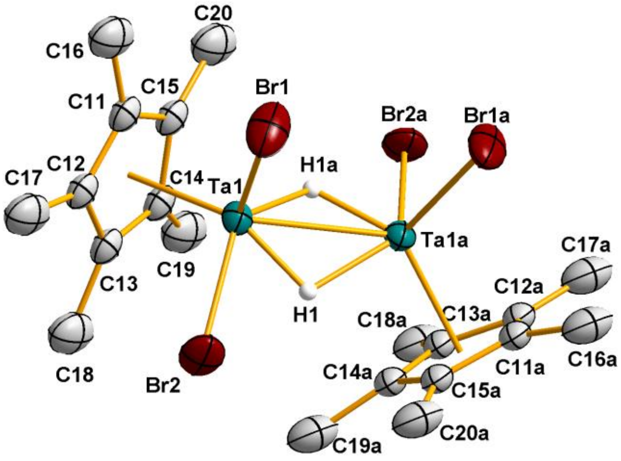

Figure S2: Molecular structure of compound **1Br**. Thermal ellipsoids are at 50% probability. Hydrogen atoms of η^5-C_5Me_5 ligands are omitted for clarity.

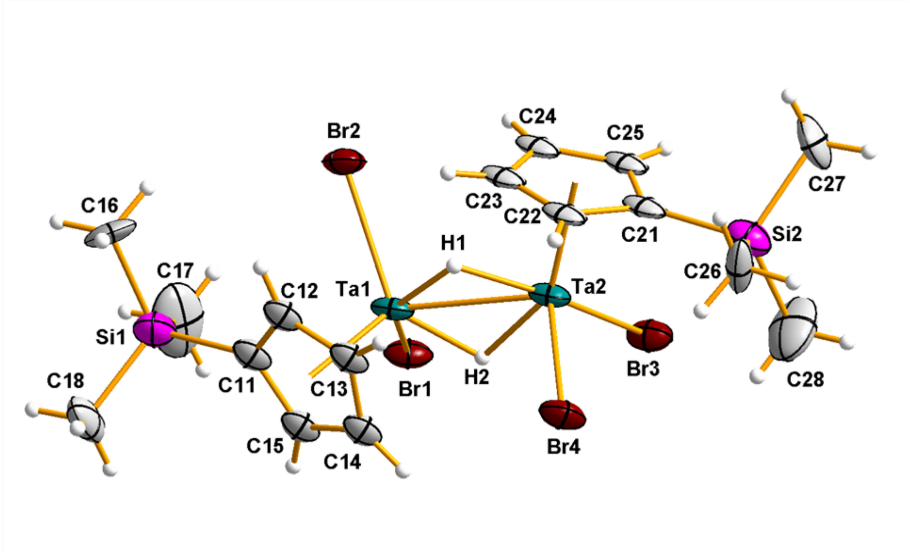

Figure S3: Molecular structure of compound **2Br**. Thermal ellipsoids are at 50% probability.

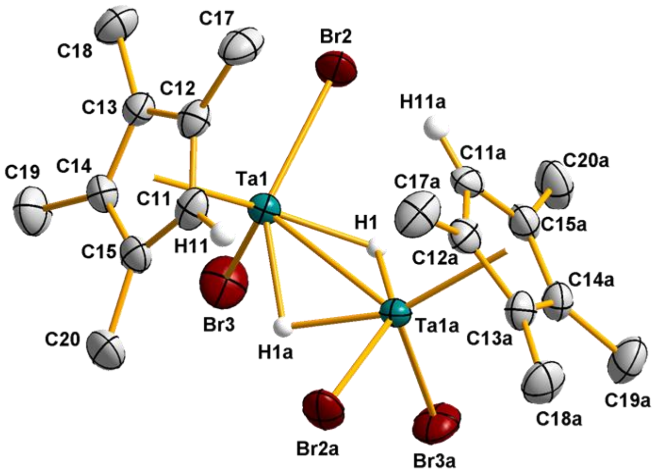

Figure S4: Molecular structure of compound **3Br**. Thermal ellipsoids are at 50% probability. Hydrogen atoms of methyl groups are omitted for clarity.

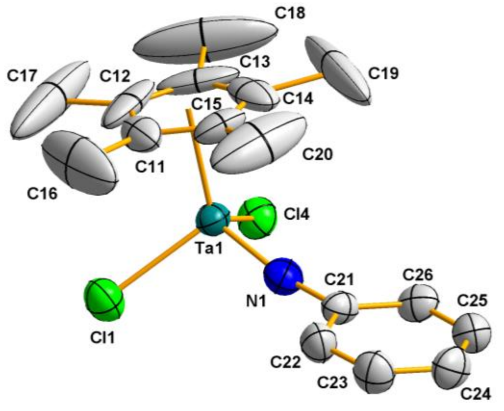

Figure S5. Molecular structure of compound **4**. Thermal ellipsoids are at 50% probability. Hydrogen atoms are omitted for clarity. Selected averaged bond lengths (Å) and angles (deg) found for complex **4**: Ta1-N1 1.780(9), Ta1-Cl 2.333(1), N1-C21 1.400(13), Ta1-N1-C21 167.7(8), N1-Ta1-Cl 103.4(2), Cl-Ta1-Cl 104.0(1).

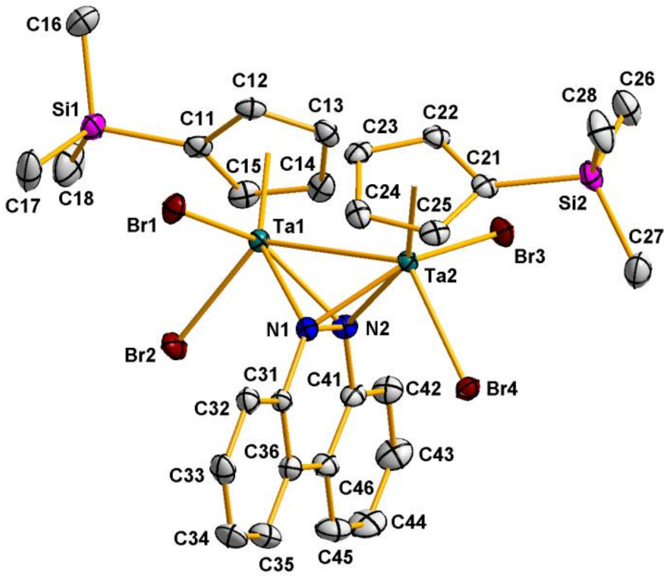

Figure S6: Molecular structure of compound **8Br**. Thermal ellipsoids are at 50% probability. Hydrogen atoms are omitted for clarity.

NMR Spectroscopy

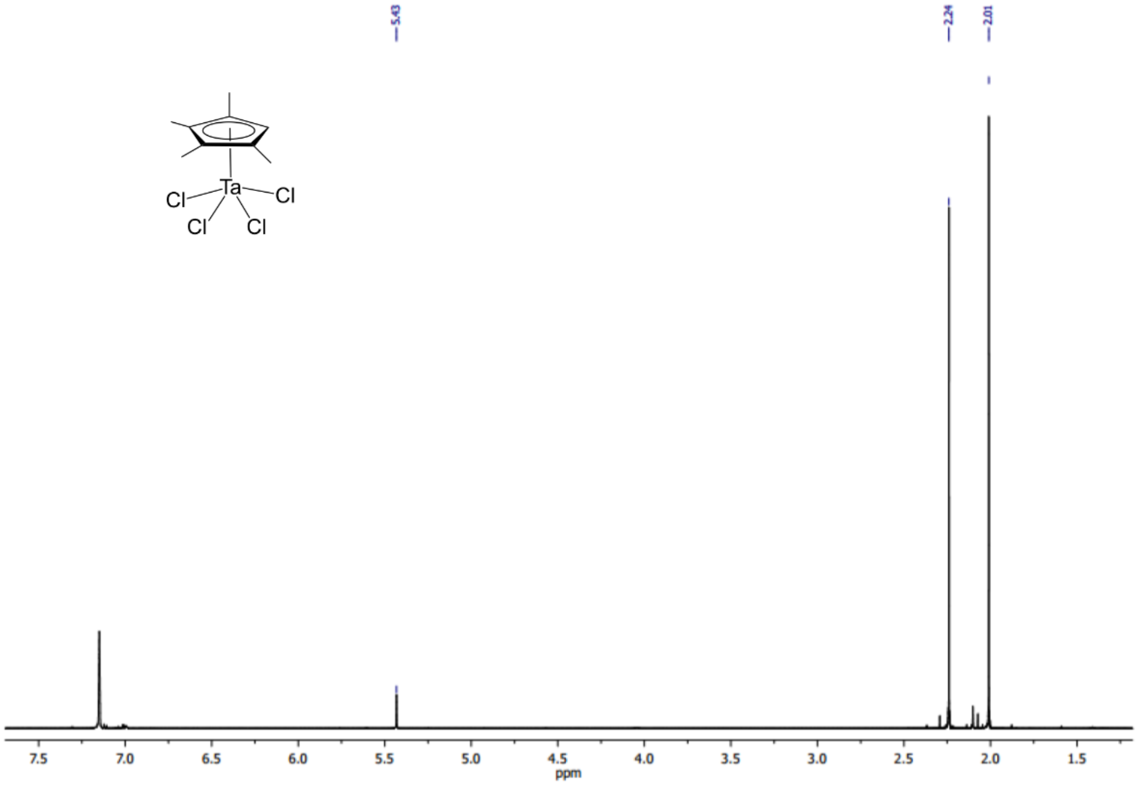

Figure S7. ^{1}H NMR spectrum of compound [Ta(η^5-C_5HMe_4)Cl_4] in C_6D_6 (500 MHz).

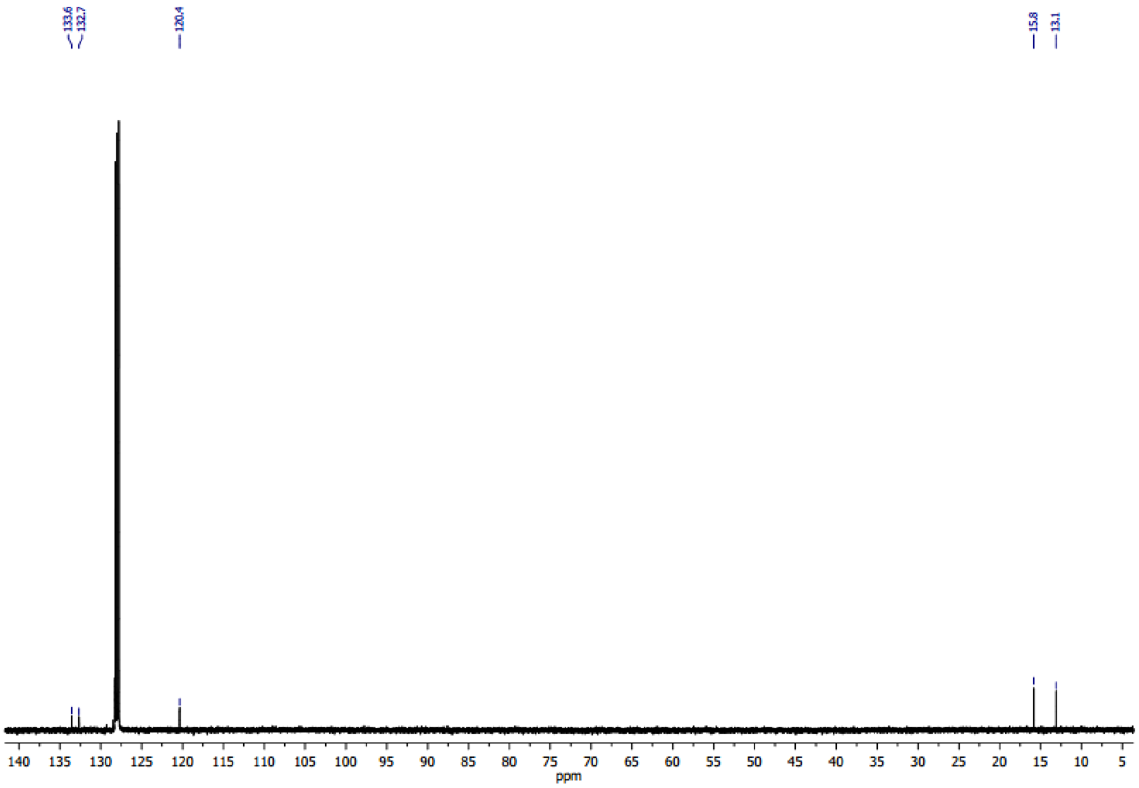

Figure S8. ^{13}C NMR spectrum of compound [Ta(η^5-C_5HMe_4)Cl_4] in C_6D_6 (125 MHz).

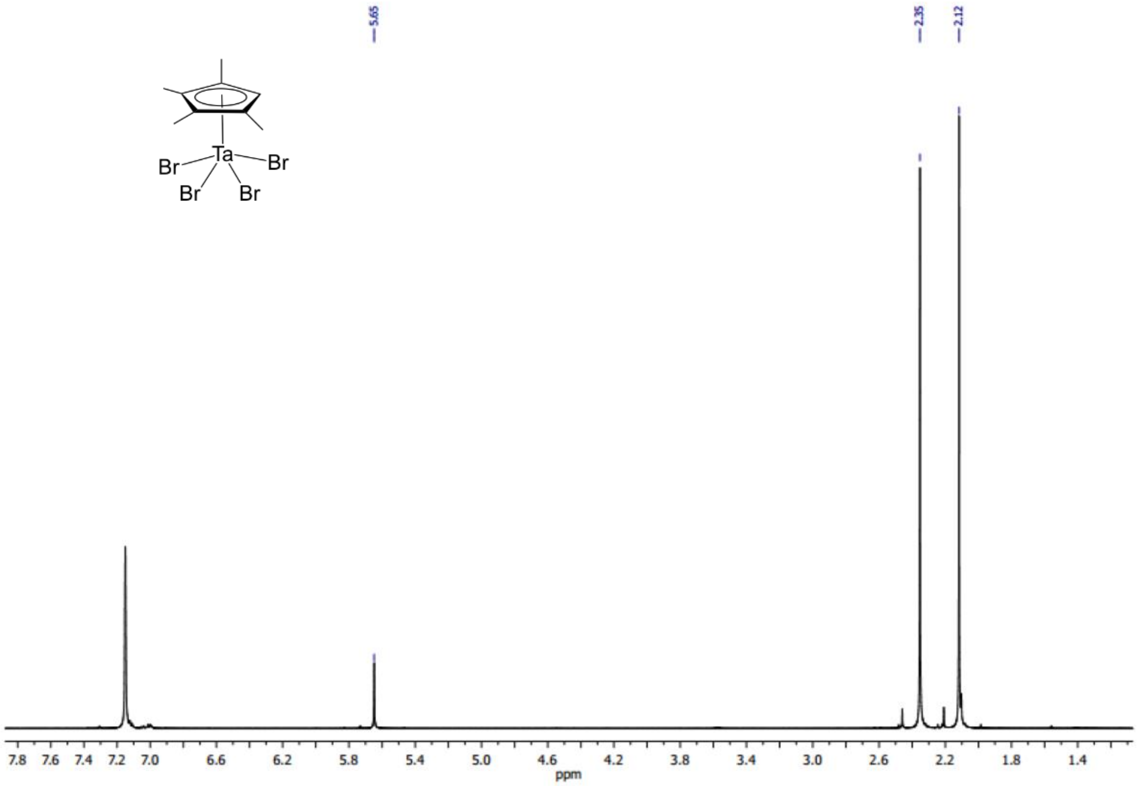

Figure S9. ^{1}H NMR spectrum of compound [Ta(η^5-C_5HMe_4)Br_4] in C_6D_6 (500 MHz).

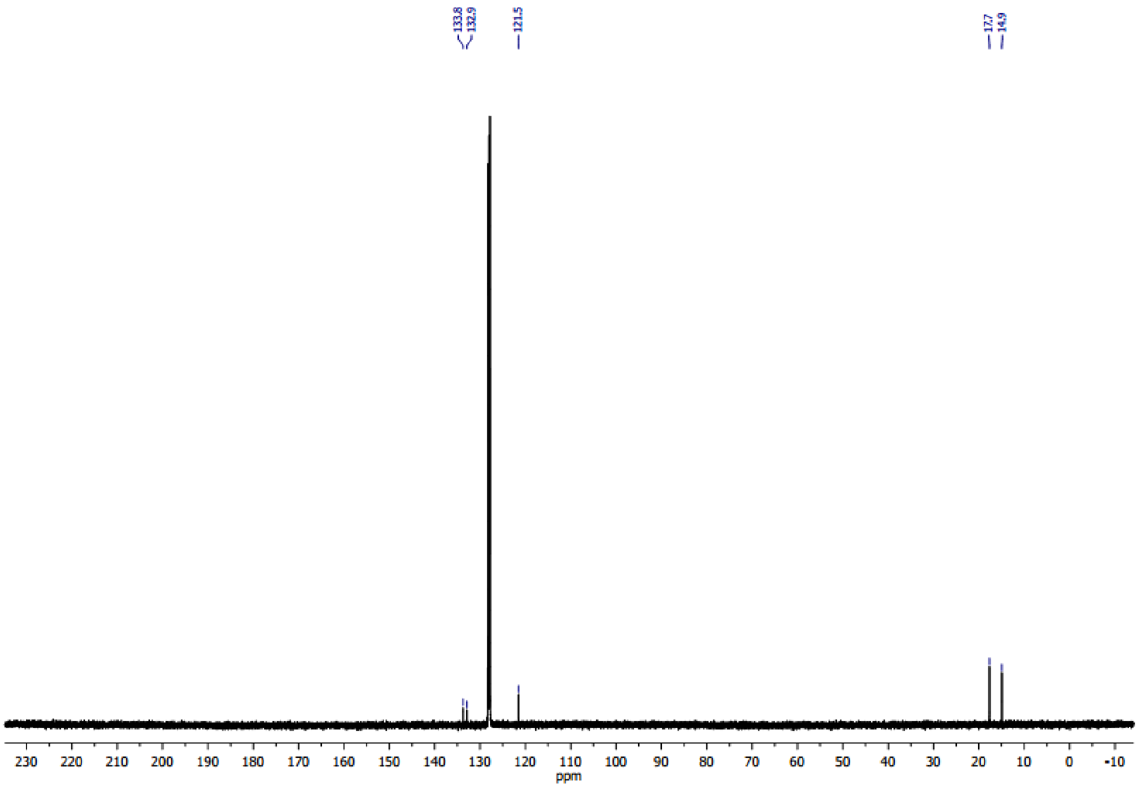

Figure S10. ^{13}C NMR spectrum of compound [Ta(η^5-C_5HMe_4)Br_4] in C_6D_6 (125 MHz).

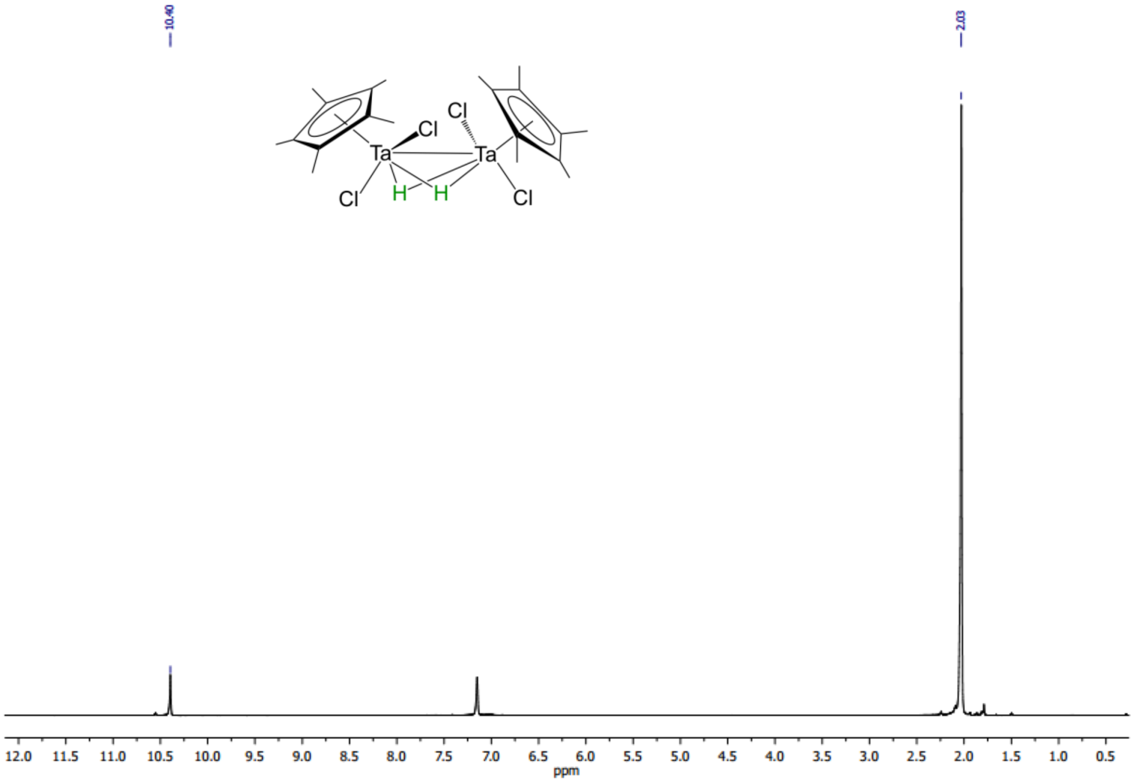

Figure S11. ^{1}H NMR spectrum of compound **1** in C_6D_6 (300 MHz).

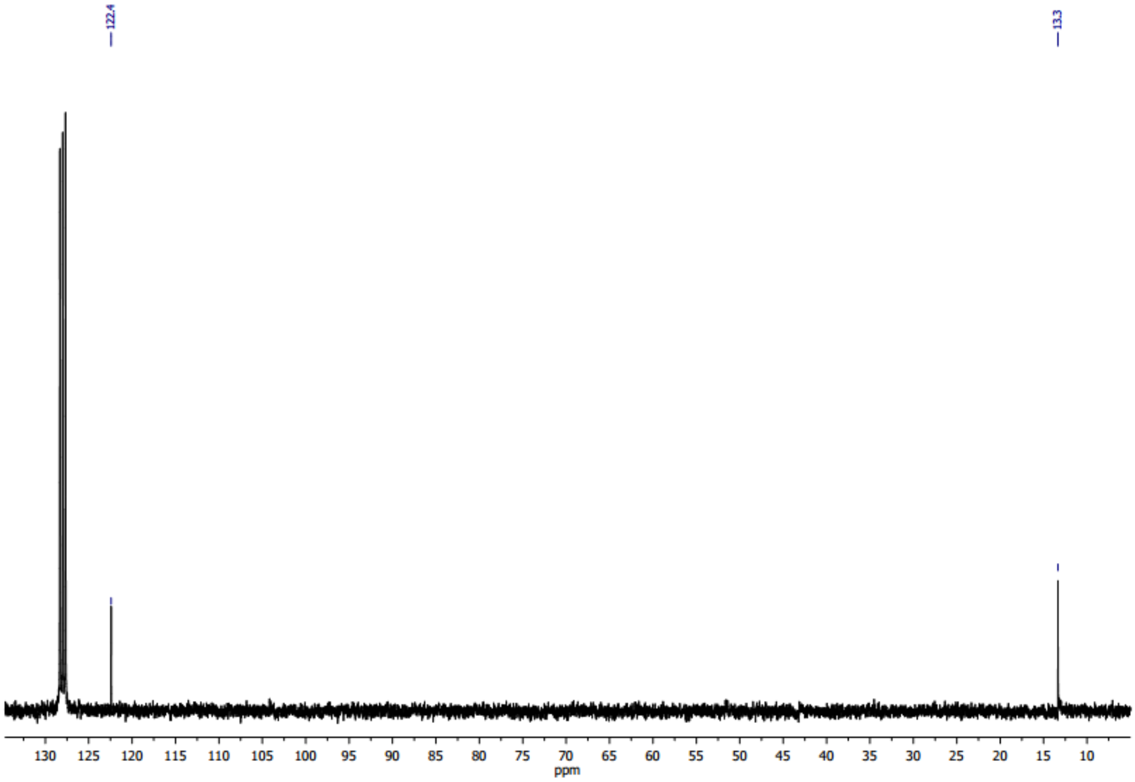

Figure S12. $^{13}C\{^{1}H\}$ NMR spectrum of compound **1** in C_6D_6 (75 MHz).

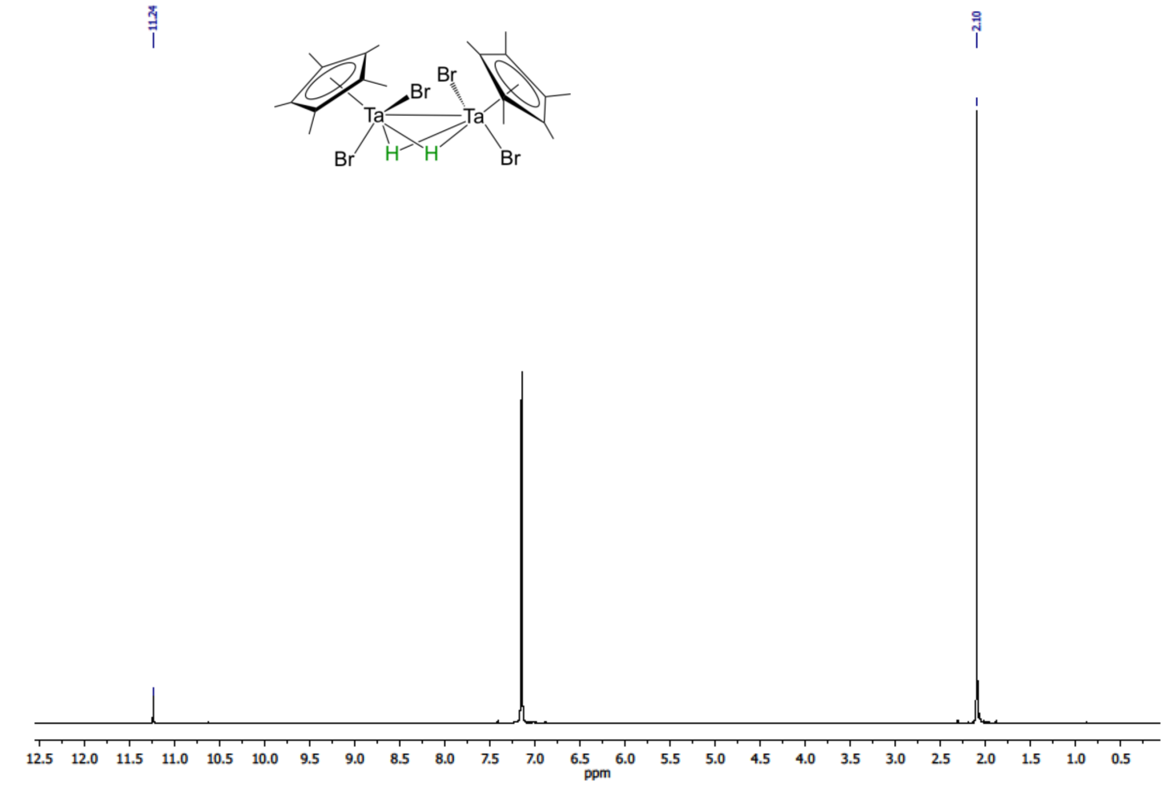

Figure S13. ^{1}H NMR spectrum of compound **1Br** in C_6D_6 (300 MHz).

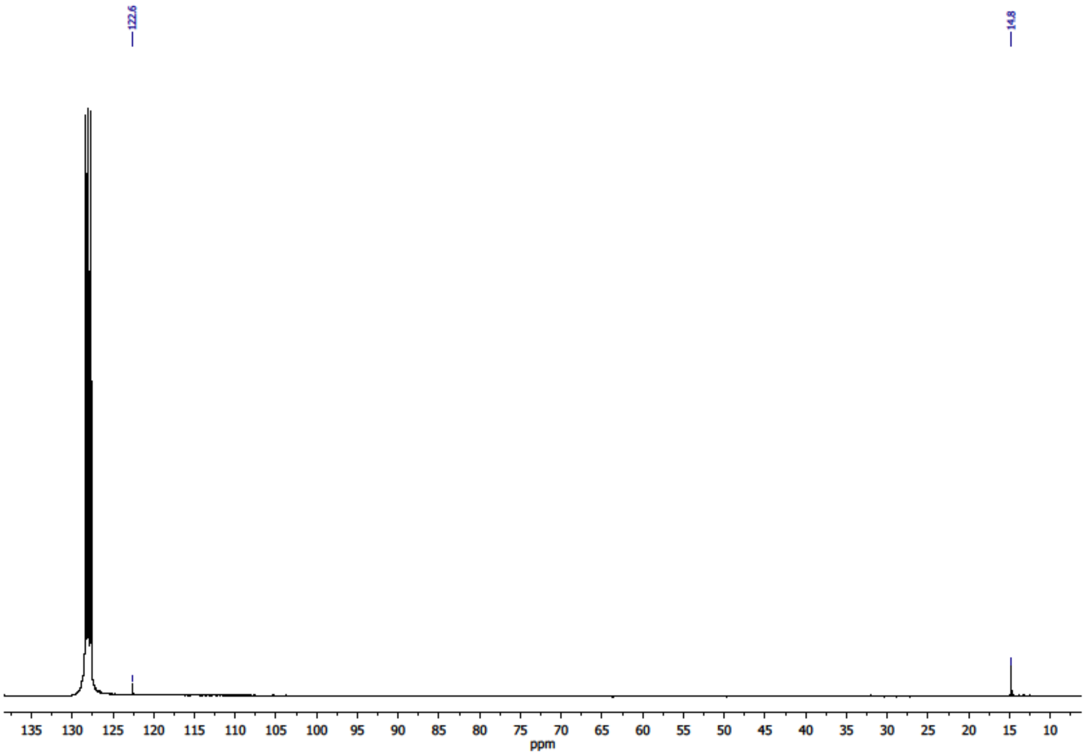

Figure S14. $^{13}C\{^{1}H\}$ NMR spectrum of compound **1Br** in C_6D_6 (75 MHz).

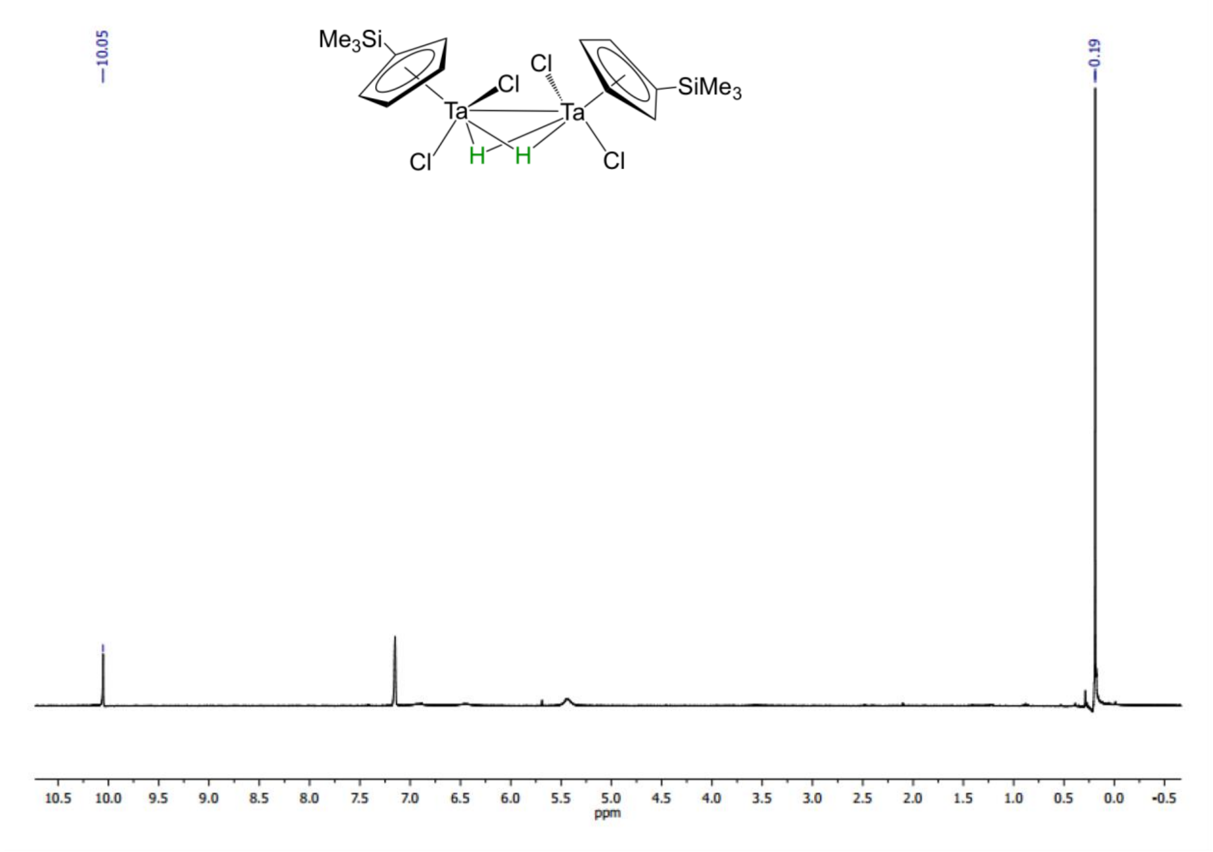

Figure S15. ^{1}H NMR spectrum of compound **2** in C_6D_6 (300 MHz).

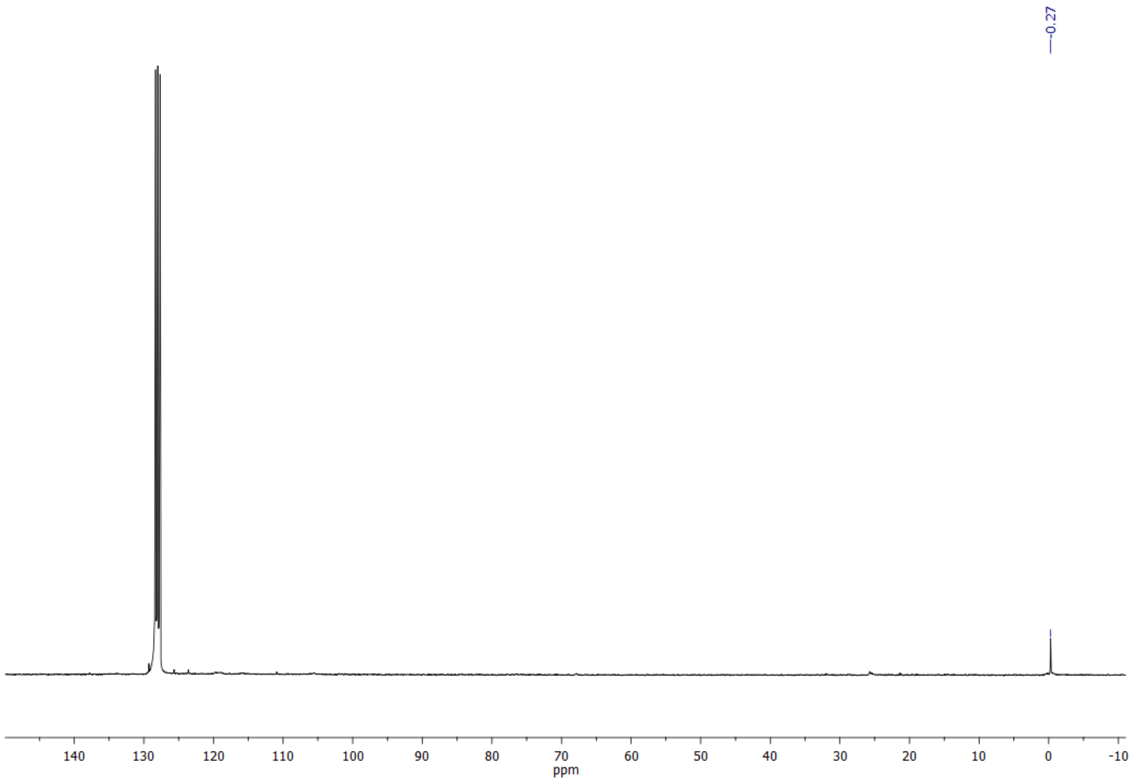

Figure S16. $^{13}C\{^{1}H\}$ NMR spectrum of compound **2** in C_6D_6 (75 MHz).

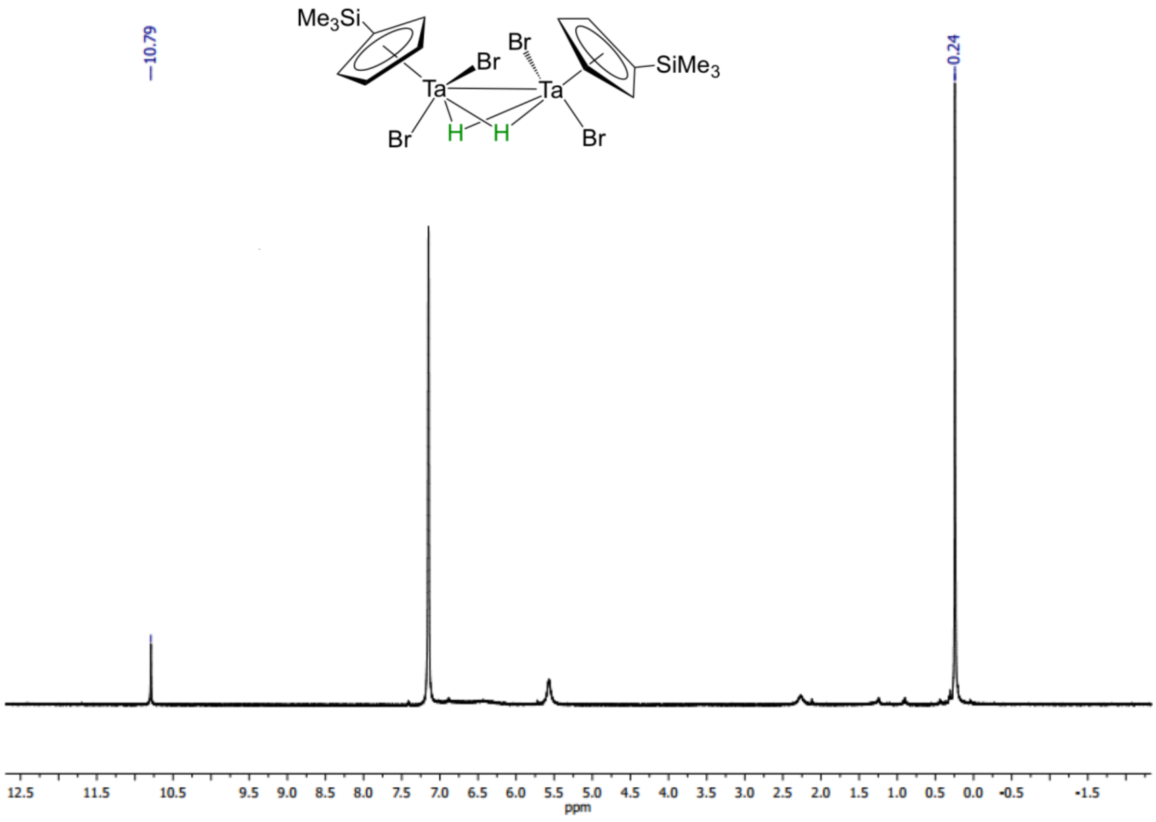

Figure S17. ^{1}H NMR spectrum of compound **2Br** in C_6D_6 (300 MHz).

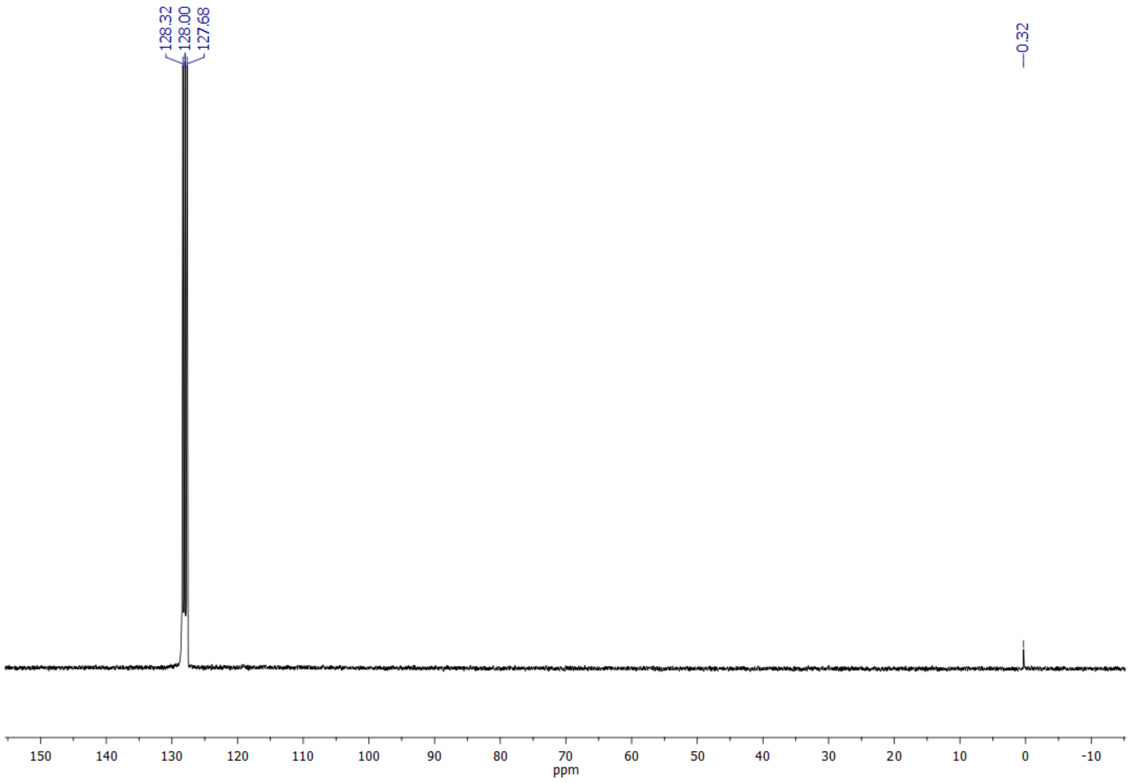

Figure S18. $^{13}C\{^{1}H\}$ NMR spectrum of compound **2Br** in C_6D_6 (75 MHz).

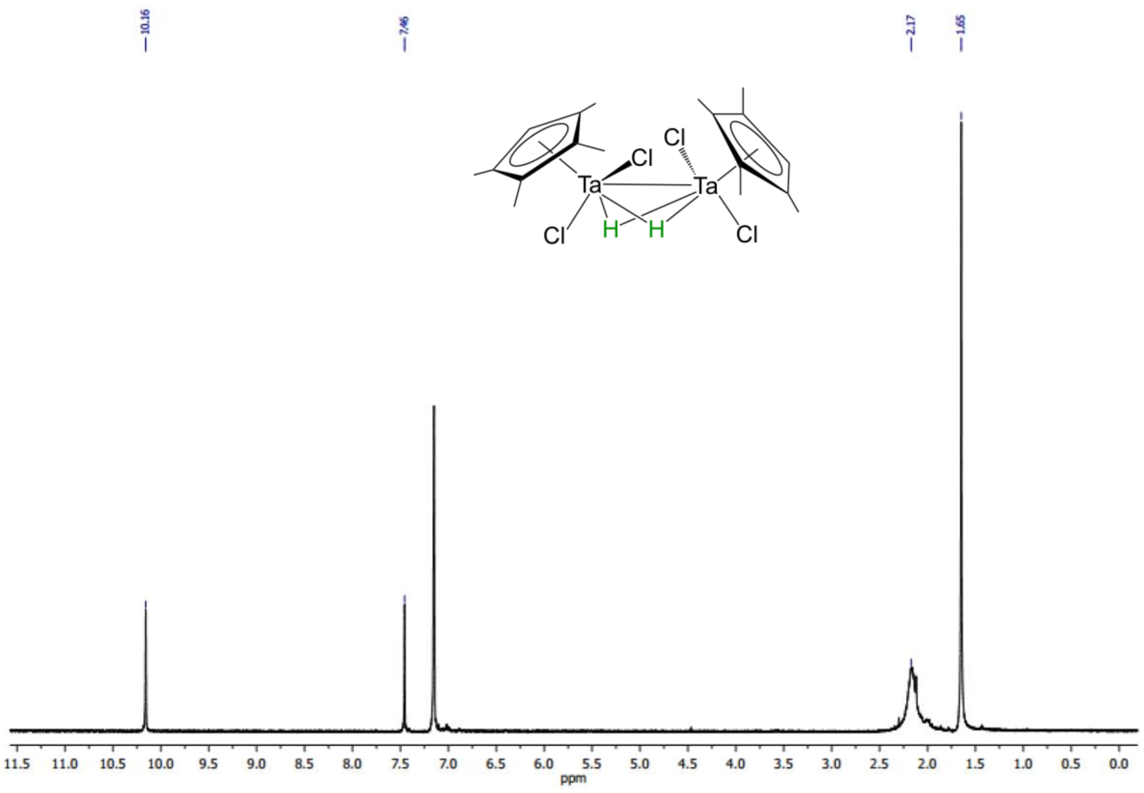

Figure S19. ^{1}H NMR spectrum of compound **3** in C_6D_6 (300 MHz).

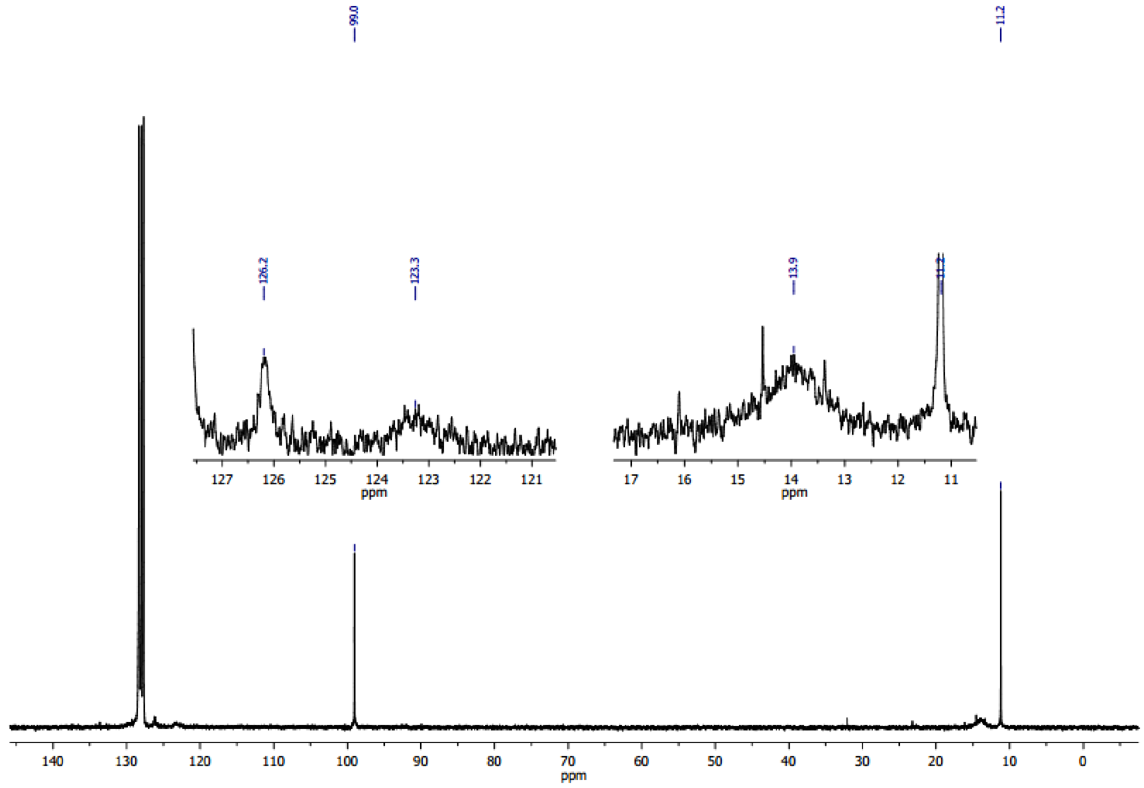

Figure S20. ^{13}C NMR spectrum of compound **3** in C_6D_6 (75 MHz).

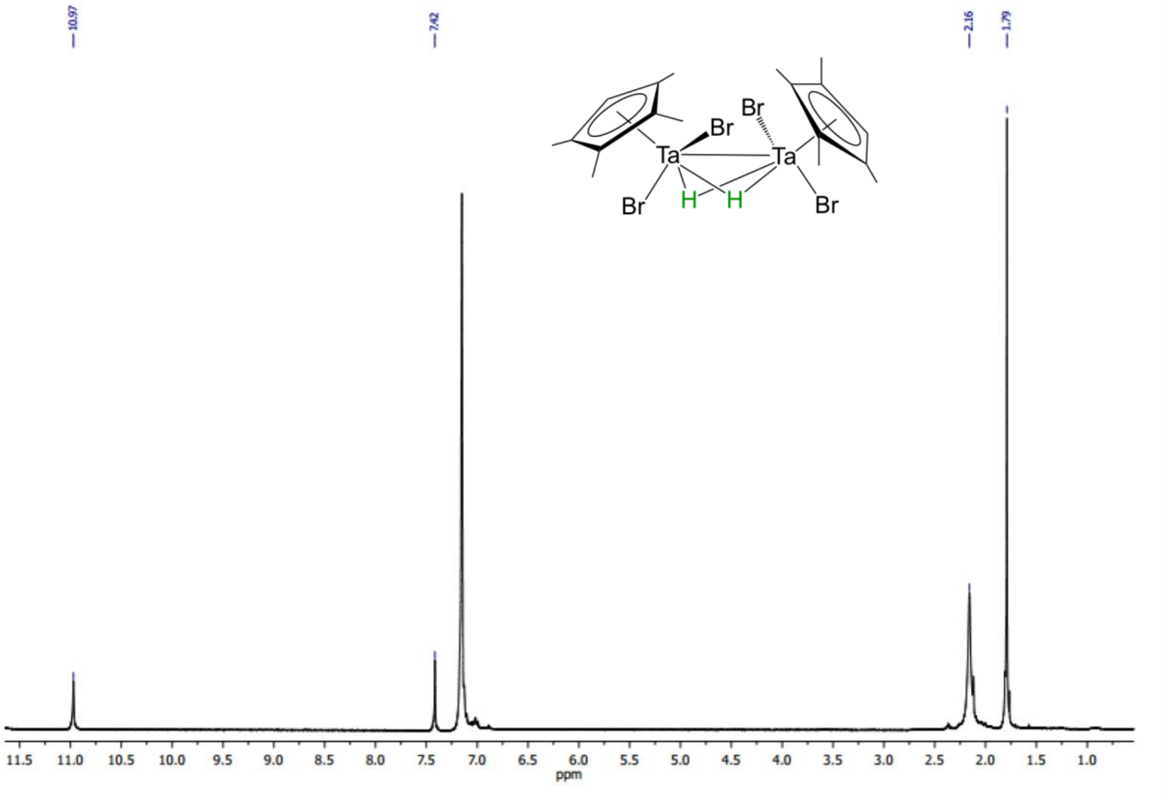

Figure S21. ^{1}H NMR spectrum of compound **3Br** in C_6D_6 (300 MHz).

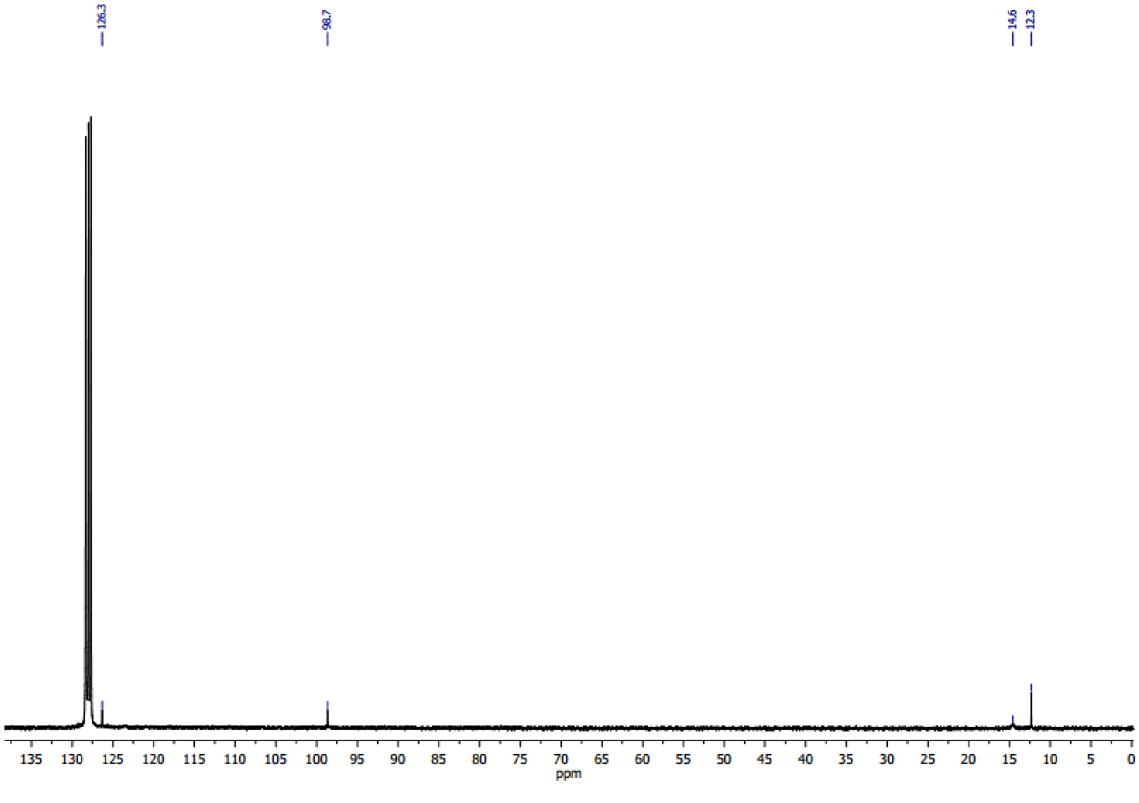

Figure S22. ^{13}C NMR spectrum of compound **3Br** in C_6D_6 (75 MHz).

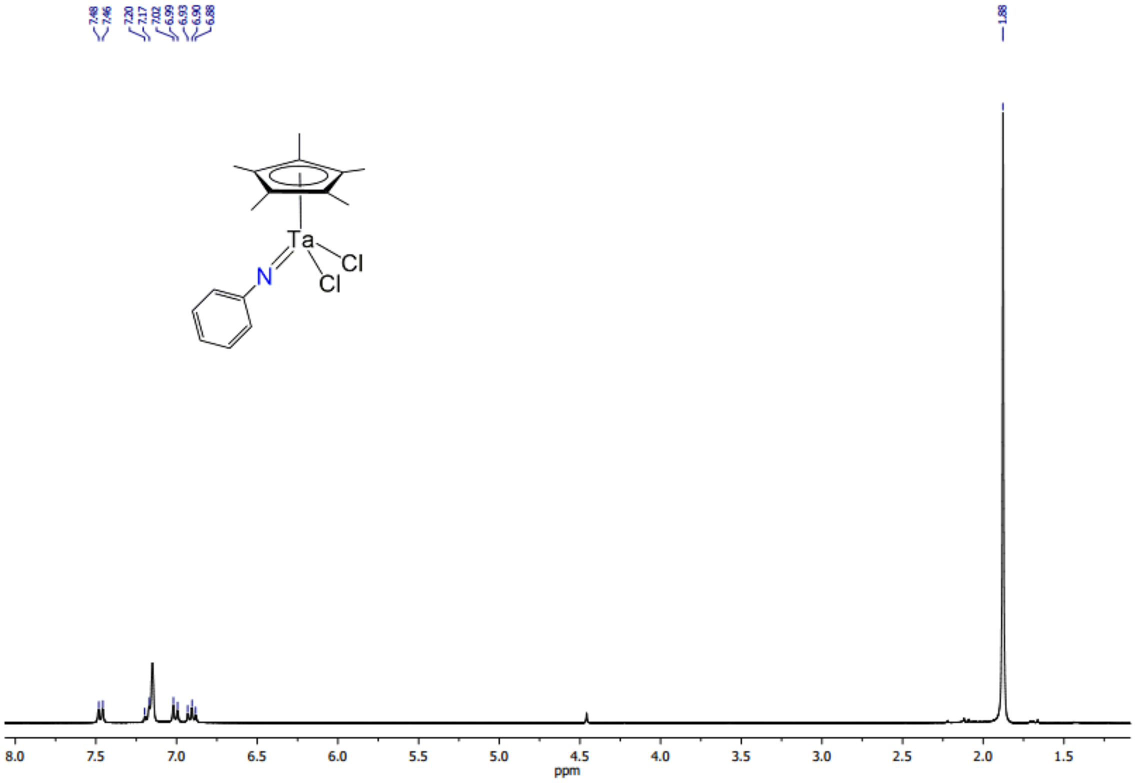

Figure S23. ^{1}H NMR spectrum of compound **4** in C_6D_6 (500 MHz).

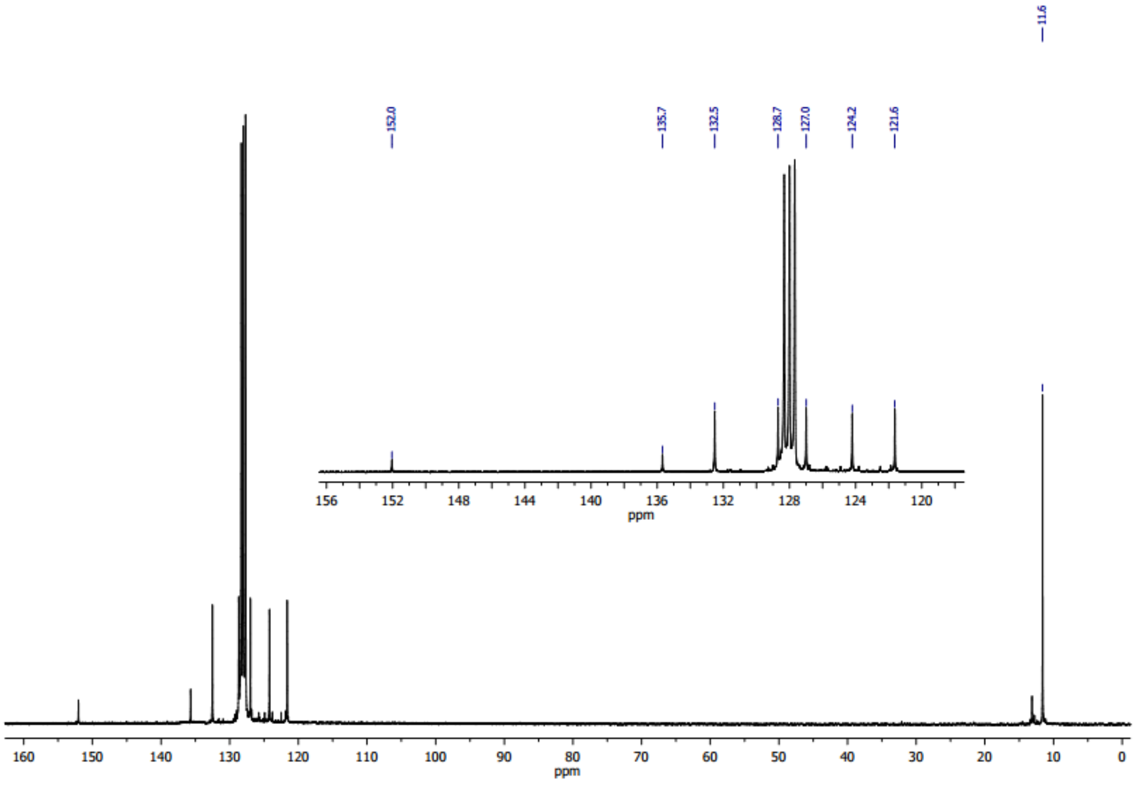

Figure S24. $^{13}C\{^{1}H\}$ NMR spectrum of compound **4** in C_6D_6 (125 MHz).

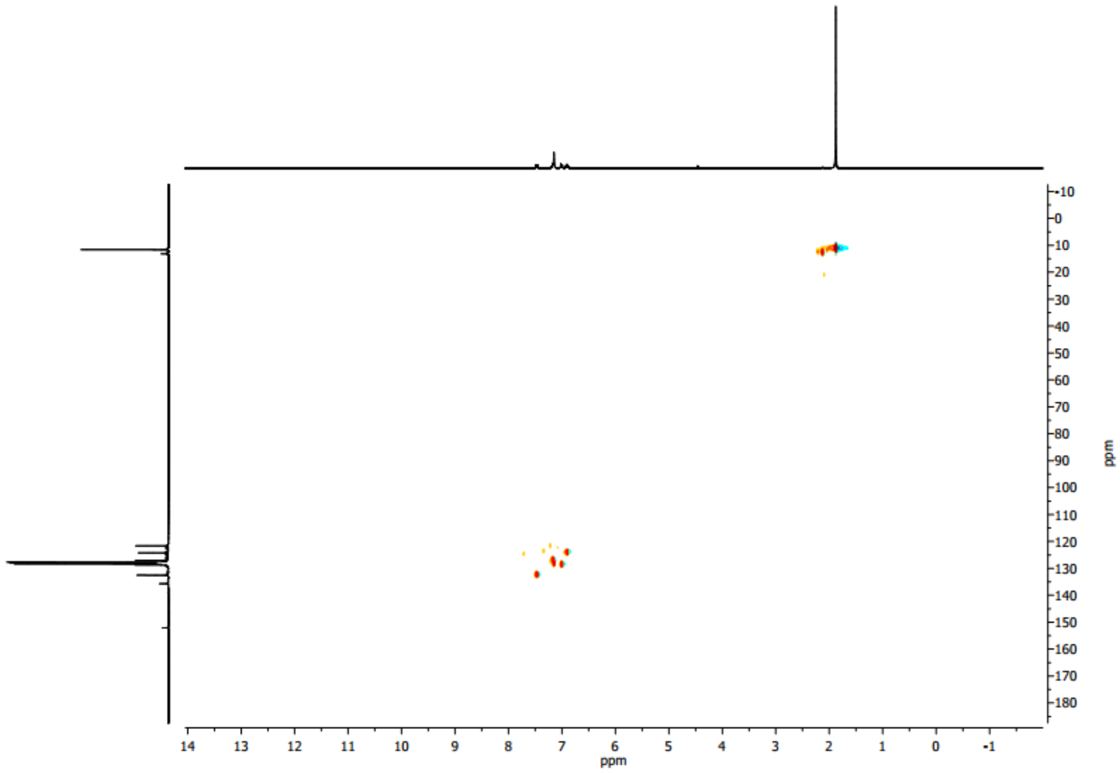

Figure S25. g-HSQC NMR spectrum of compound **4** in C_6D_6 (500 MHz).

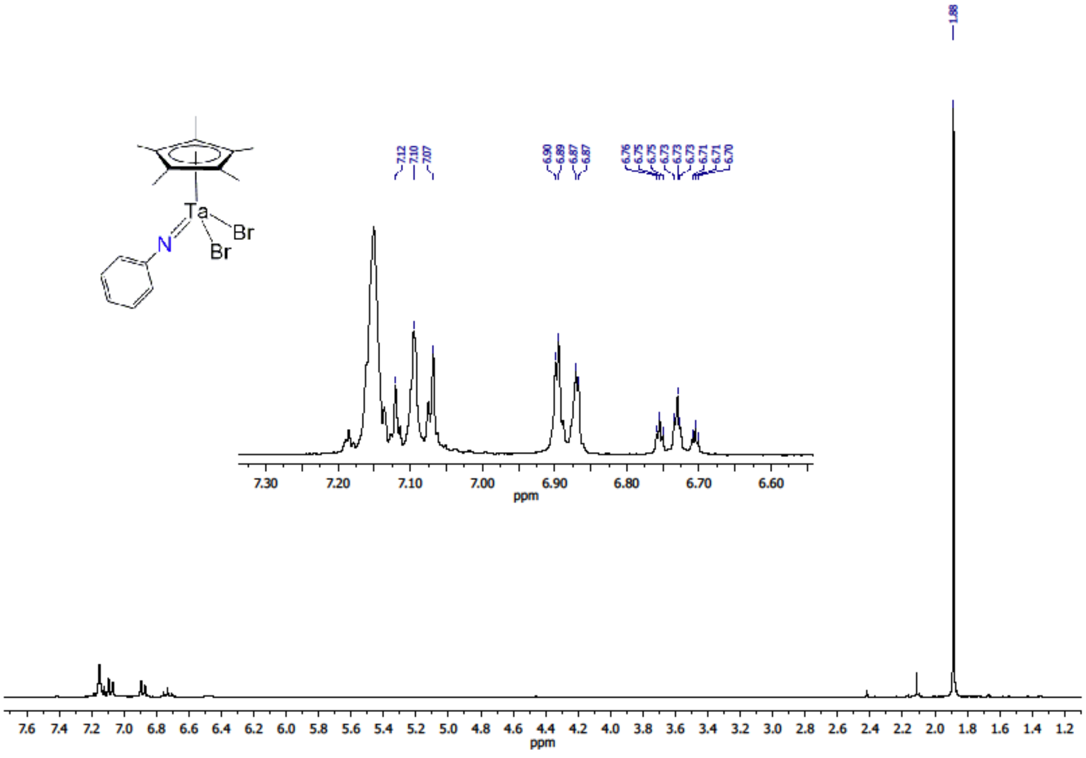

Figure S26. ^1H NMR spectrum of compound **4Br** in C_6D_6 (300 MHz).

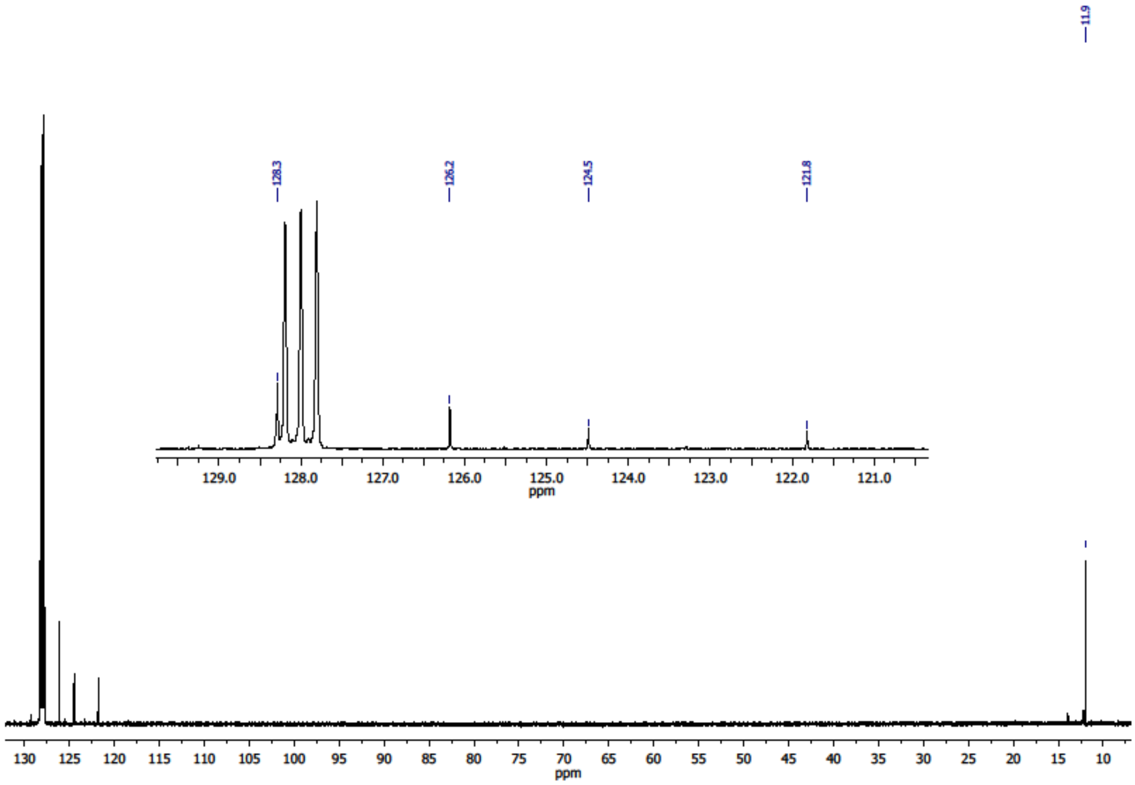

Figure S27. $^{13}C\{^{1}H\}$ NMR spectrum of compound **4Br** in C_6D_6 (75 MHz).

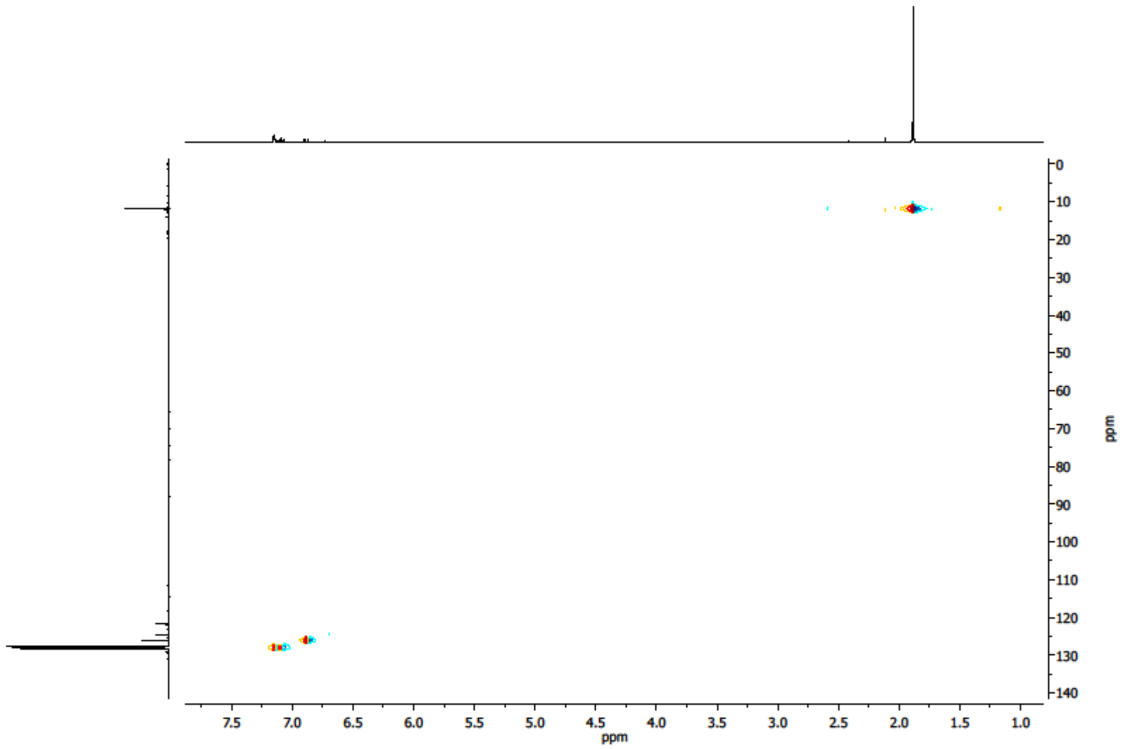

Figure S28. g-HSQC NMR spectrum of compound **4Br** in C_6D_6 (500 MHz).

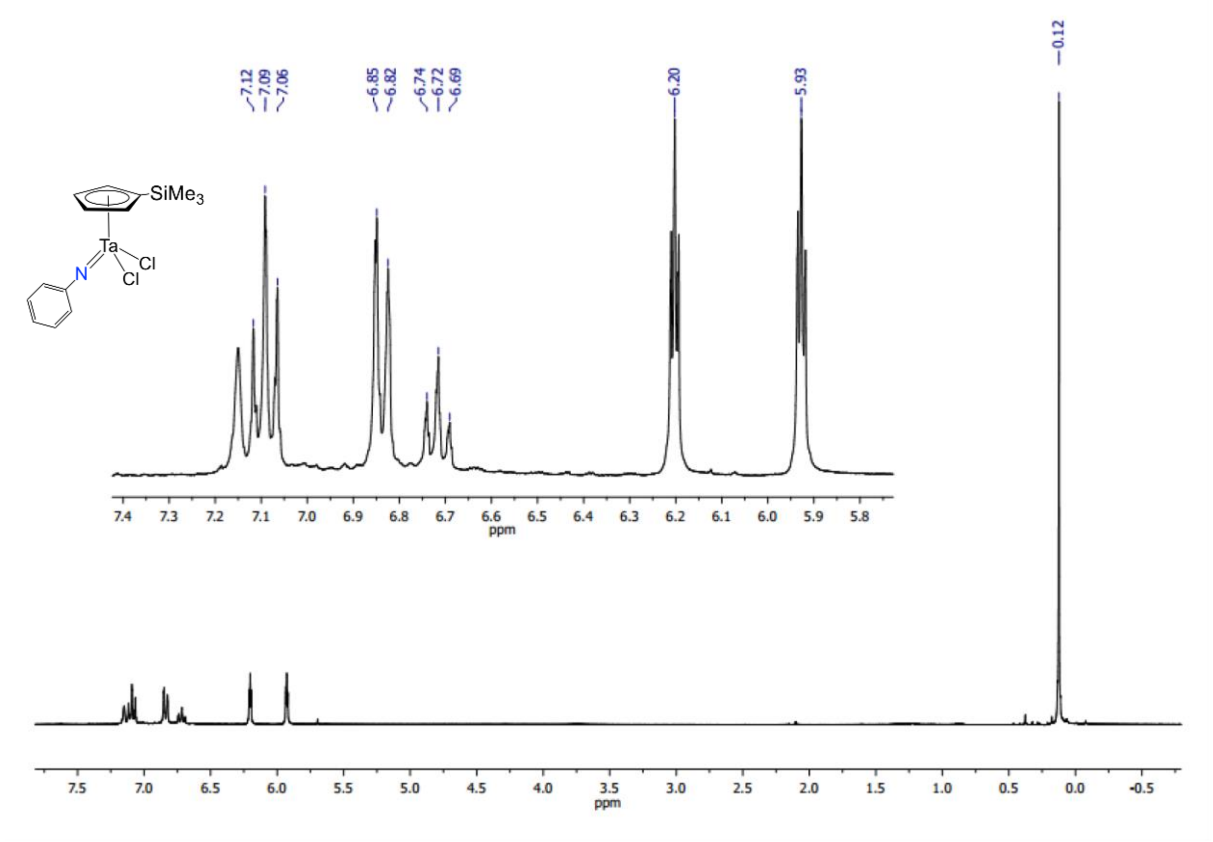

Figure S29. ^{1}H NMR spectrum of compound **5** in C_6D_6 (300 MHz).

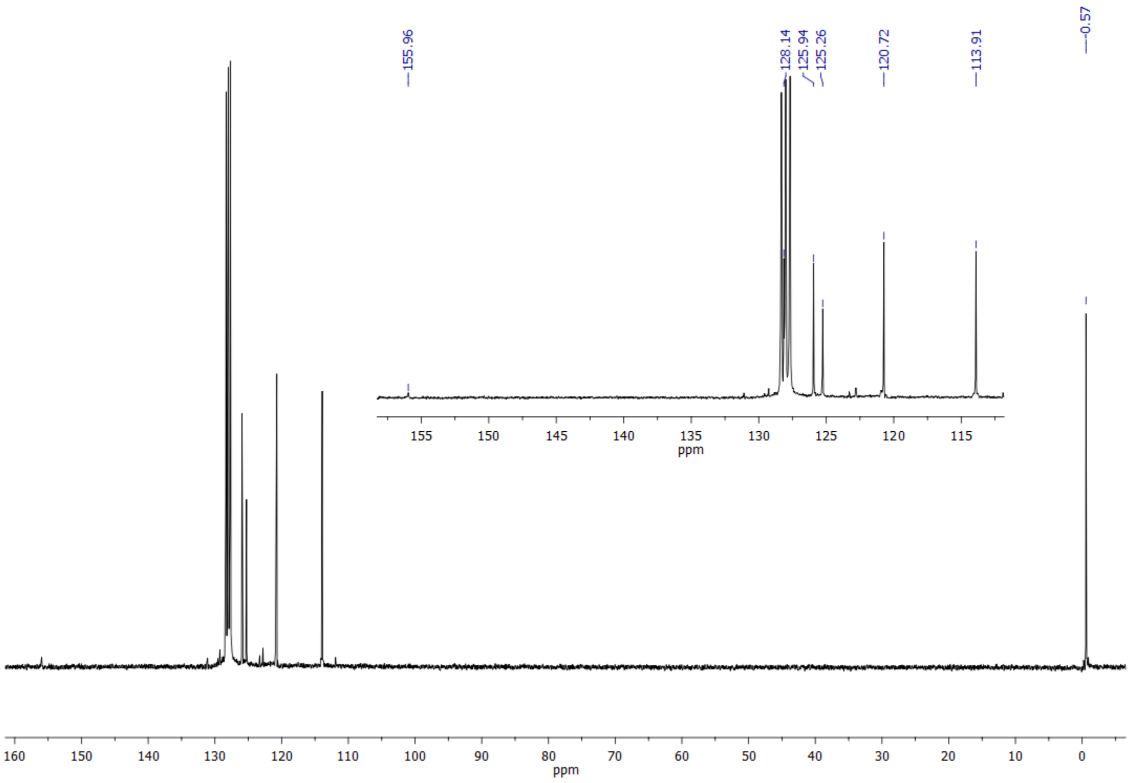

Figure S30. $^{13}C\{^{1}H\}$ NMR spectrum of compound **5** in C_6D_6 (75 MHz).

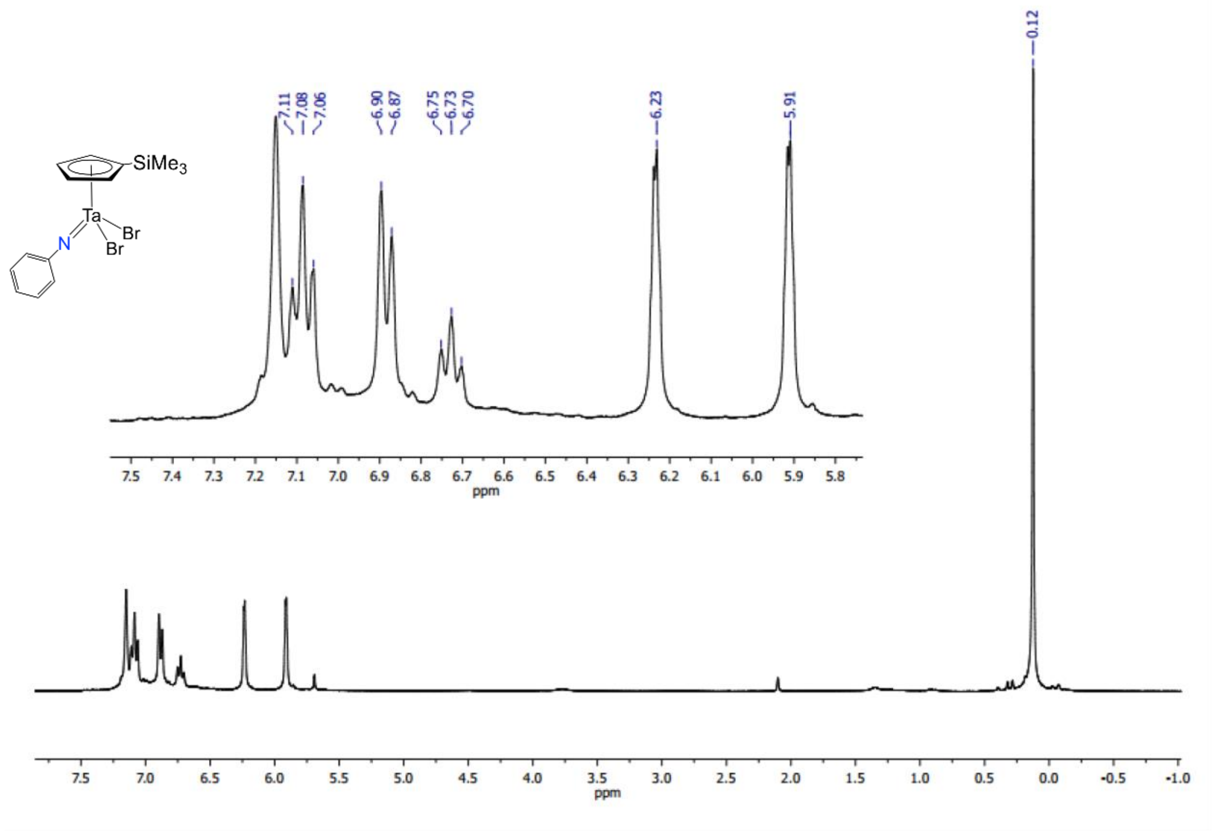

Figure S31. ^{1}H NMR spectrum of compound **5Br** in C_6D_6 (300 MHz).

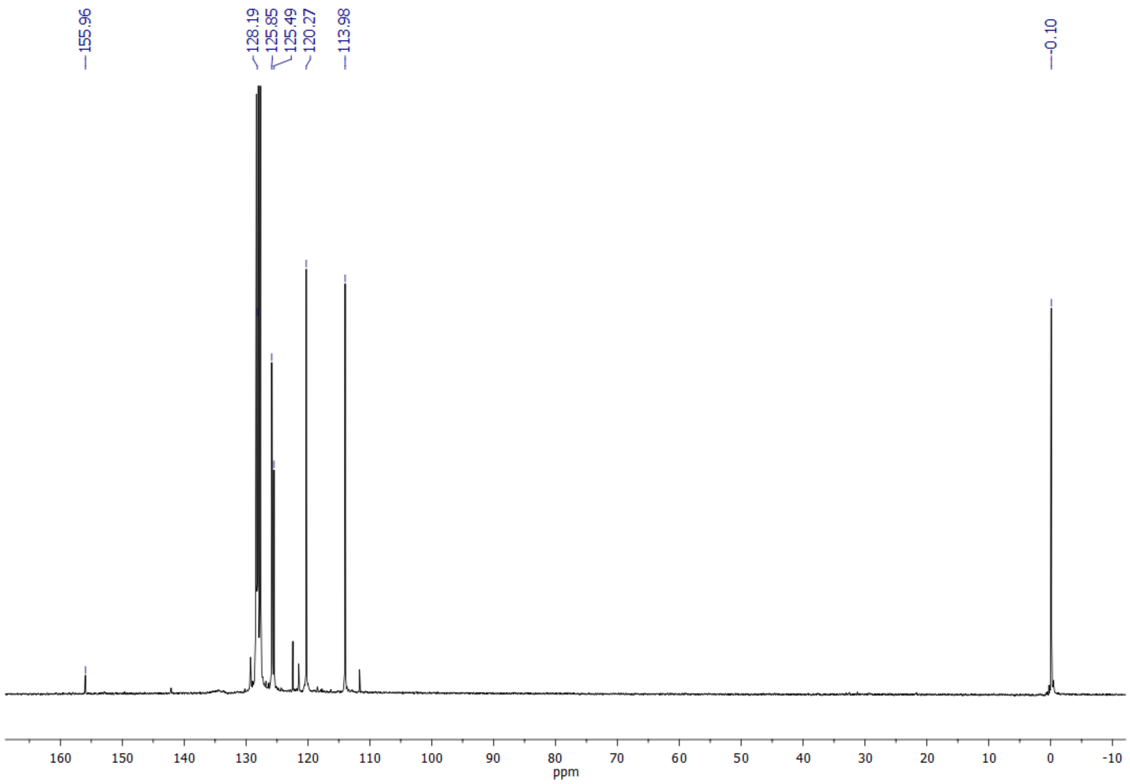

Figure S32. $^{13}C\{^{1}H\}$ NMR spectrum of compound **5Br** in C_6D_6 (75 MHz).

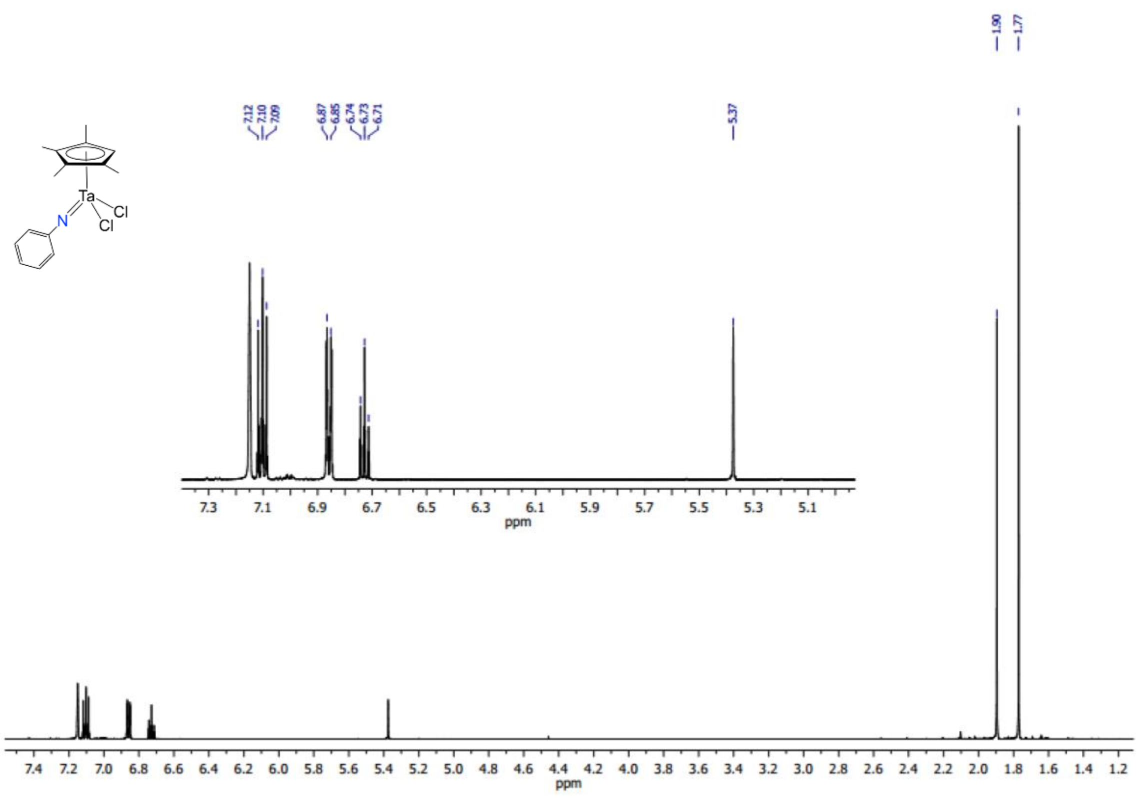

Figure S33. ^{1}H NMR spectrum of compound **6** in C_6D_6 (500 MHz).

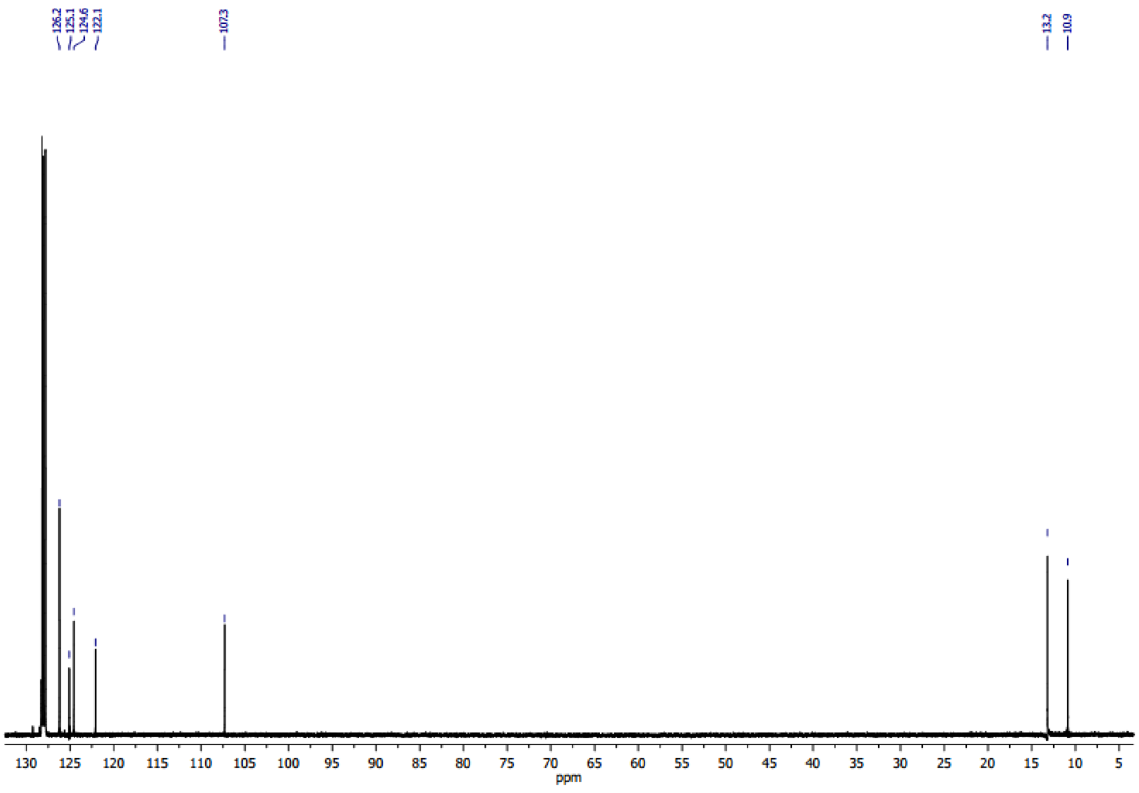

Figure S34. ^{13}C NMR spectrum of compound **6** in C_6D_6 (125 MHz).

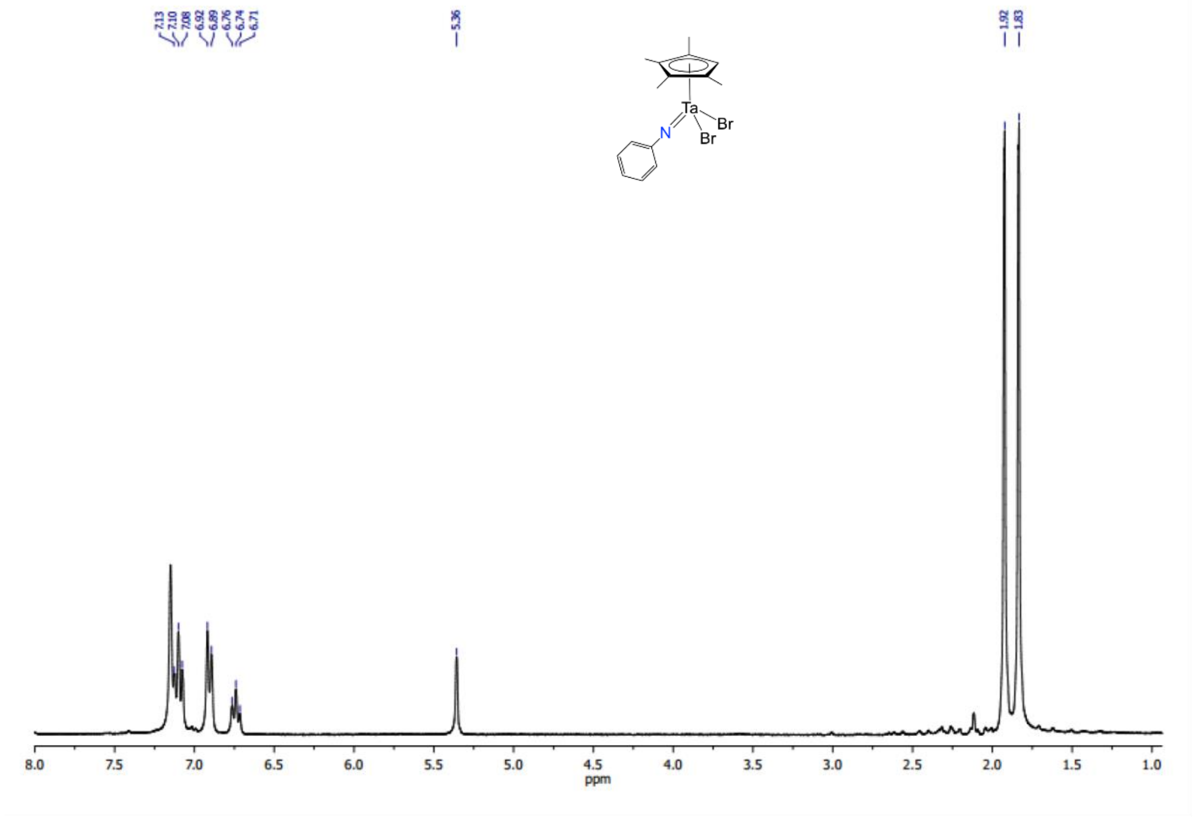

Figure S35. ^{1}H NMR spectrum of compound **6Br** in C_6D_6 (300 MHz).

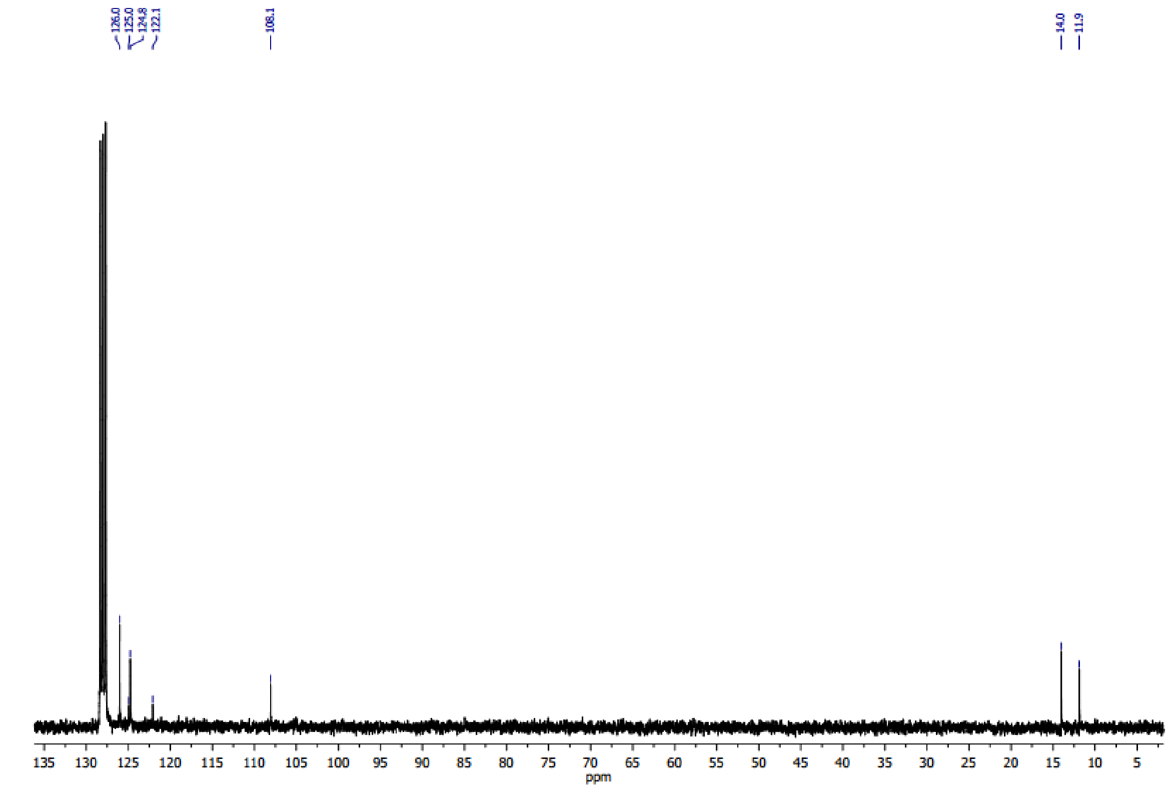

Figure S36. ^{13}C NMR spectrum of compound **6Br** in C_6D_6 (75 MHz).

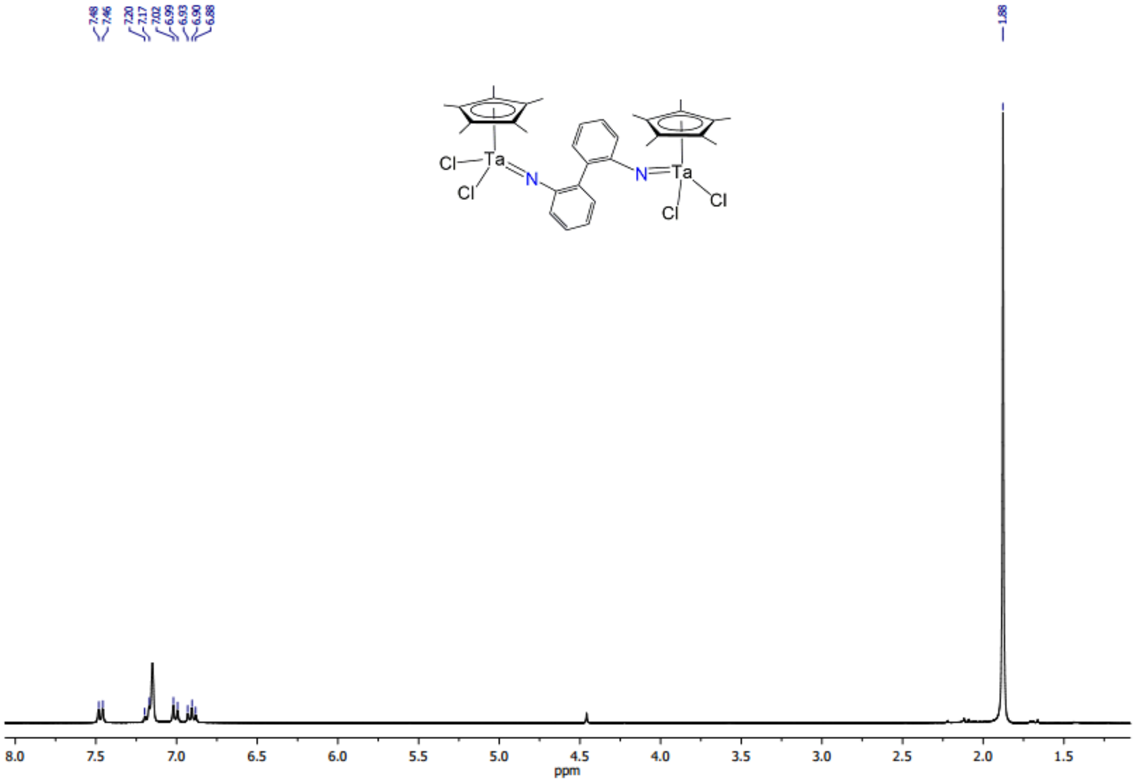

Figure S37. ^{1}H NMR spectrum of compound **7** in C_6D_6 (500 MHz).

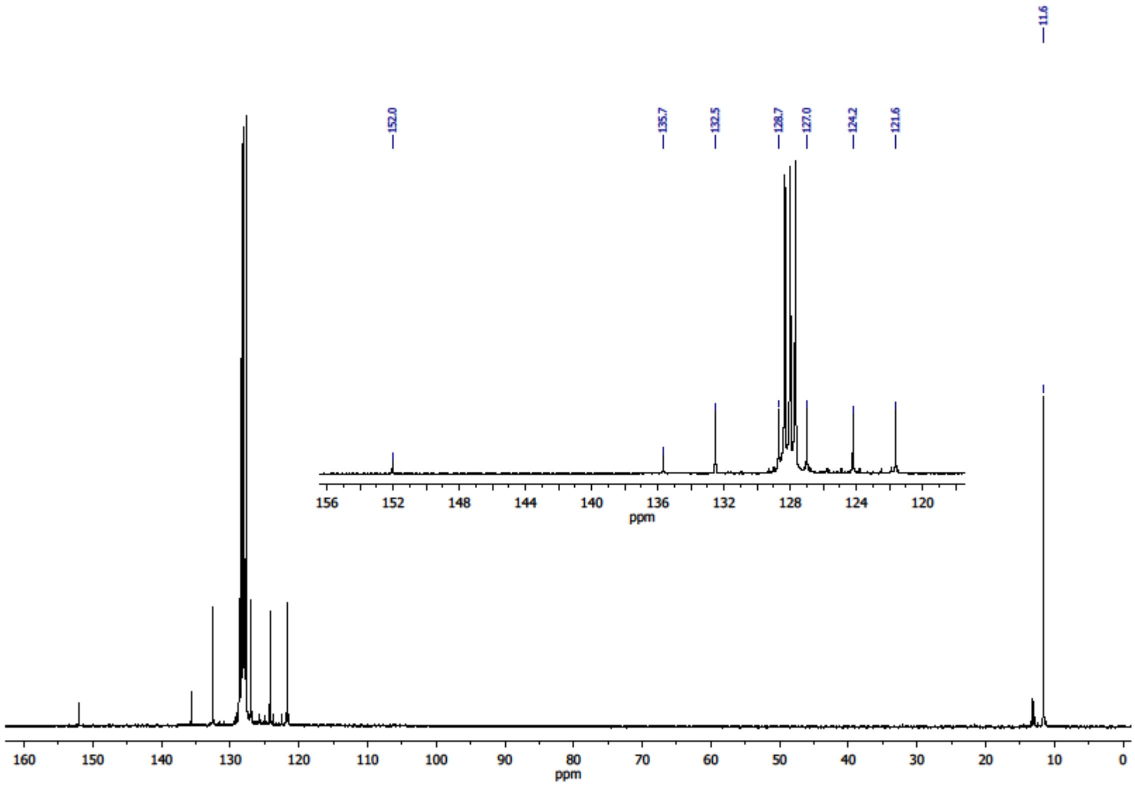

Figure S38. $^{13}C\{^{1}H\}$ NMR spectrum of compound **7** in C_6D_6 (125 MHz).

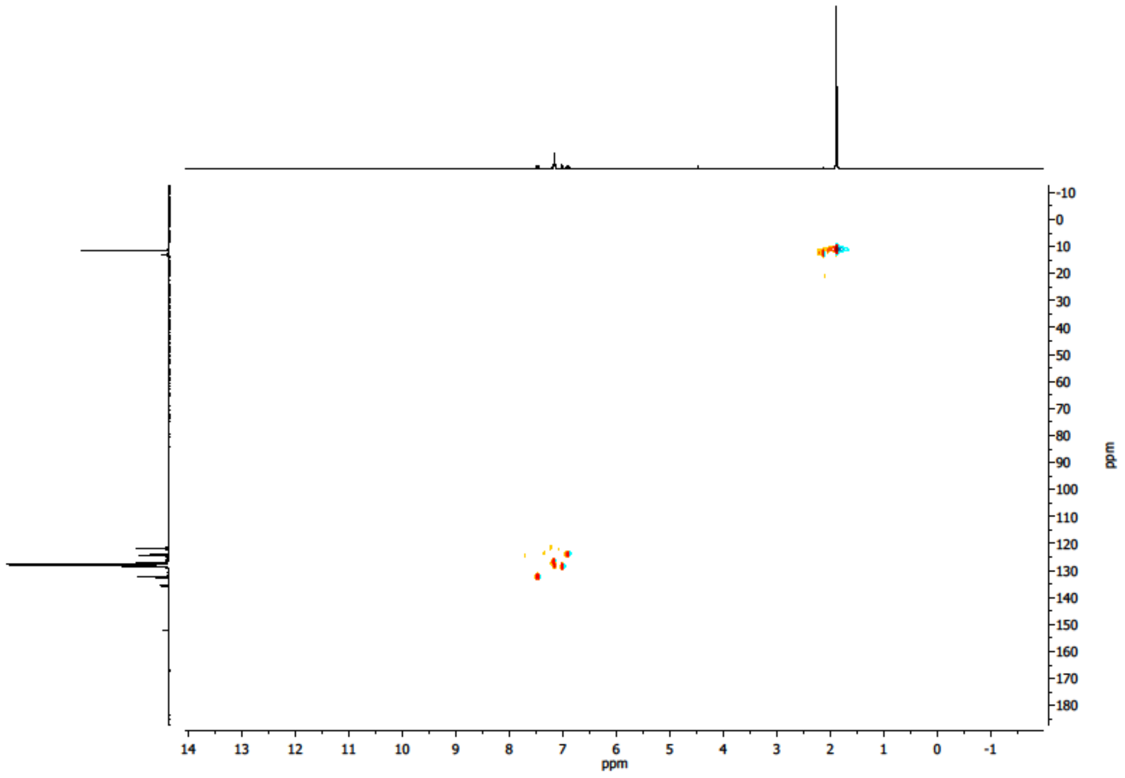

Figure S39. g-HSQC NMR spectrum of compound **7** in C_6D_6 (500 MHz).

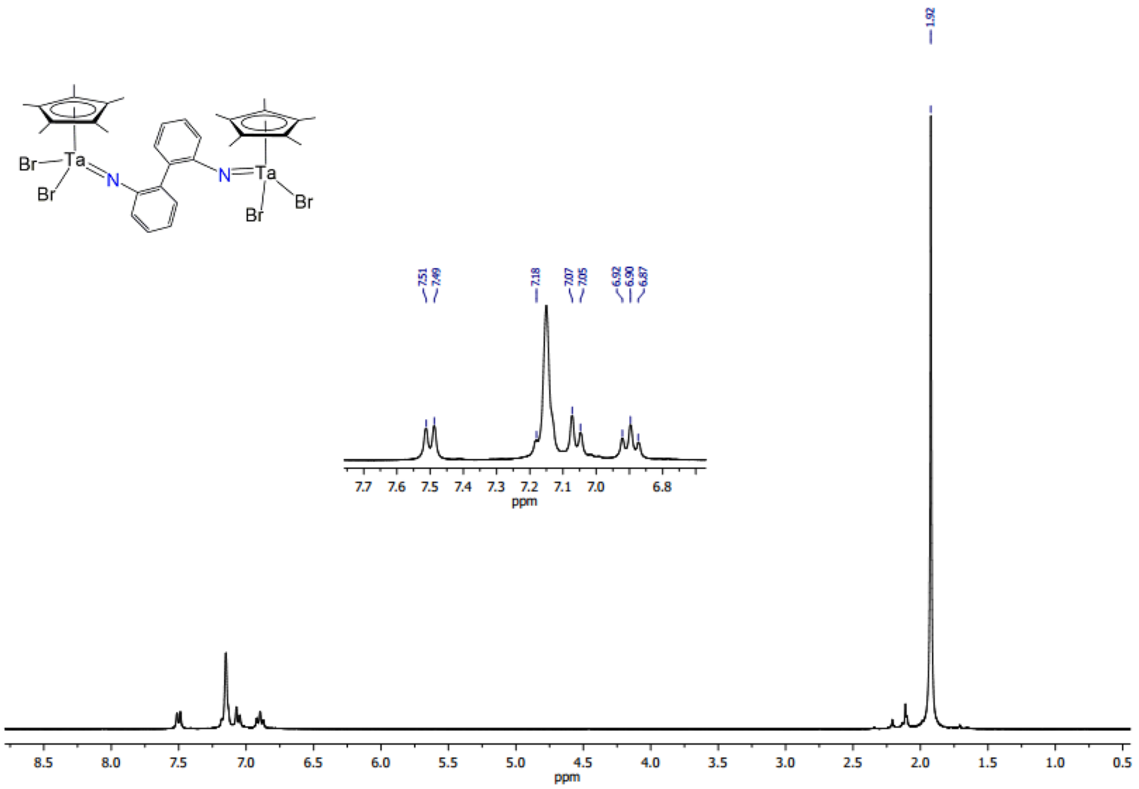

Figure S40. ^{1}H NMR spectrum of compound **7Br** in C_6D_6 (500 MHz).

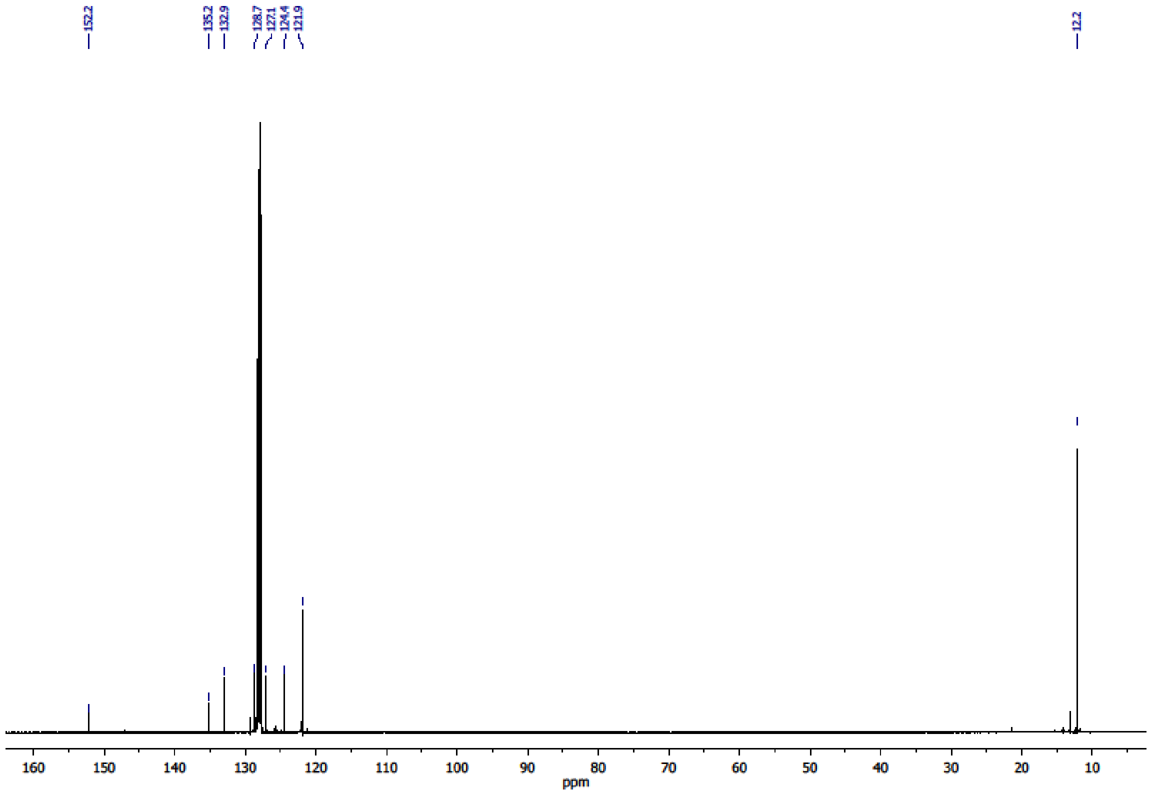

Figure S41. $^{13}C\{^{1}H\}$ NMR spectrum of compound **7Br** in C_6D_6 (125 MHz).

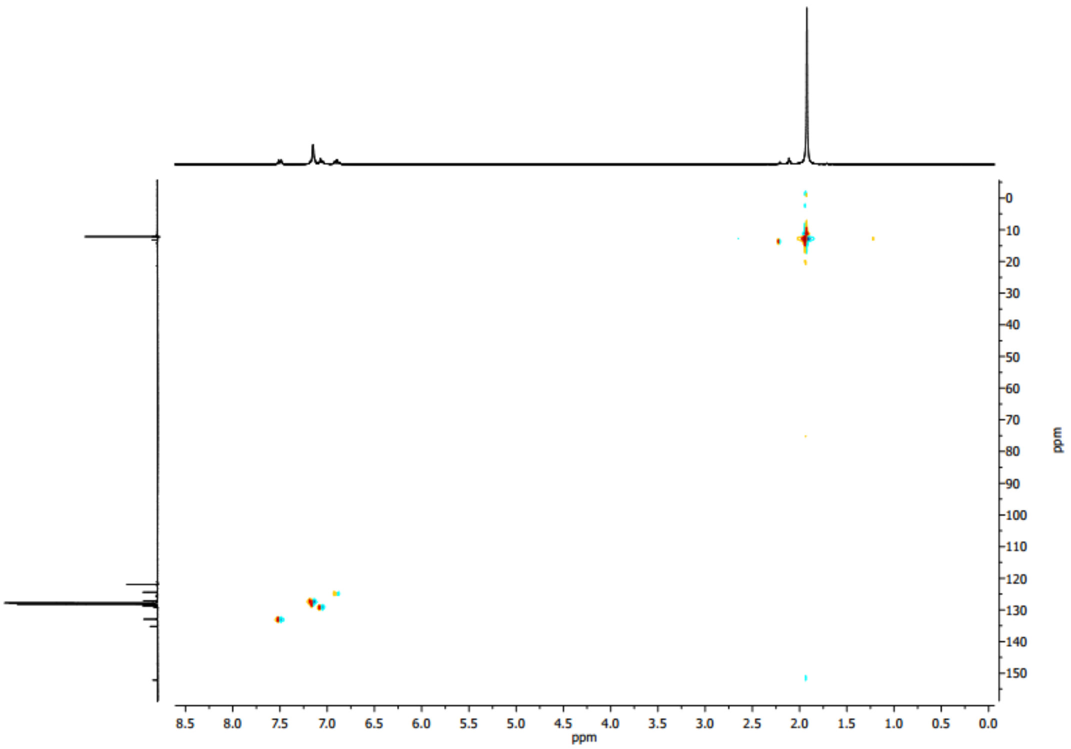

Figure S42. g-HSQC NMR spectrum of compound **7Br** in C_6D_6 (500 MHz).

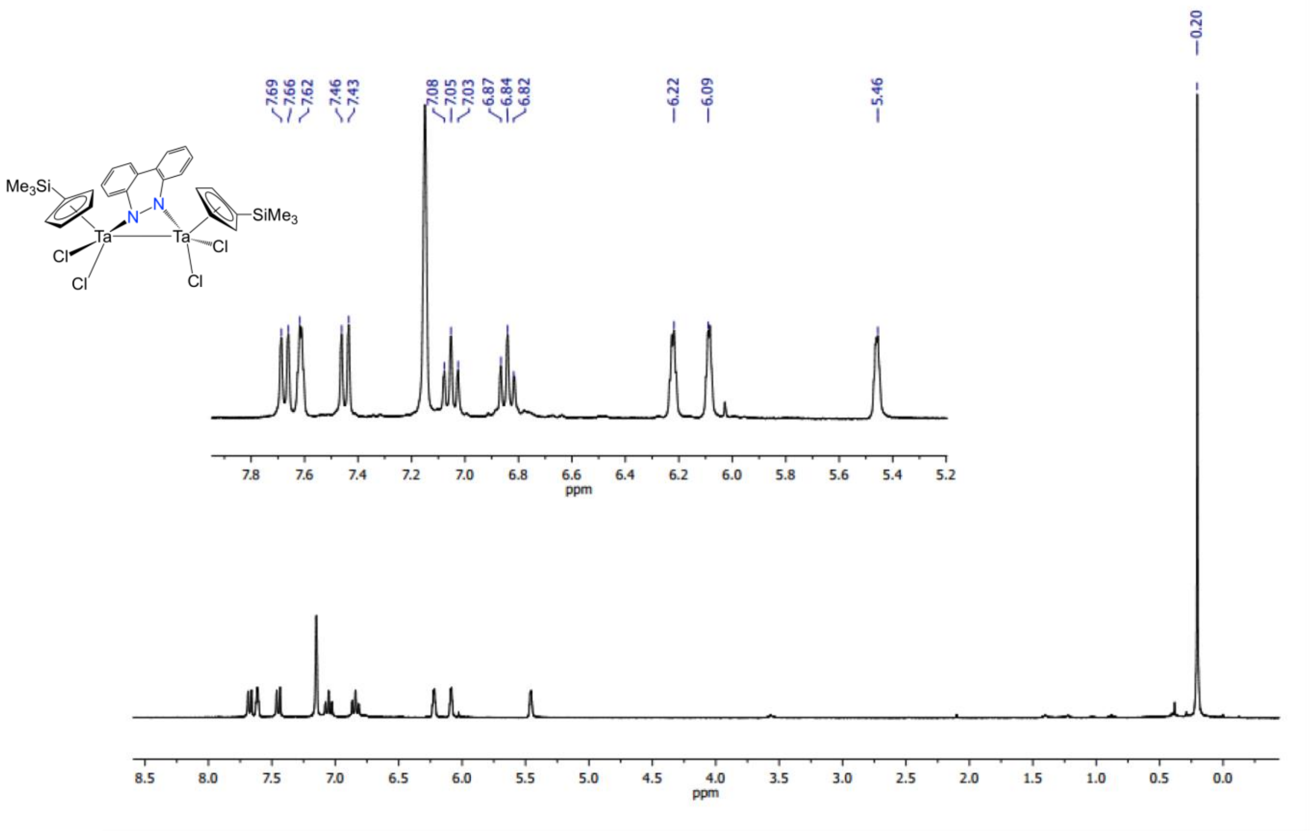

Figure S43. ^{1}H NMR spectrum of compound **8** in C_6D_6 (500 MHz).

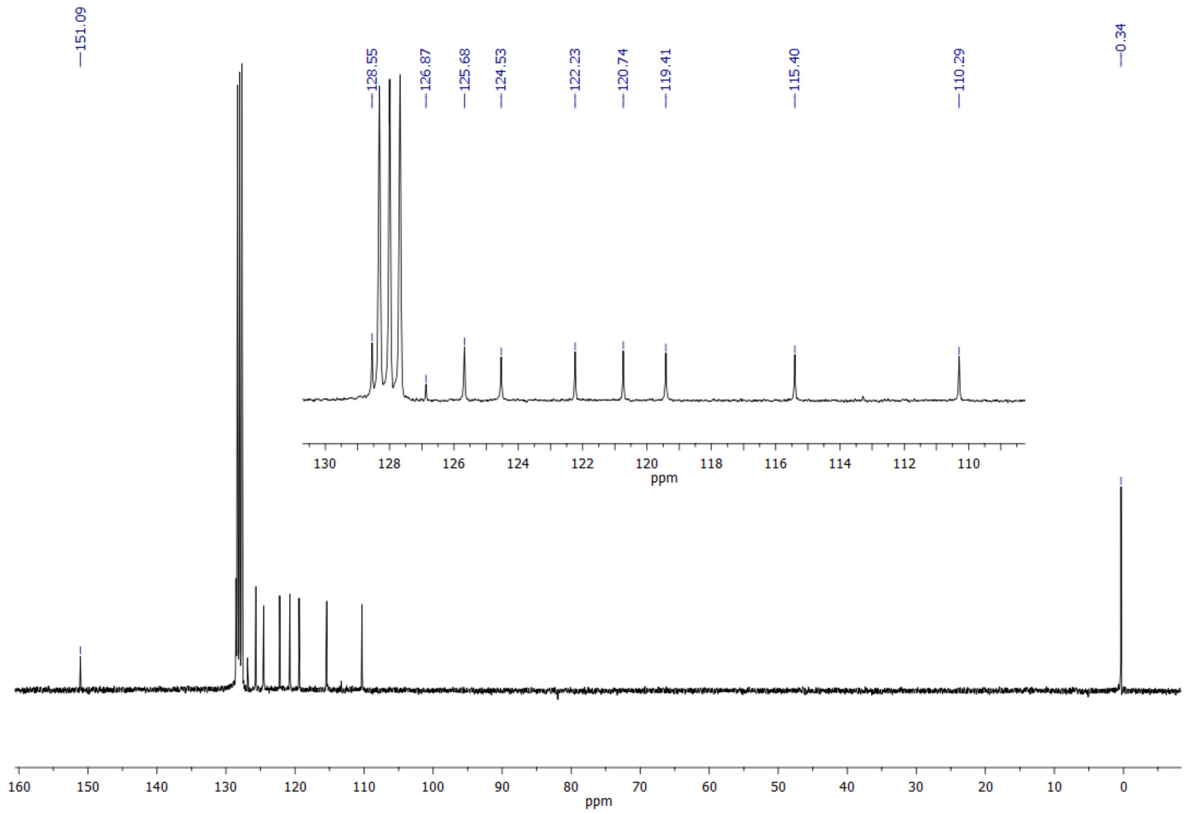

Figure S44. ^{13}C NMR spectrum of compound **8** in C_6D_6 (125 MHz).

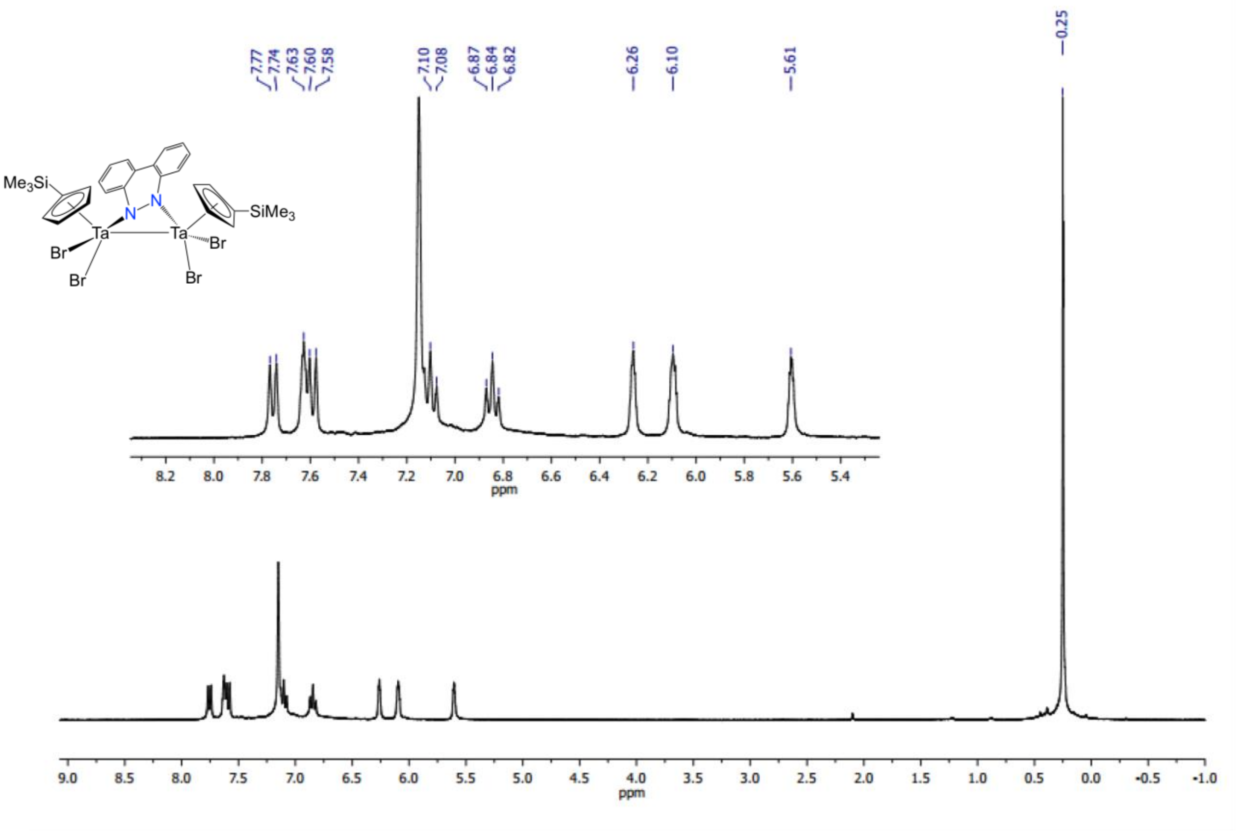

Figure S45. ^{1}H NMR spectrum of compound **8Br** in C_6D_6 (300 MHz).

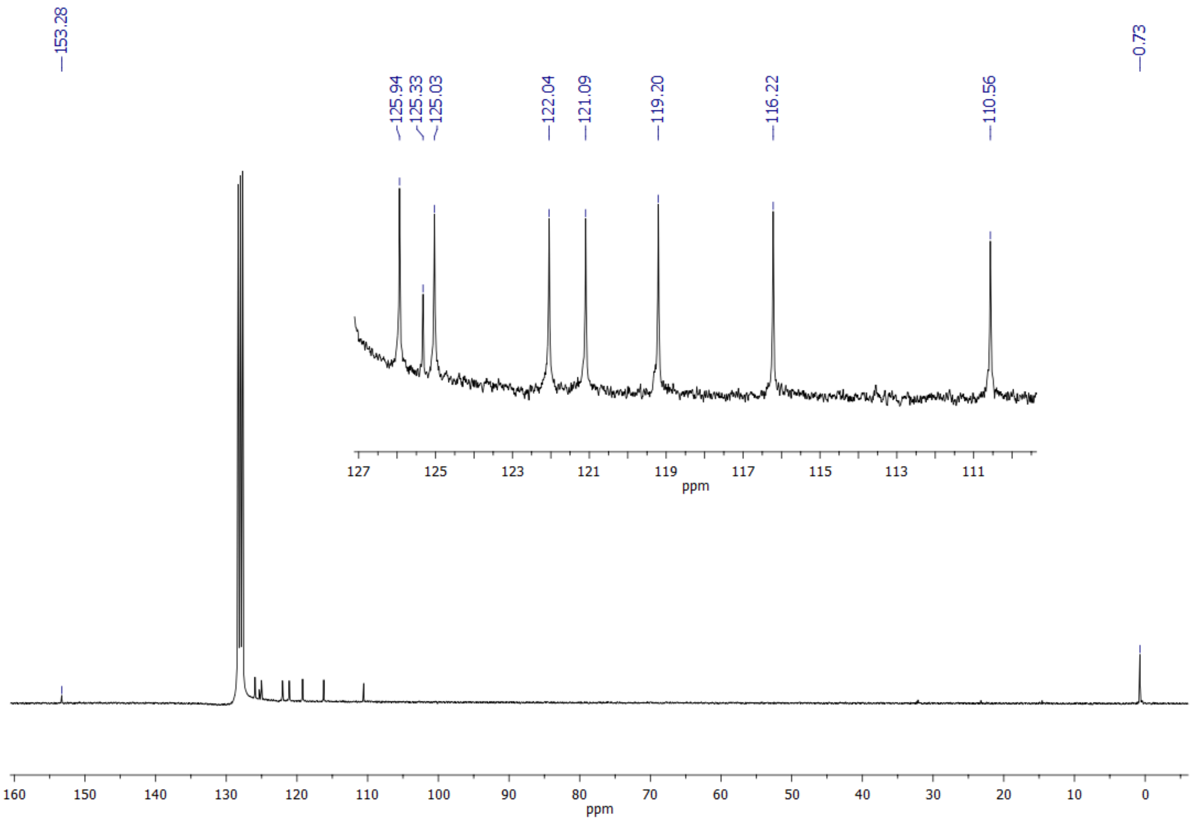

Figure S46. ^{13}C NMR spectrum of compound **8Br** in C_6D_6 (75 MHz).

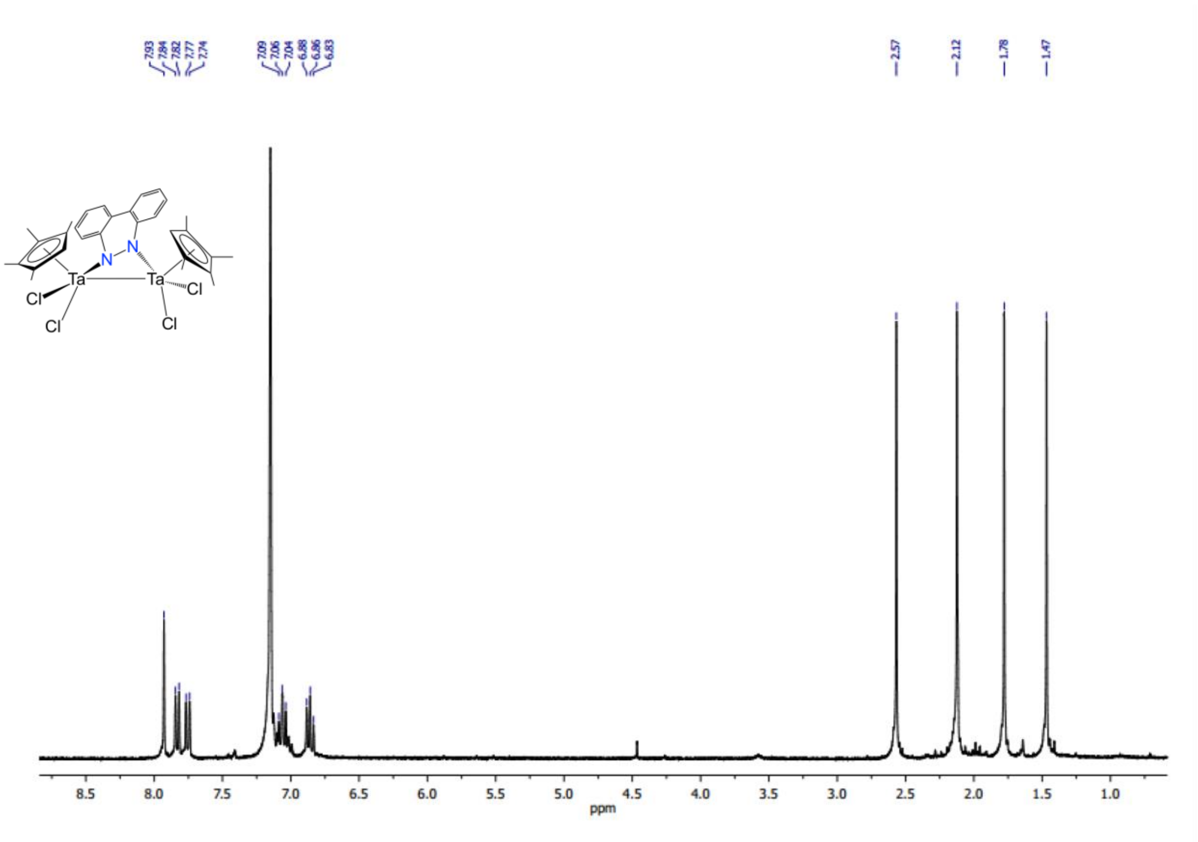

Figure S47. ^{1}H NMR spectrum of compound **9** in C_6D_6 (500 MHz).

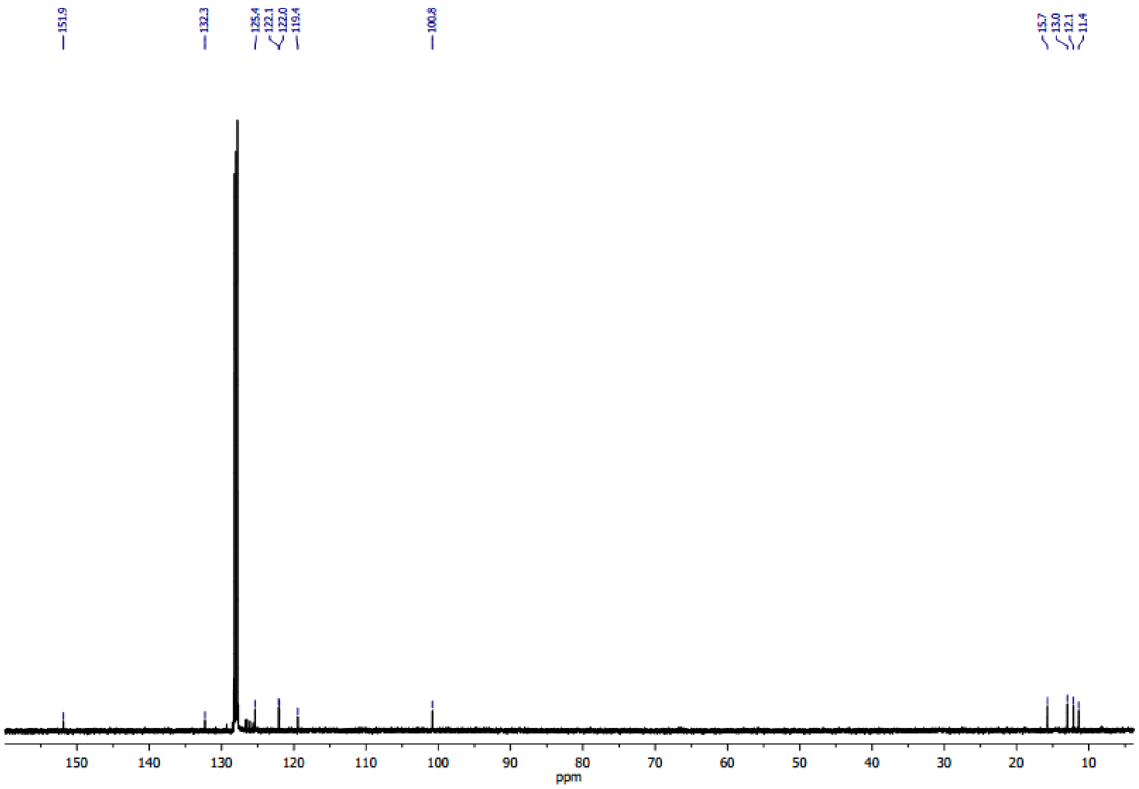

Figure S48. ^{13}C NMR spectrum of compound **9** in C_6D_6 (125 MHz).

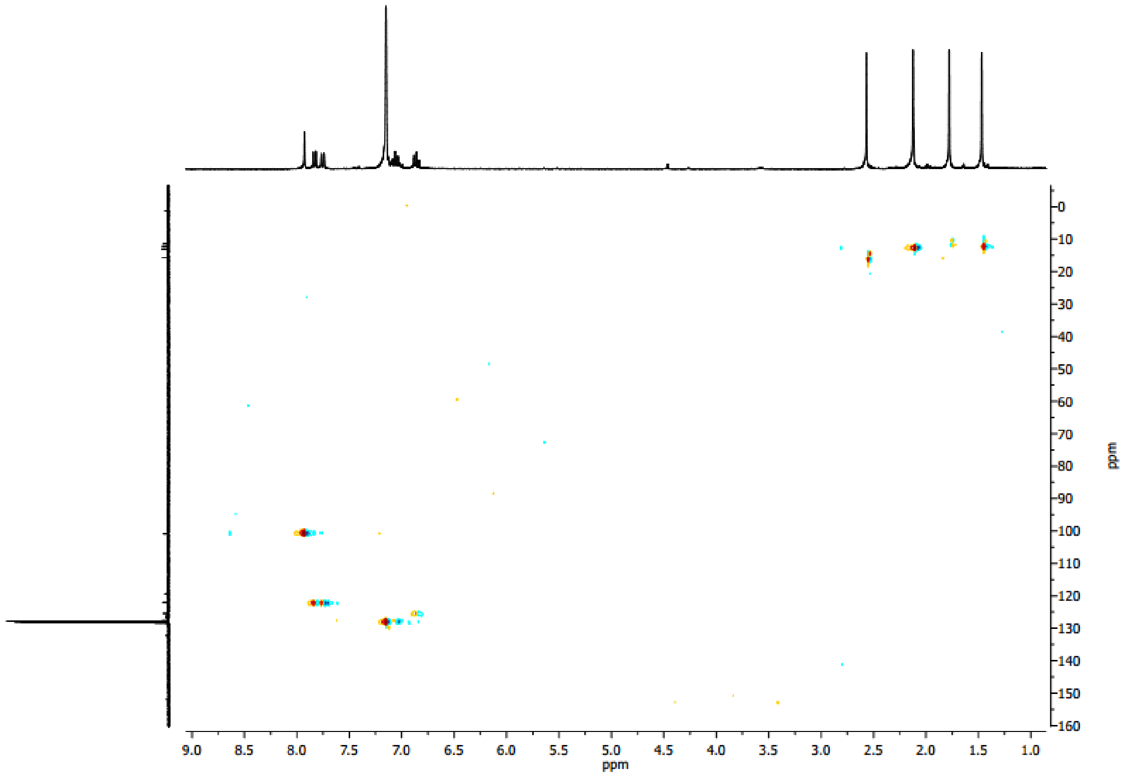

Figure S49. g-HSQC NMR spectrum of compound **9** in C_6D_6 (500 MHz).

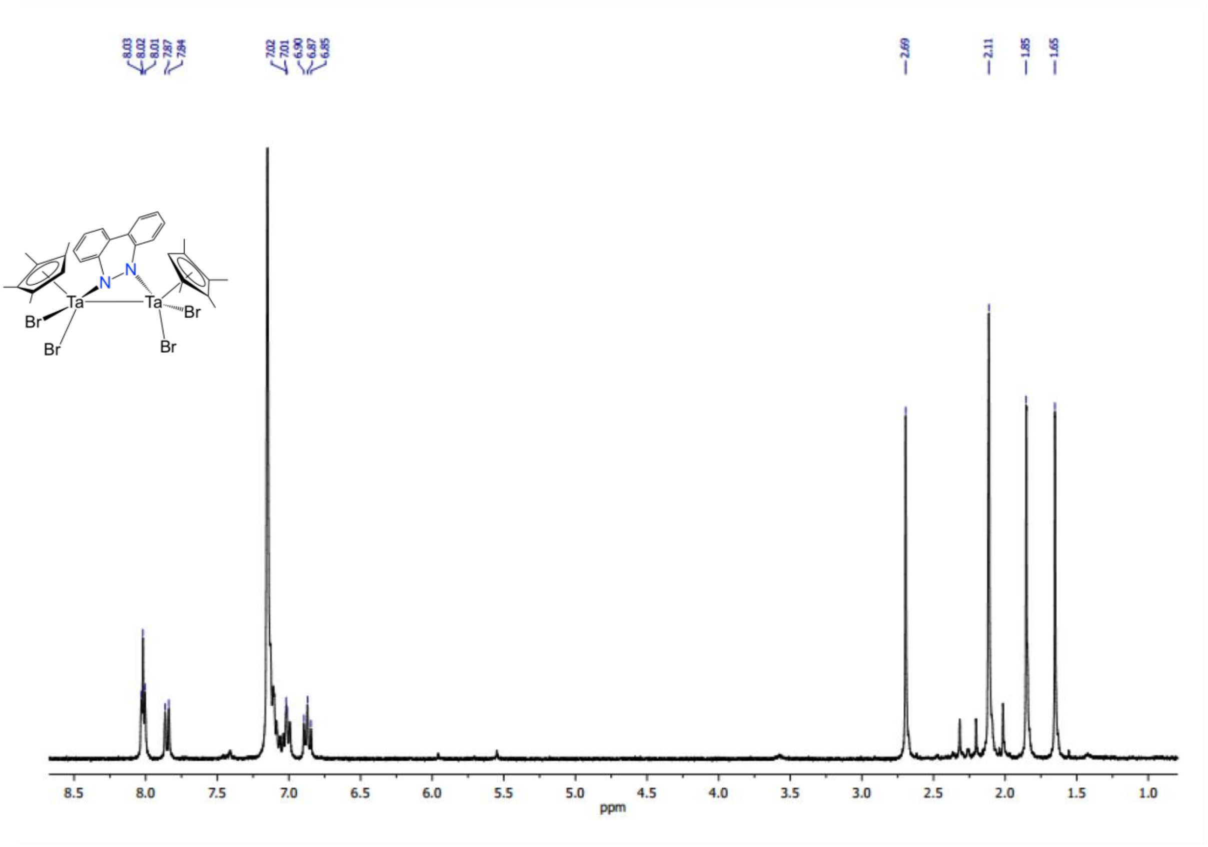

Figure S50. ^{1}H NMR spectrum of compound **9Br** in C_6D_6 (300 MHz).

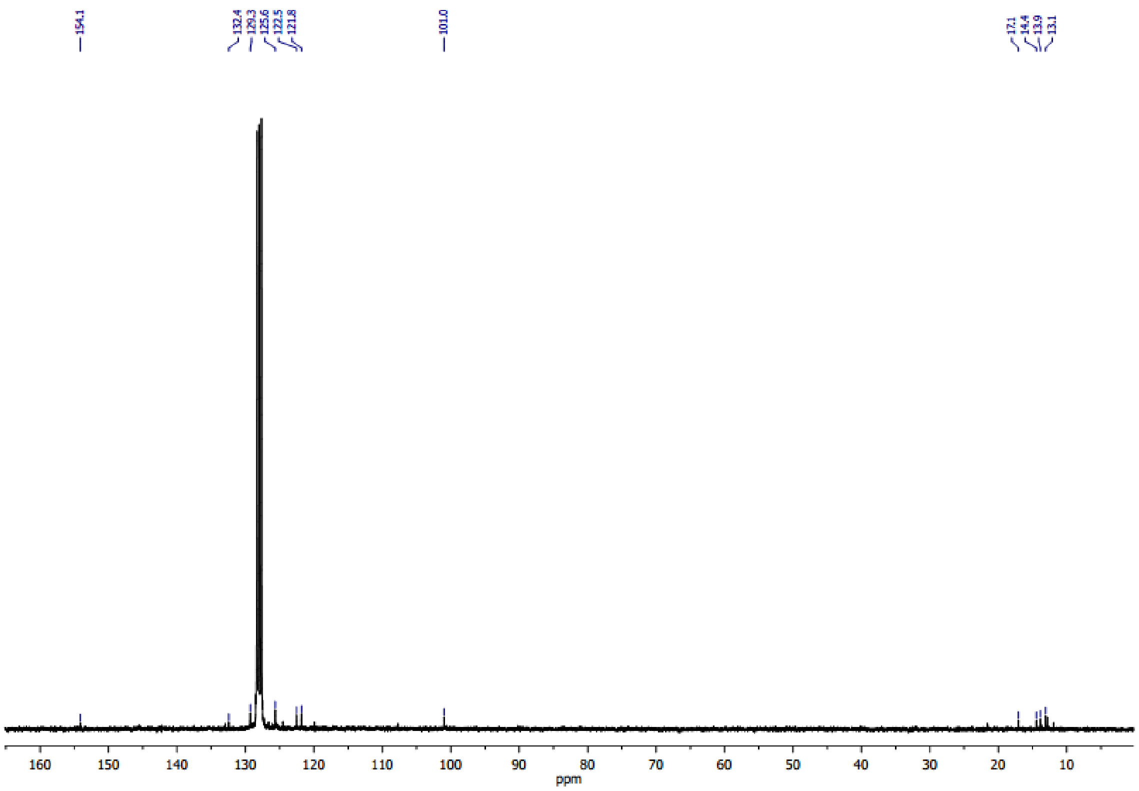

Figure S51. ^{13}C NMR spectrum of compound **9Br** in C_6D_6 (75 MHz)**.**

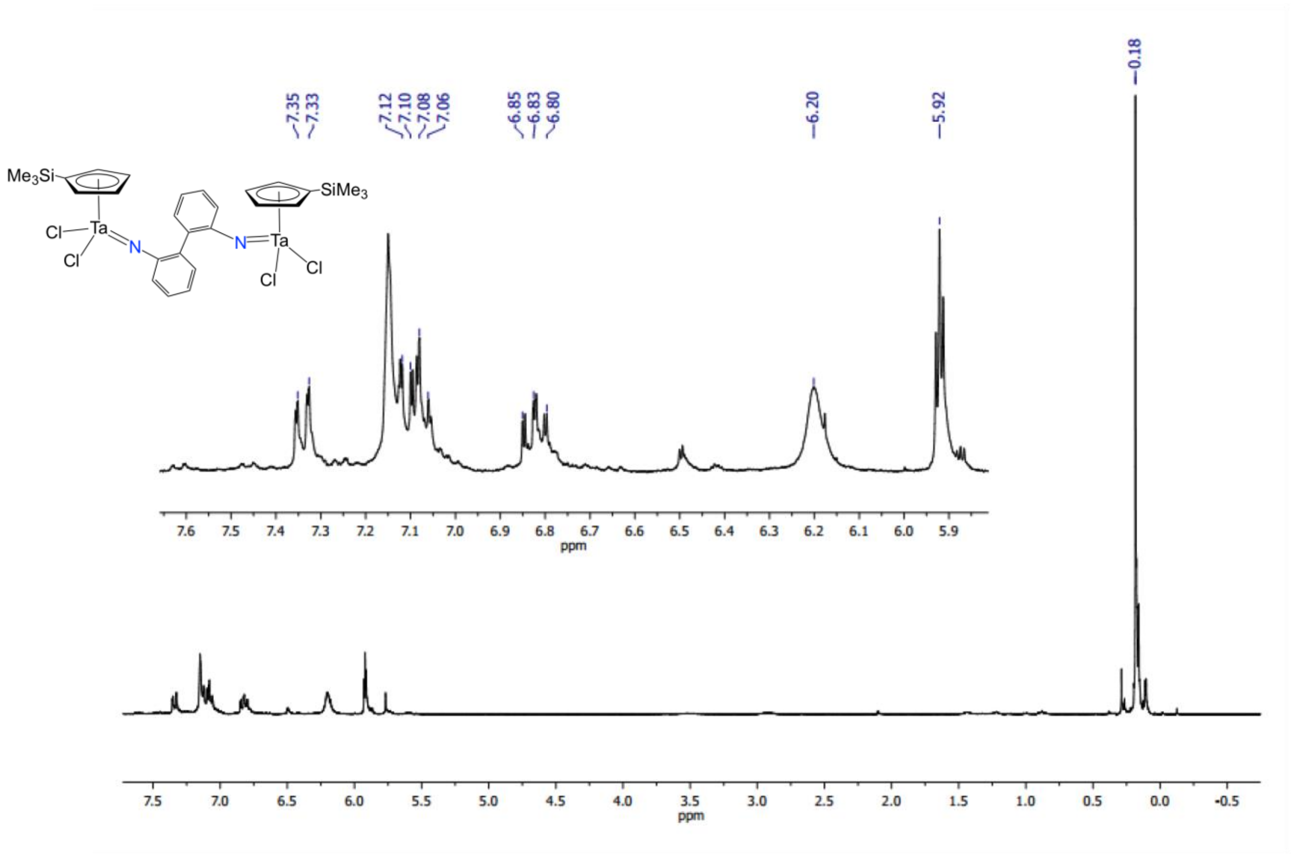

Figure S52. ^{1}H NMR spectrum of compound **10** in C_6D_6 (500 MHz).

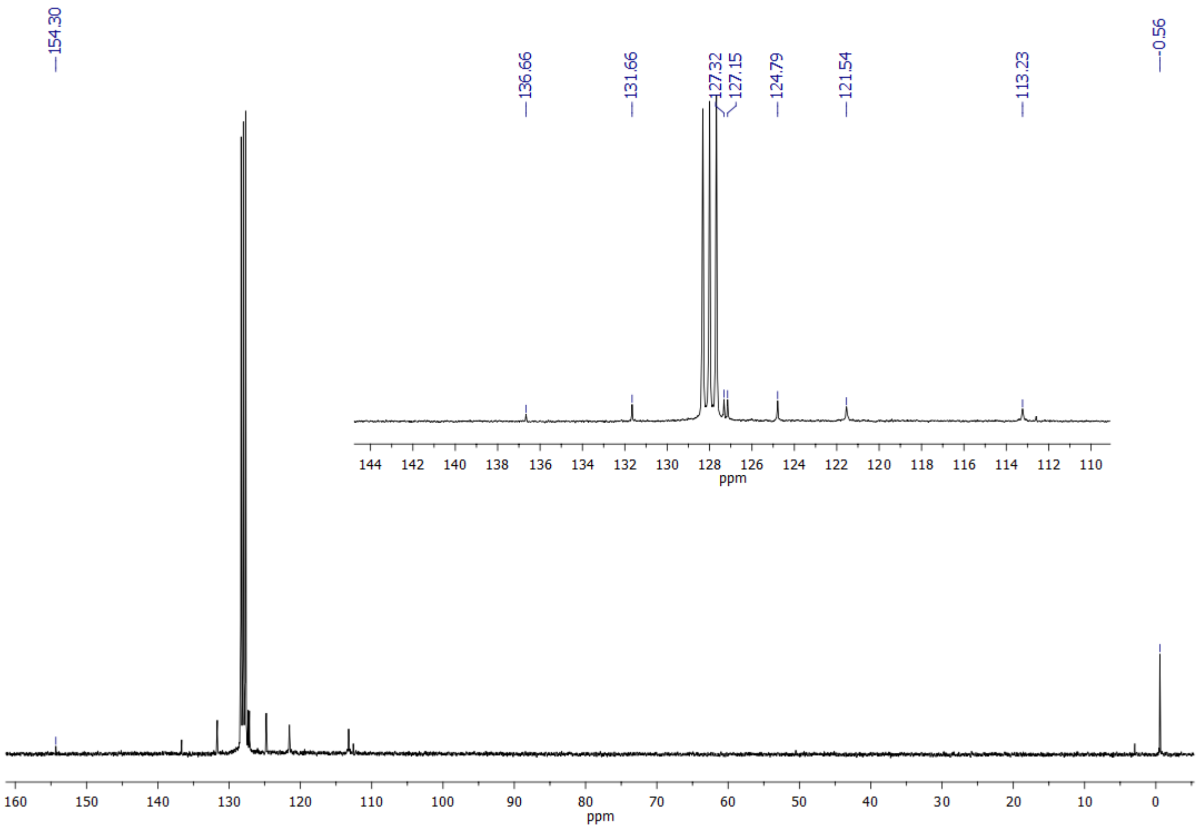

Figure S53. $^{13}C\{^{1}H\}$ NMR spectrum of compound **10** in C_6D_6 (125 MHz).

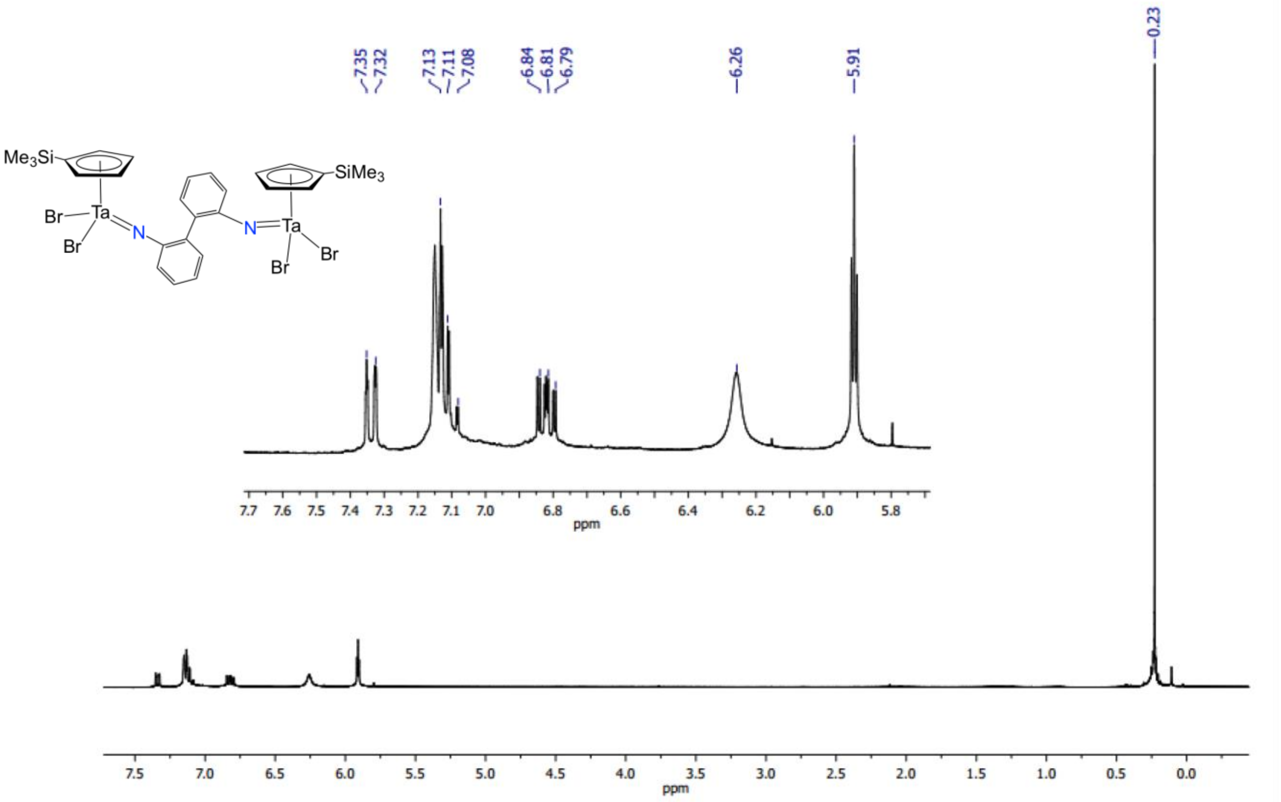

Figure S54. ^{1}H NMR spectrum of compound **10Br** in C_6D_6 (500 MHz).

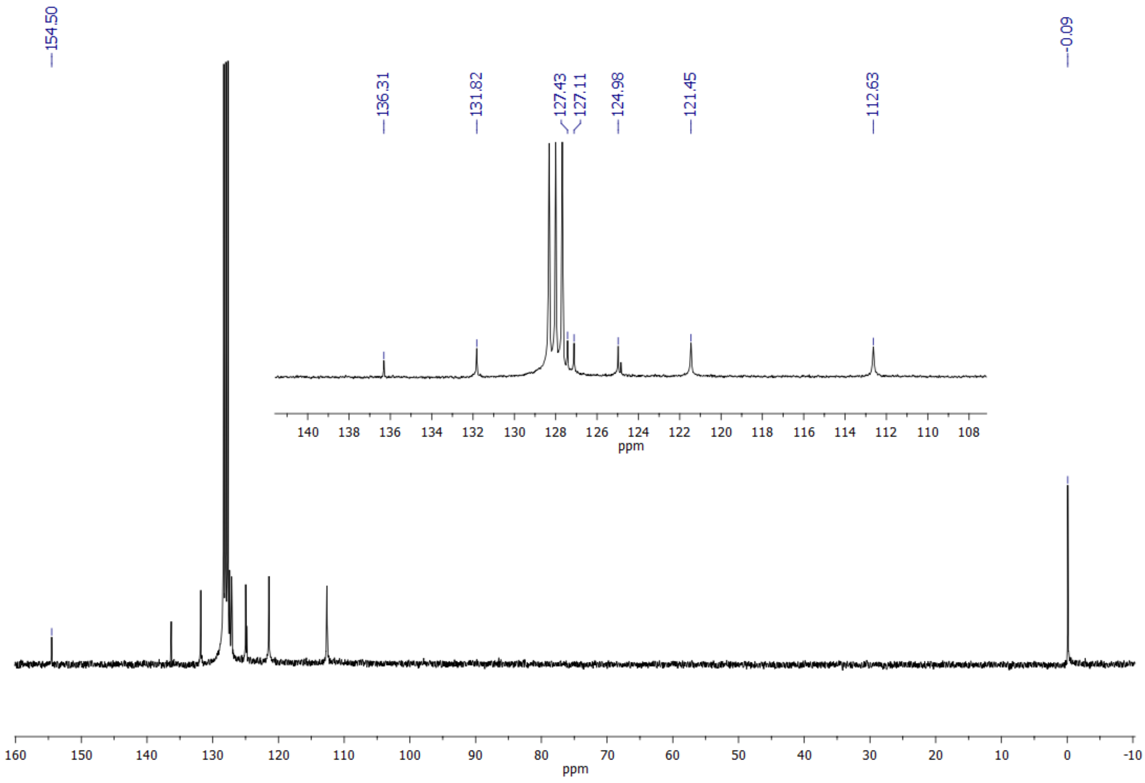

Figure S55. $^{13}C\{^1H\}$ NMR spectrum of compound **10Br** in C_6D_6 (125 MHz).

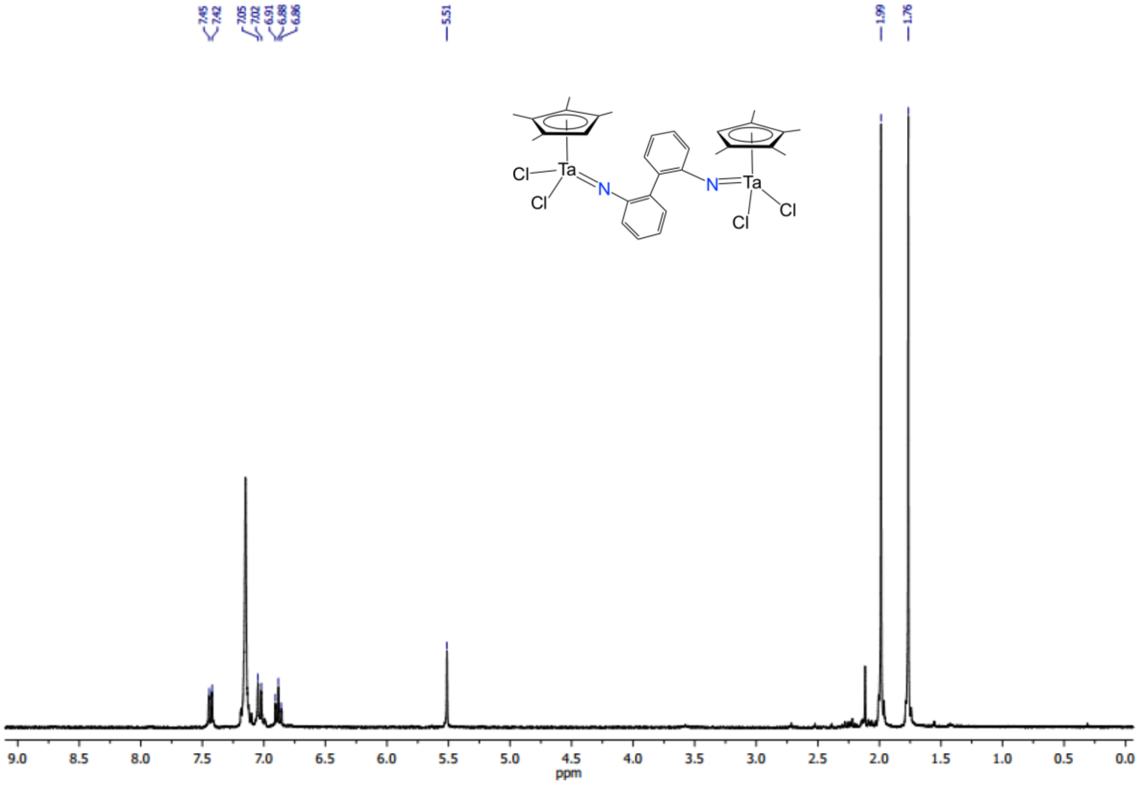

Figure S56. ^1H NMR spectrum of compound **11** in C_6D_6 (300 MHz).

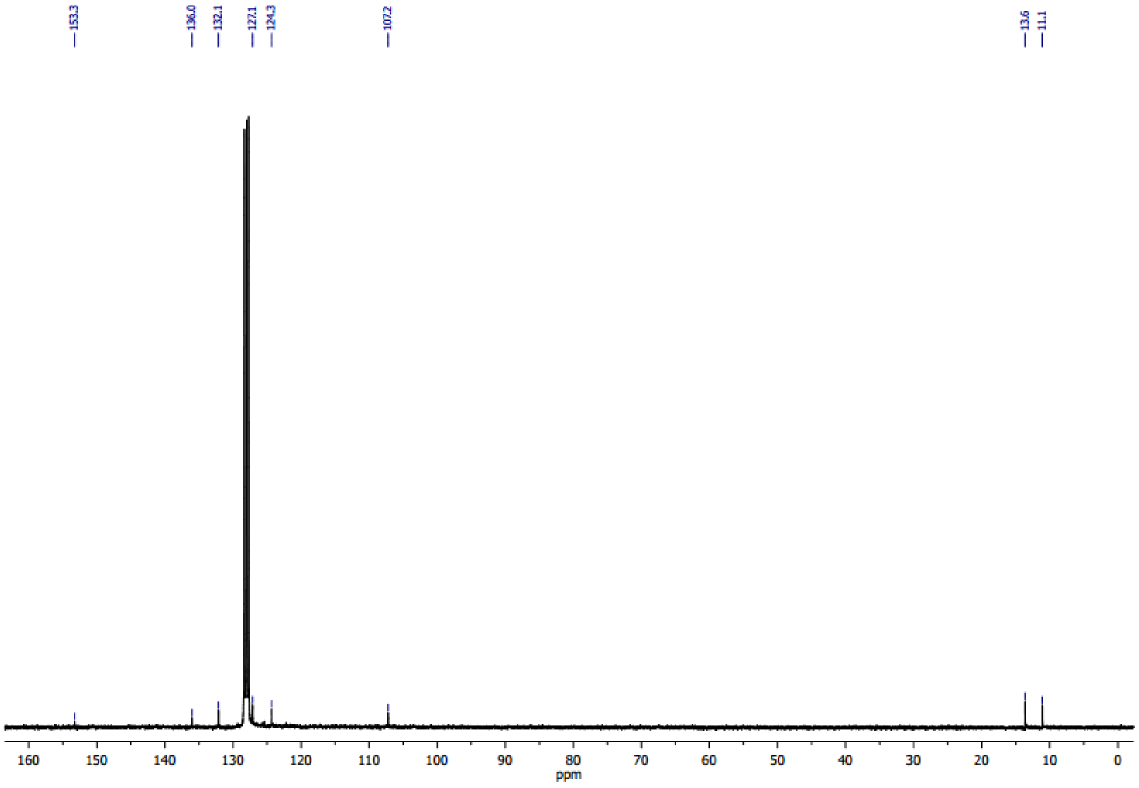

Figure S57. ^{13}C NMR spectrum of compound **11** in C_6D_6 (75 MHz).

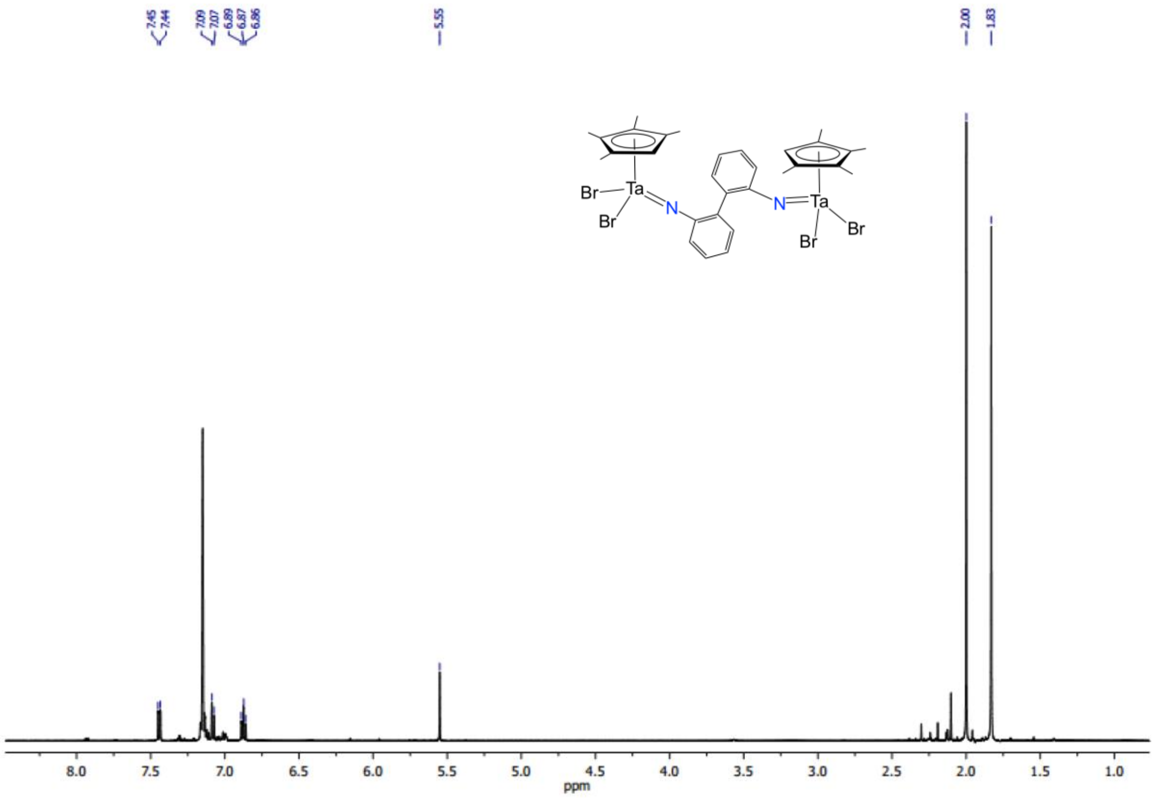

Figure S58. ^{1}H NMR spectrum of compound **11Br** in C_6D_6 (500 MHz).

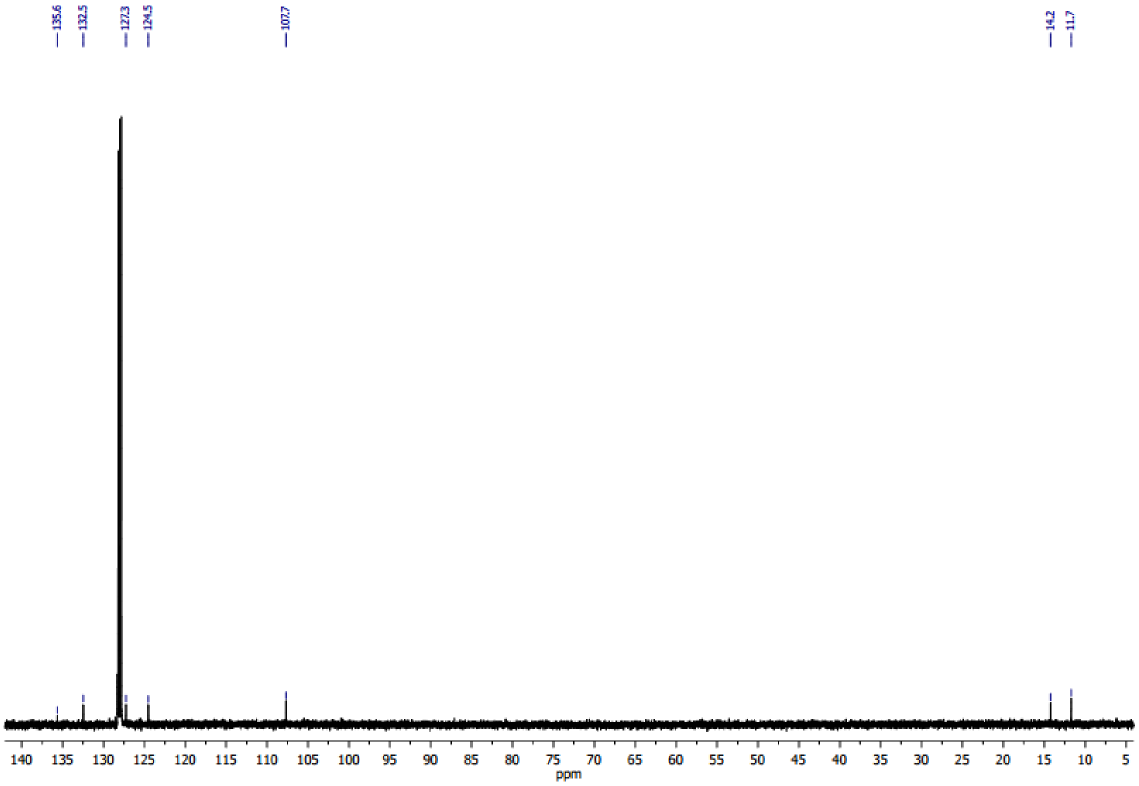

Figure S59. ^{13}C NMR spectrum of compound **11Br** in C_6D_6 (125 MHz).

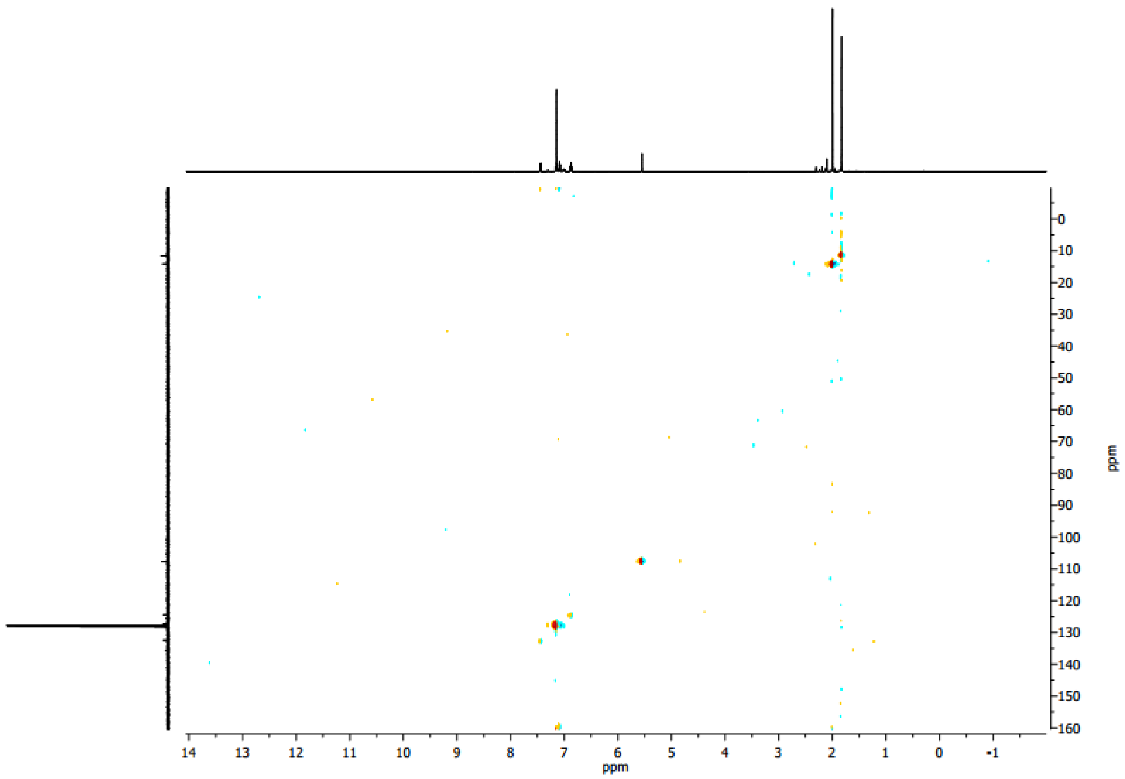

Figure S60. g-HSQC NMR spectrum of compound **11Br** in C_6D_6 (500 MHz).

Theoretical Studies

Table S2. X-Ray diffraction vs Calculated geometrical parameters for complex **1** and **2** and their analogous with Br, **1Br, 2Br** and **3Br**. Distances are in Å and angles in deg.

	**1**		**1Br**		**2**		**2Br**		**3Br**	
	**X-ray**	**Calc.**	**X-ray**	**Calc.**	**X-ray**	**Calc.**	**X-ray**	**Calc.**	**X-ray**	**Calc.**
**Ta-Ta**	2.813(1)	2.78	2.840(2)	2.80	2.758(1)	2.72	2.753(2)	2.73	2.764(1)	2.75
**Ta-H1**	2.0(2)	1.84	1.960(1)	1.84	1.859(1)	1.86	1.86(2)	1.85	1.84(1)	1.85
**Ta-H1a/2**	1.8(2)	1.84	1.621(1)	1.84	1.656(1)	1.86	1.66(2)	1.85	1.85(1)	1.85
**Ta-Cl/Br**	2.36(1)	2.37	2.510(1)	2.54	2.354(9)	2.37	2.502(6)	2.54	2.503(8)	2.52<br>2.55
**Ta-H1-Ta**	97(2)	98	104.5(1)	99	103.2(1)	94	102.5(5)	95	96.9(7)	96
**Cl1/Br1-Ta-Cl2/Br2**	100.0(2)	101	99.3(1)	99	101.5(3)	104	101.0(5)	104	101.5(1)	103

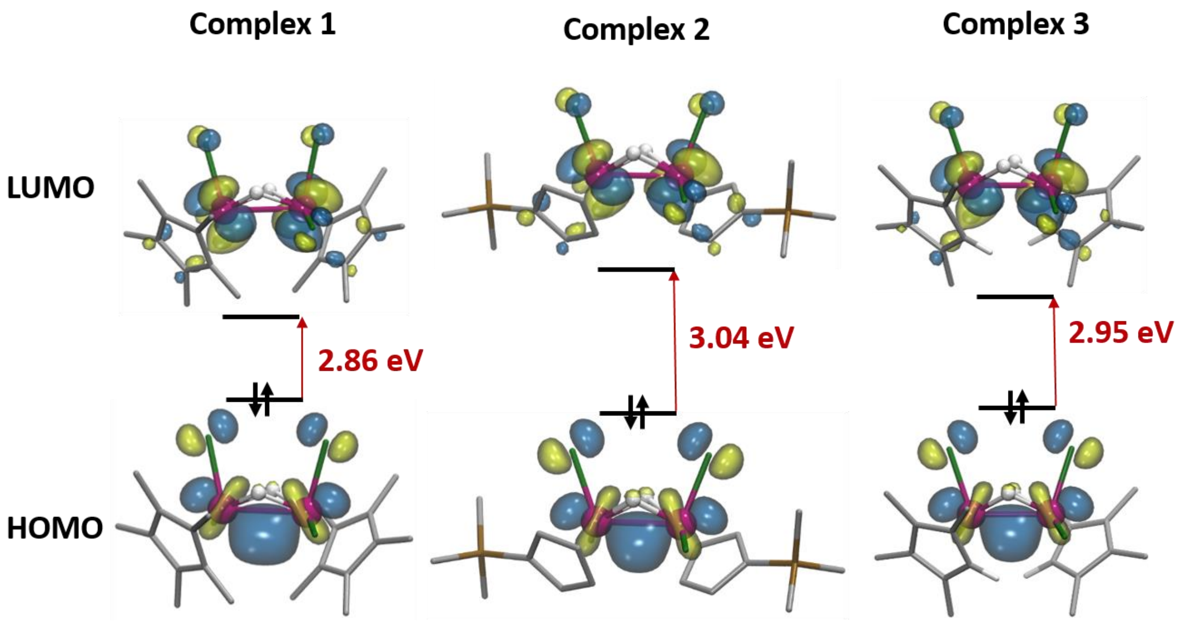

Figure S61. Frontier Molecular Orbitals of complexes **1**, **2** and **3**; and HOMO-LUMO energy gaps.

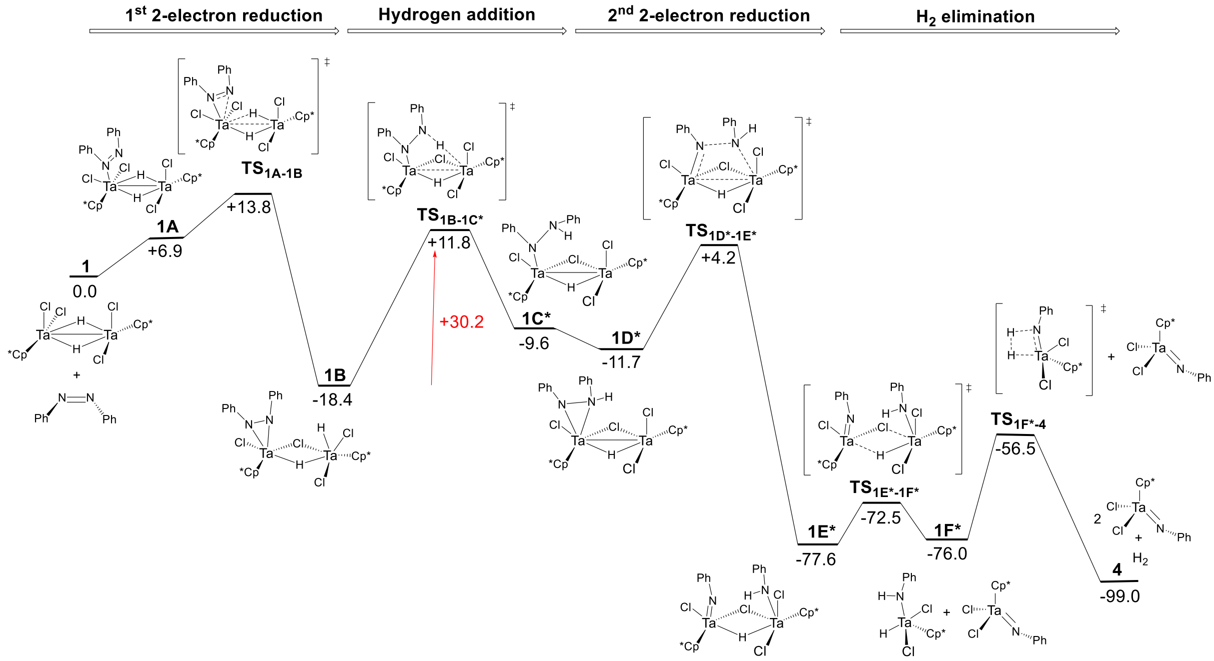

Figure S62. Alternative N=N double bond breaking mechanism for the transformation of **1** into **4**. Gibbs free energies in kcal·mol^{-1}.Although the overall free energy barrier is only somewhat higher (1 kcal mol^{-1}) than that of the proposed mechanism in Figure 5, we discarded this mechanism because it does not involve the formation of an intermediate analogous to experimentally isolated dinuclear μ-(η^2,η^2)-benzo[c]cinnoline complexes (**10** and **11**).

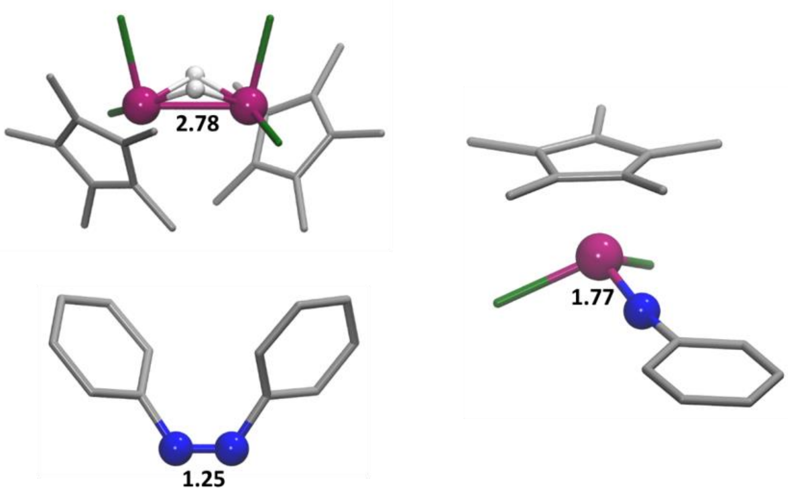

1 **4**

Figure S63. DFT structures of reactants and product **4**. Distances in Å. Hydrogens are omitted for clarity.

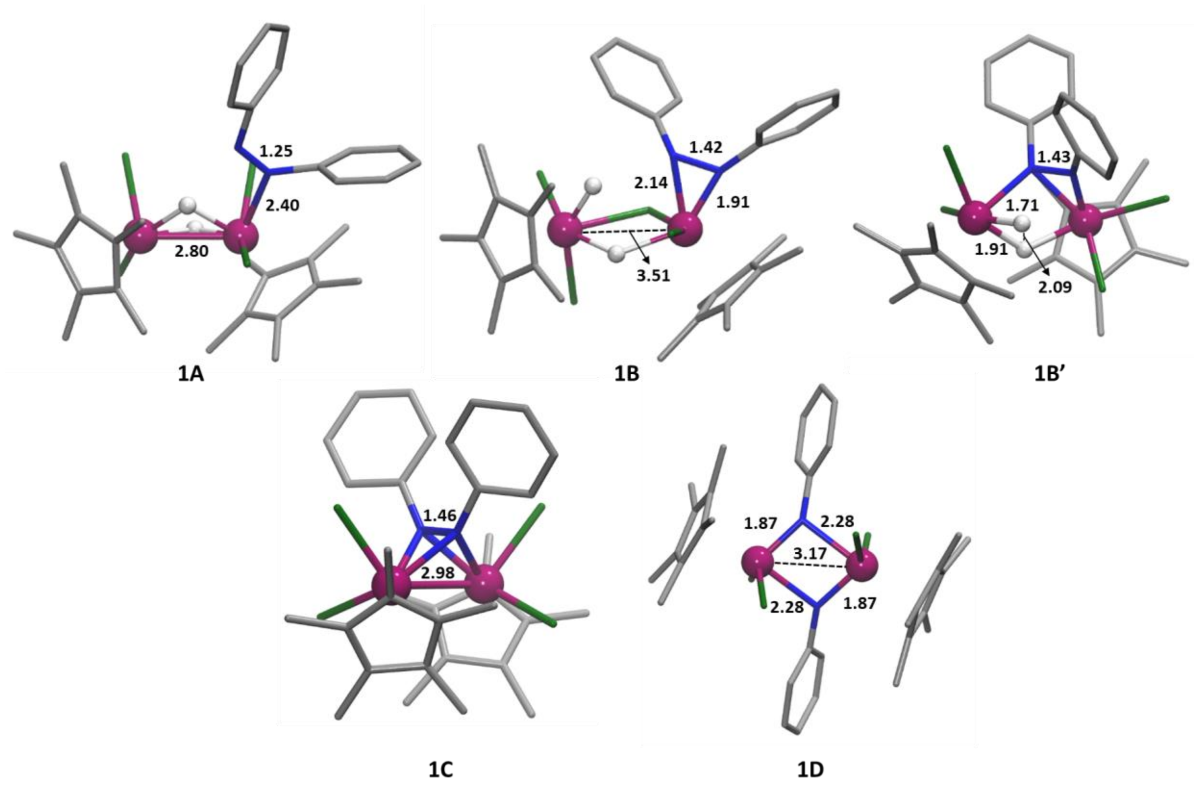

Figure S64. DFT structures of different reaction intermediates of the mechanism shown in Figure 5. Selected distances in Å. Hydrogens are omitted for clarity.

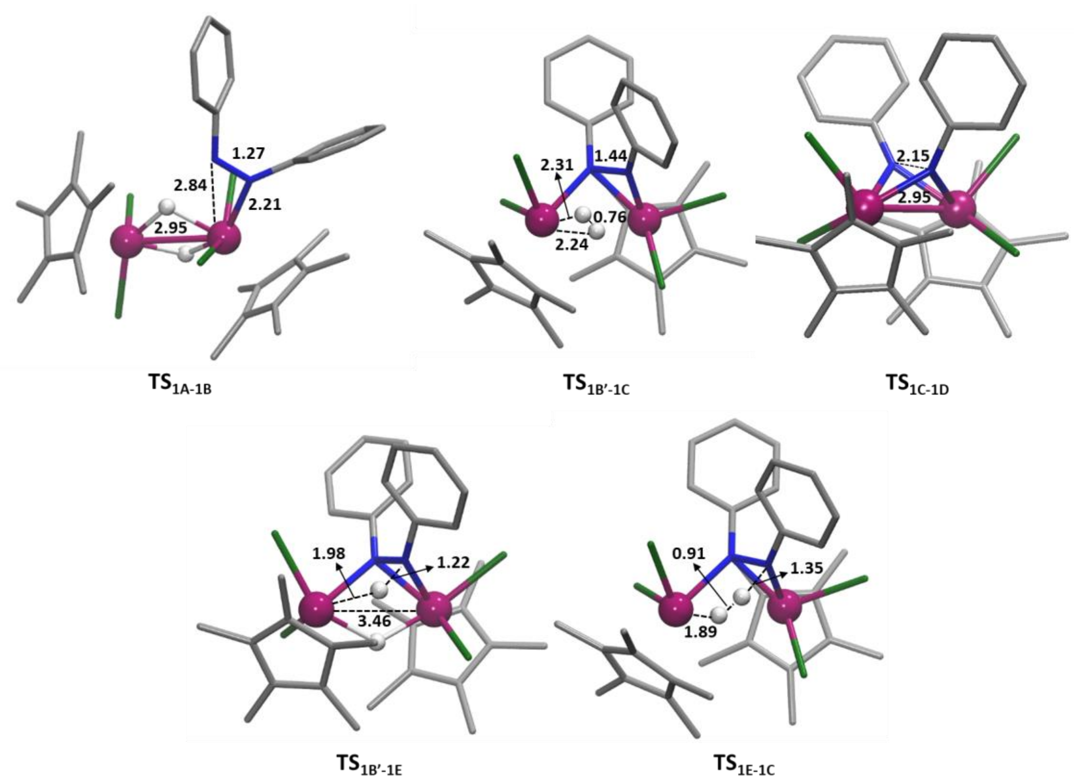

Figure S65. DFT structures of the transition states of the mechanism shown in Figure 5. Selected distances in Å. Hydrogens are omitted for clarity.
